# Micro RNAs upregulated in Vitiligo skin play an important role in its aetiopathogenesis by altering TRP1 expression and keratinocyte-melanocytes cross-talk

**DOI:** 10.1038/s41598-019-46529-6

**Published:** 2019-07-12

**Authors:** Utpreksha Vaish, Avinash A. Kumar, Swati Varshney, Shreya Ghosh, Shantanu Sengupta, Chandni Sood, Hemanta K. Kar, Pankaj Sharma, Vivek T. Natarajan, Rajesh S. Gokhale, Rajni Rani

**Affiliations:** 1 National Institute of Immunology, New Delhi, 110067 India; 2 CSIR-Institute of Genomics & Integrative Biology, Mathura Road, Sukhdev Vihar, New Delhi, 110025 India; 3 Academy of Scientific and Innovative Research (AcSIR), CSIR-IGIB, Mathura Road, Sukhdev Vihar, New Delhi, 110025 India; 4 Dr. Ram Manohar Lohia Hospital, New Delhi, 110001 India

**Keywords:** High-throughput screening, Protein-protein interaction networks, miRNAs

## Abstract

Translation of genes is regulated by many factors including microRNAs (miRNAs). miRNA profiling of lesional and non-lesional epidermal RNA from 18 vitiligo patients revealed significant upregulation of 29 miRNAs in the lesional epidermis, of which 6 miRNAs were transfected in normal human epidermal keratinocytes (NHEKs) to study their downstream effects using quantitative proteomics. Many proteins involved in oxidative stress, Vesicle trafficking, Cellular apoptosis, Mitochondrial proteins and Keratins were regulated after miRNA transfections in the keratinocytes. However, tyrosinase related protein-1 (TRP1/TYRP1), a melanogenesis protein, was consistently downregulated in NHEKs by all the six miRNAs tested, which was quite intriguing. TRP1 was also downregulated in lesional epidermis compared with non-lesional epidermis. Since melanocytes synthesize and transfer melanosomes to the surrounding keratinocytes, we hypothesized that downregulation of TRP1 in NHEKs may have a role in melanosome transfer, which was confirmed by our co-culture experiments. Downregulation of TRP1 in keratinocytes negatively affected the melanosome transfer from melanocytes to keratinocytes resulting in melanin accumulation which may be leading to melanin induced cytotoxicity in melanocytes. Regulation of key processes involved in aetiopathogenesis of vitiligo along with TRP1 suggests that miRNAs act in an integrated manner which may be detrimental for the loss of melanocytes in vitiligo.

## Introduction

Translation of genes into functional proteins is regulated by many factors including MicroRNAs (miRNAs). Micro RNAs are small endogenous, non-coding gene regulatory RNAs that are involved in post transcriptional regulation of gene expression^1–3^. They are approximately 21–23 nucleotides long, evolutionarily conserved RNA molecules which inhibit gene expression by affecting mRNA stability or post-transcriptional repression by base pairing with the “seed sequences” in 3′ UTR or 5′ UTR^4,5^ of the gene. Each miRNA has multiple potential targets, since the seed match requires only 4–7 base complementarity^6^. miRNAs constitute about 1–5% of human genome and are predicted to regulate more than 60% of protein coding genes^1,5,7^. They have been reported to regulate several processes like cell proliferation, differentiation, signal transduction and Immune responses etc^8,9^. miRNA biogenesis involves a sequential series of both nuclear and cytoplasmic cleavage events performed by ribonuclease III endonucleases, Drosha and Dicer^7^, ultimately leading to miRNA guided regulation via RNA-induced silencing complex (RISC) which induces gene silencing by either mRNA cleavage or translational blockage^7^. Several miRNAs have been implicated in skin diseases and pigmentation. Micro RNA(miR)-434-5p homologue, miR-330-5p, miR-125, miR-145 and miR-203 have been shown to target major pigmentation genes like *Tyrosinase*^10,11^, *MITF*^12^, *Sox9*, *Mitf*, *Tyr*, *Trp1*, *Myo5a*, *Rab27a* and *Fscn1*^13^. Further, miR-203, identified as a master regulator of epidermal differentiation has been shown to restrict stemness by suppressing expression of the stem cell marker p63 and to effect melanogenesis via melanosome transport mechanisms^14^. While these studies have implicated the role of miRNAs in skin pigmentation^10,12–14^, there are very few studies on micro RNA profiling of lesional and non-lesional epidermis of vitiligo patients. One study selected 12 well described micro RNAs and compared their expression between the vitiligo and control skin^15^, and reported that miR-99b, miR-155, miR-199a-3p, miR-125b and miR-145 were dysregulated in the skin of vitiligo patients. Of these miRNAs, miR-155, was found to cause inhibition of melanogenesis associated genes in the skin like *TRP*, *SDCBP*, *YWHAE*, and *SOX10*^15^. Another study on four vitiligo patients from the Indian subcontinent reported 12 miRNAs to be significantly upregulated in the lesional skin of patients compared with healthy control skin and only miR-136, miR-296 and miR-238 had significantly decreased expression in the lesional skin compared with the non-lesional skin^16^. However, none of these studies compared the miRNA profile of paired lesional and non-lesional skin in an objective manner without any selection bias. So, we wanted to objectively explore the expression of miRNAs in lesional and non-lesional skin of eighteen vitiligo patients and study their role, if any, in manifestation of vitiligo.

Vitiligo is a depigmenting disorder where functional melanocytes are lost, resulting in milky white patches on the skin. While the mechanism of melanocyte loss in vitiligo has not been clearly understood, autoimmunity has been widely implicated. However, it is not clear whether autoimmunity is the cause or effect of depigmentation in vitiligo. In this study, we have attempted to study the alterations observed in the “miRNome” of the lesional epidermis compared to the non-lesional epidermis, and tried to decipher the role of altered miRNA expressions in the pathogenesis of vitiligo. In the normal epidermis, melanocytes and keratinocytes are present at a ratio of 1:40 constituting the epidermal melanin unit, where melanocytes synthesize and transfer melanosomes to the surrounding keratinocytes to save them from UV induced damage^17,18^. Keratinocytes, on the other hand, produce growth factors for melanocytes to proliferate and melanogenesis^19^. Thus these two important cell types play an important role in maintaining epidermal homeostasis. However, lesional epidermis of vitiligo lacks melanocytes and has keratinocytes only. While vitiligo is usually considered as the disease of melanocytes, we have recently shown widespread alterations in the lesional epidermis (that has keratinocytes only and no melanocytes), at architectural, cellular, and transcriptome levels when compared with paired non-lesional epidermis, which may be involved in protecting the keratinocytes against UV-induced damage, in the absence of melanin^20^. In the present study, to decipher the role of miRNAs upregulated in the lesional skin, we transfected normal human epidermal keratinocytes (NHEKs) with selected pre-miRNAs (since the lesional skin has only keratinocytes), followed by proteomic analysis in order to study altered proteome of NHEKs, which may shed some light on their role in aetiopathogenesis of vitiligo.

## Results

### Evaluation of differentially expressed micro RNAs (miRNAs) in lesional epidermis

**Figure 1:**
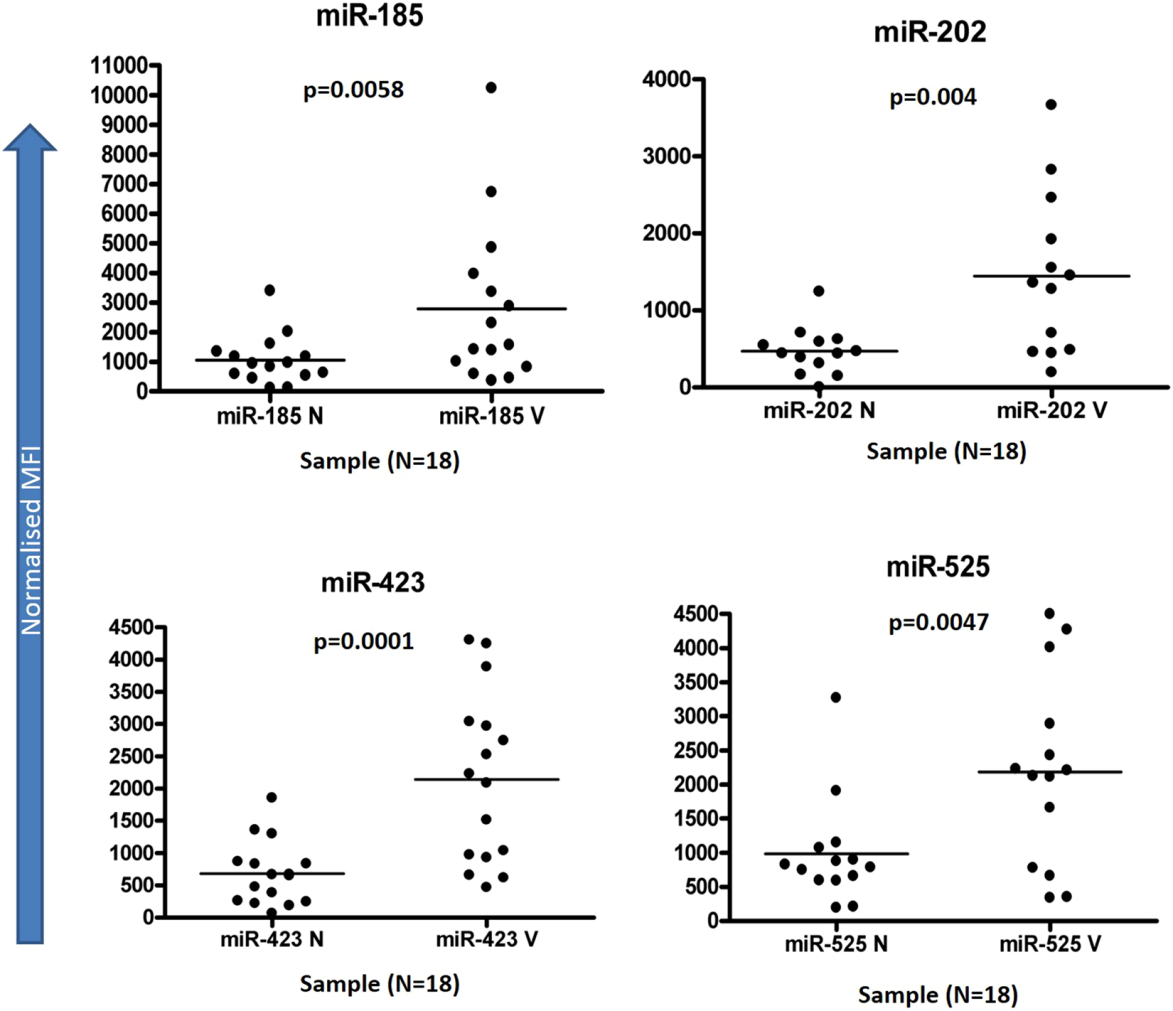
Expression of micro RNAs miR-185, miR-202, miR-423, & miR-525 in Vitiligo: Scatter-plots showing the expression of miR-185, miR-202, miR-423, and miR-525 in lesional and non-lesional epidermis of 18 vitiligo patients. On, X axis the suffix N denotes non-lesional epidermis, while V denotes the vitiligo lesional epidermis. Values plotted are the background-subtracted, normalised Mean Fluorescence Intensity (MFIs).

Lesional and non-lesional epidermal RNAs from 18 vitiligo patients were studied for the expression of 318 miRNAs using the FlexmiR MicroRNA Assay. After applying a cut off of 50 MFI (Mean Fluorescence Intensity) and calculating fold change, 56 miRNAs showed upregulation of more than 1.5 fold in the lesional skin. Of the 56 miRNAs, 29 miRNAs were found to be significantly up regulated in the lesional epidermis compared with the non-lesional epidermis (Table 1). Figure 1 shows a representative plot of expression of miR-185, miR-202, miR-423, and miR-525 in lesional and non-lesional epidermis of vitiligo patients. As is clear from the figure, normalized MFIs were higher in the lesional skin (shown as V) compared with the non-lesional skin (shown as N).

**Table 1:** List of micro RNAs (miRs) altered in lesional skin of vitiligo patients compared with non-lesional skin. miRNAs showed in bold were used for further analysis.

miRNA	p Value (Medians)	p Value (Means)	No. of samples showing upregulation (n = 18)
**miR**-**423**	**0**.**0008**	**0**.**0001**	**17/18**
**miR**-**185**	**0**.**0003**	**0**.**0058**	**14/18**
**miR**-**326**	**0**.**0031**	**0**.**0222**	**14/18**
**miR**-**202**	**0**.**0007**	**0**.**004**	**13/18**
**miR**-518 C*	**0**.**0009**	**0**.**0087**	**13/18**
**miR**-**518b**	**0**.**0049**	**0**.**0099**	**13/18**
miR-324-5p	0.0015	0.0012	13/18
**miR**-**525**	**0**.**0085**	**0**.**0047**	**12/18**
miR-373*	0.0004	0.0022	12/18
miR-498	0.0103	0.0023	12/18
miR-302c*	0.0024	0.0047	12/18
miR-129	0.0017	0.0052	12/18
miR-198	0.0313	0.0059	12/18
miR-527	0.0049	0.0135	12/18
miR-320	0.009	0.0046	11/18
miR-526c	0.0012	0.0057	11/18
miR-450	0.0039	0.009	11/18
miR-503	0.004	0.0105	11/18
miR-let-7e	0.0012	0.0158	11/18
miR-383	0.0391	0.0236	11/18
miR 516-5p	0.0026	0.0074	10/18
miR-335	0.0098	0.013	10/18
miR-206	0.0151	0.0179	10/18
miR-519e	0.0156	0.0221	09/18
miR-526a	0.0494	0.0326	09/18
**miR**-**518a**-2*	**0**.**0137**	**0**.**0134**	**08/18**
miR-184	0.0353	0.0243	08/18
miR-500	0.0342	0.0252	08/18
miR-452	0.0151	0.0329	08/18

### Identification of putative targets of microRNAs regulated in lesional skin of vitiligo using bioinformatics tools

Of the 29 miRNAs upregulated in the lesional skin (Table 1), we selected 8 miRNAs (based on the number of samples showing their upregulation in the lesional skin), miR-423, miR-185, miR-326, miR-202, miR-518a, miR-518b, miR-518c and miR-525, for identification of their targets using a bioinformatics tool, Target scan, an online software. miR-518a was showing upregulation in only 8 of the 18 samples, however, since miR-518 b and c were upregulated in more number of samples, we selected miR-518a as well. Each miRNA was predicted to have many targets, categorised as Keratins, Melanogenesis genes (*TYR*, *TYRP1*, *DCT*, and *MITF*), Oxidative stress genes (*PRDX6*, *PRDX3*, *TMX4*) and vesicular trafficking genes (*RAB25*, *RAB27a*, *VAMP1*, *VAMP2*). These targets have been implicated in pathogenesis of vitiligo.

### Quantitative proteomic analysis of miRNA induced regulation of proteins in keratinocytes

While the bioinformatics analysis does suggest the putative targets, we wanted to study the actual targets using proteomic tools to check the overlap between the prediction and actual regulation. Since the lesional skin lacks melanocytes and is rich in keratinocytes, we transfected normal human epidermal keratinocytes (NHEKs) with six pre-miRNAs, pre-miR-185, pre-miR-202, pre-miR-525-5p, pre-miR-326, pre-miR-518a-5p and pre-miR-518c and also fluorescent Dy547-labelled miRNA control to ascertain efficiency of transfection (Supplementary Fig. 1). We observed more than 90% transfection efficiency (visual estimation) in the scrambled control transfections suggesting that the same level of efficiency may have been achieved in the pre-miRNA transfected keratinocytes as well. RNA and proteins were harvested from control and transfected samples for further analysis.

Protein samples were subjected to relative quantitative proteomics using iTRAQ mass spectrometry. Analysis of the proteomics data showed several proteins to be perturbed (Table 2, Supplementary Tables 1 and 2). Interestingly, however, the numbers of proteins predicted by Target scan to be regulated and actually regulated were different with different miRNAs as shown in the Venn diagrams in Supplementary Figures. Number of proteins common between prediction and actually regulated were 6 by miR-326, 82 by miR-518a-5p (Supplementary Fig. 2), 60 by miR-518c, 61 by miR-185 (Supplementary Fig. 3), 25 by miR-202 and 50 by miR-525-5p (Supplementary Fig. 4). These data clearly show that there may be several putative targets based on seed sequence match, however, number of actual targets may be different.

**Table 2:** Number of proteins regulated i.e. upregulated or downregulated in primary keratinocytes transfected with specified miRNAs.

Regulation of proteins	miR 326	miR 518a	miR 518c	miR 185	miR 202	miR 525
Downregulated	299	308	266	234	206	225
Upregulated	263	313	296	301	254	267

**Figure 2:**
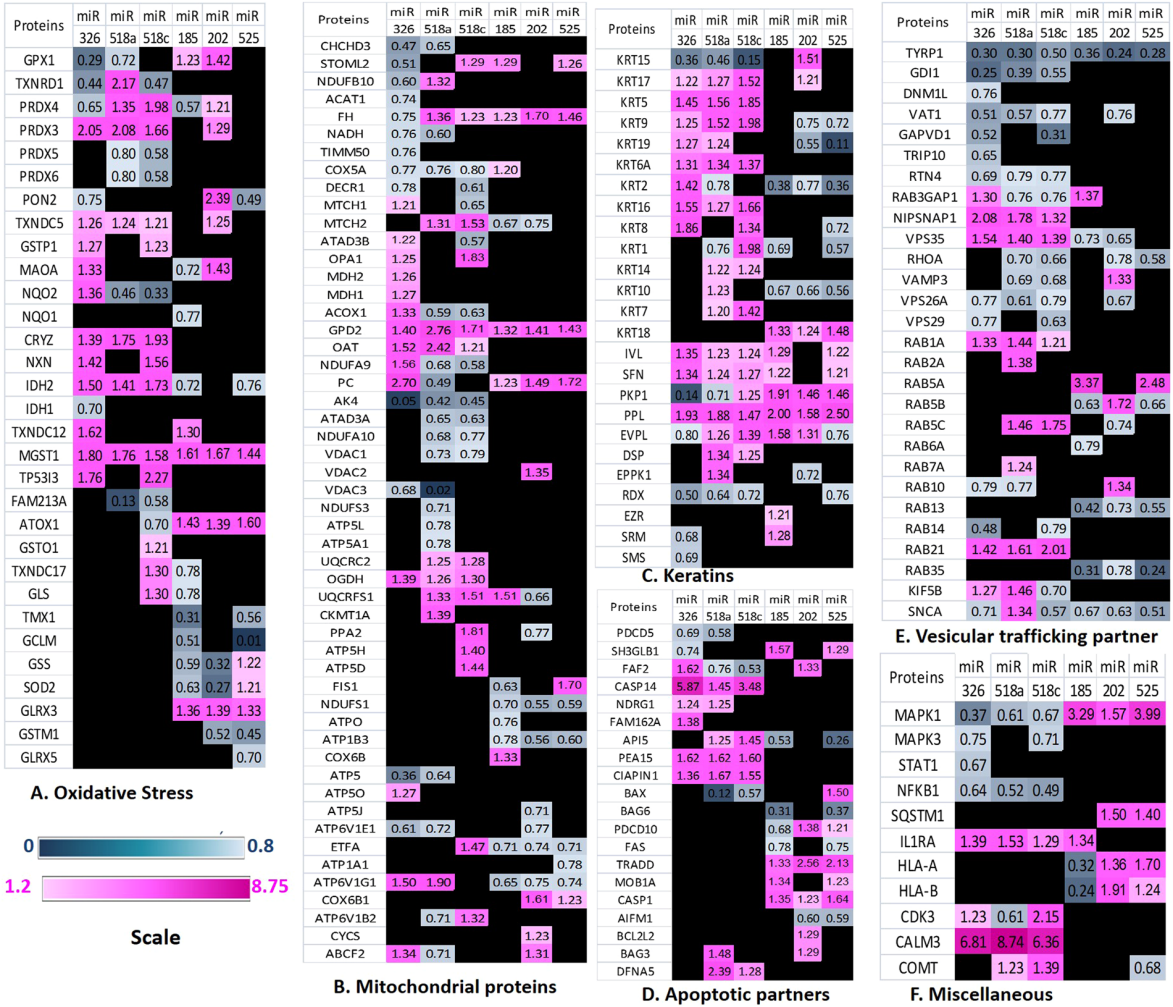
Heat Map of proteins regulated in keratinocytes after transfection with different miRNAs as revealed by iTRAQ mass spectrometry. Proteins have been grouped into different networks and their relative expressions at protein level are depicted where fold change of ≤0.8 is shown as downregulated in blue colour and fold change ≥1.2 is shown as upregulated in pink colour. (**A**) Oxidative stress Proteins; (**B**) Mitochondrial proteins; (**C**) Keratins & Structural proteins; (**D**) Apoptosis proteins; (**E**) Vesicular trafficking partners; (**F**) Miscellaneous protein including Immune System proteins & related Kinases.

Further, the proteins which were found to be regulated could be classified into different networks like, proteins involved in oxidative stress (Fig. 2A, Supplementary Fig. 5, PRDXs, TXN, SOD, GSS, GPX, MAO etc.), Mitochondrial proteins (Fig. 2 B and Supplementary Fig. 6, UQCRC2, ATP5O, MTCH2, NDUFS1, COX5A etc.), Keratins and structural proteins (Fig. 2C and Supplementary Fig. 7, KRT 1, KRT 2, KRT 9, KRT 14, KRT 15 and KRT 19 etc.), Cellular apoptosis (Fig. 2D and Supplementary Fig. 8, BAX, BCL2, CASP1, CASP14, FAF, FAS, AIFM1), Vesicular trafficking partners (Fig. 2E and Supplementary Fig. 9. RAB5B, RAB5C, RAB10, RAB14, RAB1A, RAB21, SEPT11, ARF3 etc.), and proteins and kinases involved in immune responses (Fig. 2F and Supplementary Fig. 10, NFKB1, STAT1, HLA-A, HLA-B etc.).

**Figure 3:**
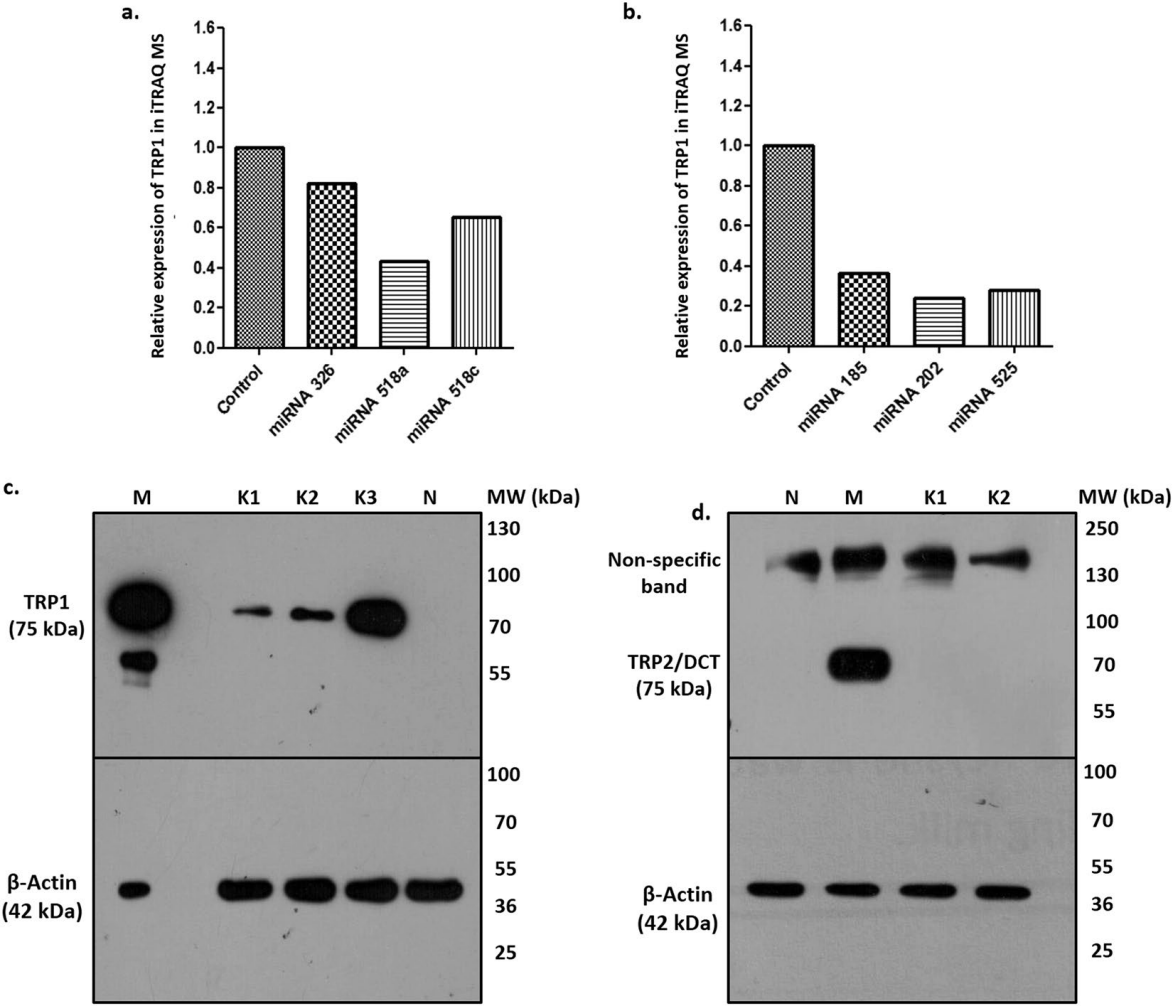
Expression of Tyrosinase related protein 1 (TRP1) in keratinocytes: (**a**,**b**) show relative expression of TRP1 protein in six miRNA transfected Keratinocytes compared with control as revealed by iTRAQ Mass Spectrometry. (**c**) Expression of TRP1 at protein level in Keratinocyte cell lysates in K1, K2 and K3 samples shows variable expression. M is Melanocyte lysate used as positive control where TRP1 expression is very abundant, N is NIH3T3, negative control, where there is no expression of TRP1.Full gel pictures of this cropped gel are shown in Supplementary Fig. 11. The lower Immunoblot represents β-Actin as loading control. (**d**) Expression of TRP2/DCT in NIH3T3 (N), melanocytes (M) and two samples of keratinocyte cultures K1 and K2, to show the expression of the melanogenic protein only in melanocytes and not in keratinocytes, full gel pictures are shown in Supplementary Fig. 11.

Interestingly, Tyrosinase related protein 1 (TRP1/TYRP1), a protein involved in melanogenesis, was found to be consistently downregulated in keratinocytes after transfection with all the pre-miRNAs tested, as revealed by mass spectrometry (Fig. 3a,b). TRP1 is a member of Cu^++^/Zn^++^ metallo-enzymes, which also include Tyrosinase (TYR) and TRP2 (DCT)^21^. Cu^++^/Zn^++^ metallo-enzymes are expressed in Melanocytes and control the process of melanogenesis wherein TRP1 acts as a weak DHICA (Dihydroxy indole carboxylic acid) oxidase^22^. Expression of all three enzymes i.e. TYR, TRP1 and TRP2 is regulated by Microphthalmia-Associated Transcription Factor (MITF), however, TRP1 expression may also be regulated independently^23^. TRP1 forms a heterodimer with Tyrosinase (TYR) and negatively regulates its activity to attenuate tyrosinase mediated toxicity^24^.

### Expression of TRP1 in keratinocytes

It was intriguing to find TRP1, a melanogenesis protein, to be down regulated in miRNA transfected NHEKs since keratinocytes have not been reported to express TRP1. So, we first confirmed that keratinocytes do actually express TRP1, (Fig. 3c,d and Supplementary Fig. 11), however, variable expression of TRP1 was observed in normal human keratinocytes. To confirm that the expression in TRP1 in keratinocytes was not coming from any contaminating melanocytes in the culture, we probed the western blots with another melanogenesis protein Tyrosinase related protein 2 (TRP2, also known as DCT). As is clear from Fig. 3d (Supplementary Fig. 11), none of the keratinocyte lysates stained for TRP2, while melanocyte lysates did show a positive staining, confirming that our keratinocytes were devoid of any melanocyte contamination and that TRP1 was indeed expressed on keratinocytes.

Since its role in keratinocyte has not been elucidated, we speculated that TRP1 might play a role in melanosome uptake or melanosome maturation in Keratinocytes and might contribute to vitiligo pathogenesis.

### Downregulation of TRP1 by selected miRNA

**Figure 4:**
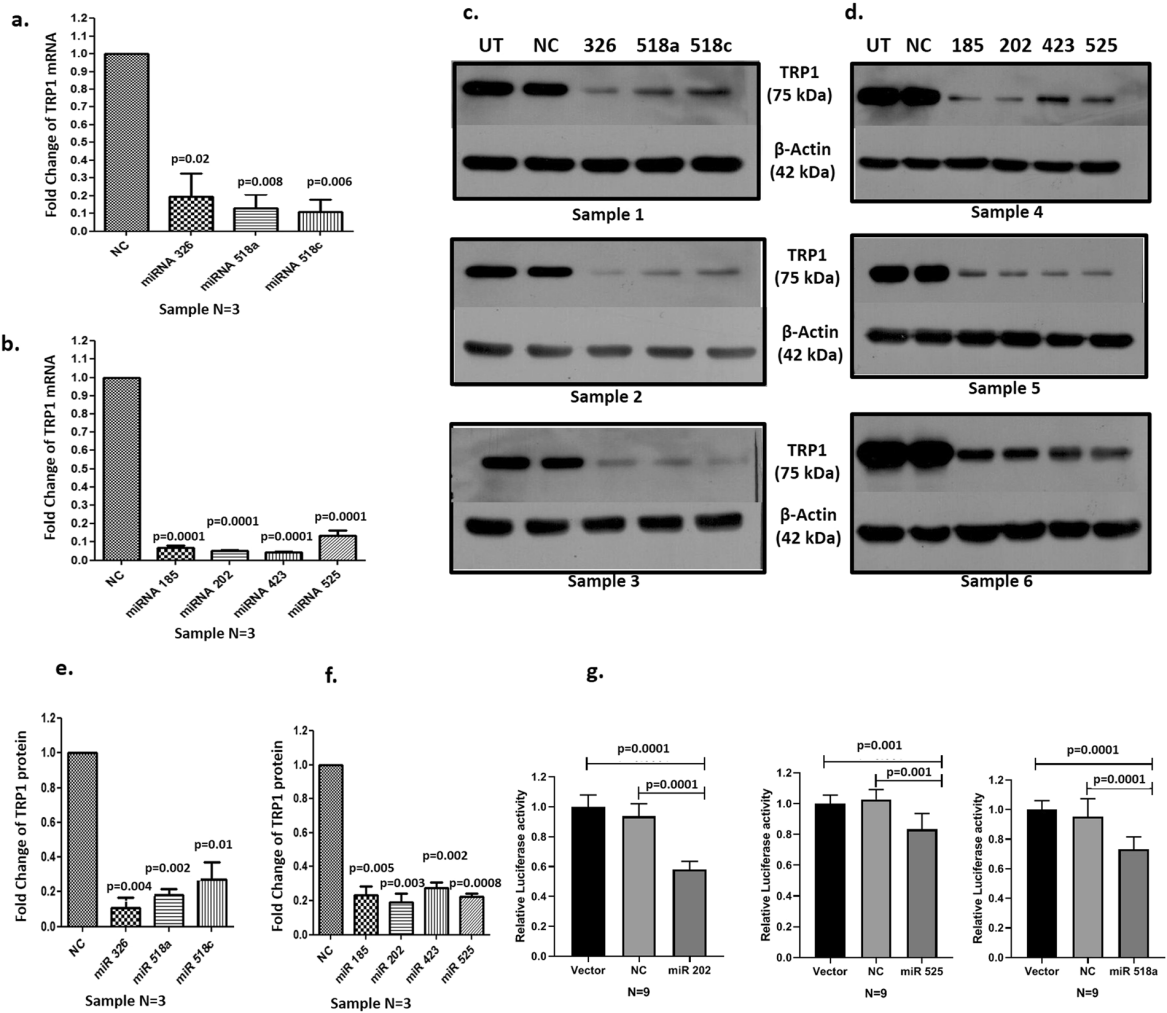
Downregulation of Tyrosinase related protein 1 (TRP1) by selected miRNA at mRNA and protein levels. (**a**) TRP1 mRNA profiling was done by qPCR and normalized with NC (negative control i.e., scrambled control). Expression of TRP1 in keratinocytes at mRNA level after transfection with pre-miR-326, pre-miR-518a-5p and pre-miR-518c, (**b**) Transcriptional expression of TRP1 in keratinocytes after transfection with pre-miR-185, pre-miR-202-3p, pre-miR-423-3p and pre-miR-525-5p (**c**) Western blot of TRP1 expression after transfection with pre-miR-326, pre-miR-518a-5p, pre-miR-518c. UT is Untransfected control. Full gel pictures are shown in Supplementary Fig. 12. (**d**) Western blot of TRP1 expression after transfection with pre-miR-185, pre-miR-202-3p, pre-miR-423-3p and pre-miR-525-5p. Full gel pictures are shown in Supplementary Fig. 13. (**e**) Densitometric analysis of TRP1 protein expression in pre-miR-326, pre-miR-518a-5p, pre-miR-518c transfected keratinocytes after normalization with β- Actin, (**f**) Densitometric analysis of TRP1 protein expression in pre-miR-185, pre-miR-202-3p, pre-miR-423-3p and pre-miR-525-5p transfected keratinocytes after normalization with β-Actin (N = 3), (**g**) Target validation assay results showing significantly reduced relative luciferase activity of 3'UTR clones transfected with pre-miR-202-3p, pre-miR-518a-5p and pre-miR-525-5p. All the bars showing reduced expression are depicted as mean ± standard error of mean (S.E.M).

To validate the iTRAQ Mass spectrometry data with respect to TRP1 expression, we transfected normal human epidermal keratinocytes (NHEK) with selected pre-miRNAs, i.e., pre-miRNA-185, pre-miR-202, pre-miR-423-3p, pre-miR-525-5p, pre-miR-326, pre-miR-518a-5p and pre-miRNA-518c and expression of TRP1 was checked at both mRNA and protein levels using quantitative PCR (qPCR) and western blots respectively. TRP1 was found to be significantly downregulated after miRNA transfection at both mRNA (Fig. 4a,b) and protein levels (Fig. 4c–f, Supplementary Figs 1^2,13^).

### TRP1 profiling in lesional and non-lesional epidermal samples from vitiligo patients

**Figure 5:**
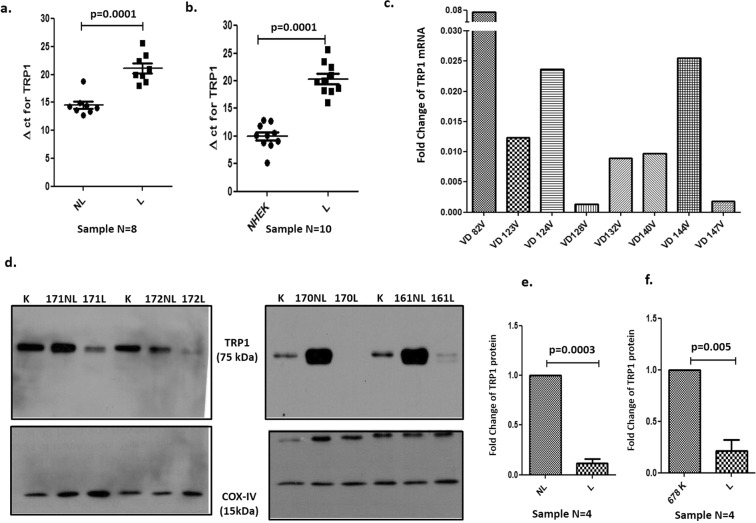
Expression of TRP1 in lesional (L), non-lesional (NL) epidermis and Normal human epidermal keratinocytes (NHEK). (**a**) Transcriptional expression of TRP1 shown as ΔCT in non-lesional and lesional epidermis from vitiligo patients. (n = 8) (**b**) Transcriptional expression of TRP1 shown as ΔCT in NHEK (n = 10) and lesional epidermis (n = 10) from vitiligo patients, (**c**) Downregulation of TRP1 expression in terms of fold change in lesional epidermis compared with non-lesional epidermis in 8 Vitiligo patients samples (VD). (**d**) Western blot for expression of TRP1 in four samples of non-lesional vs. lesional epidermis and normal human epidermal keratinocytes (678 K) at protein level showing lower expression in lesional epidermis. Full gel pictures are shown in Supplementary Fig. 14. (**e**) Densitometric analysis for expression of TRP1 in Non-lesional vs. Lesional epidermis normalized with endogenous control COX-IV. (**f**) Densitometric analysis for expression of TRP1 in Keratinocytes (678 K) vs. Lesional epidermis. All the bars showing reduced expression are depicted as mean ± S.E.M.

We further checked the expression of TRP1 in mRNA and protein samples from lesional and non-lesional epidermis of vitiligo patients. TRP1 was found to be significantly downregulated in lesional epidermis as compared with non-lesional epidermis and NHEKs at both mRNA (Fig. 5a–c) and protein levels (Fig. 5d–f Supplementary Fig. 14). The TRP1 protein input was normalized with endogenous control COX-IV. Expression of TRP1 in terms of fold change was significantly lower in the lesional epidermis when compared with non-lesional epidermis (Fig. 5e) and NHEK (Fig. 5f). We did not use beta actin as loading control for lesional and non-lesional skin as beta actin showed regulation in the micro-array analysis done earlier in the lab^20^. Hence COX-IV was used as the loading control. It was interesting to note that seventeen of the 18 patients initially studied for profiling micro RNAs showed upregulation of one or more of the selected miRNAs (i.e., miRNA-185, miRNA-202, miRNA-423, miRNA-525, miRNA-326, miRNA-518a and miRNA-518c), that induced down regulation of TRP1 in keratinocytes (Supplementary Fig. 15).

### Bioinformatics prediction of miRNA binding site on TRP1 gene

Since all the miRNAs studied i.e., miR-185, miR-202, miR-423-3p, miR-525-5p, miR-326, miR-518a-5p and miR-518c induced consistent downregulation of *TRP1*, we employed Target Scan algorithm to find the seed sequences in the *TRP*1 3′UTR that would have miRNA binding sites. Interestingly, this analysis revealed that only 3 micro RNAs, miR-525-5p, miR-518a-5p and miR-202 had seed sequences that may bind to TRP1 mRNA at 3′ UTR (Supplementary Fig. 16). However, other miRNAs did not show any targeting sequences, suggesting that downregulation of TRP1 after transfection with these miRNAs may be due to regulation of some other genes that may be the targets of these miRNAs, which in turn may be regulating TRP1.

To check whether the target sites of miR-202-3p, miR-525-5p and miR518a-5p are conserved in other species, we again used the online software Target scan. According to Target Scan prediction, target sequences of all three miRNAs seem to be poorly conserved where miR-202-3p had conserved target sequences only in Chimpanzee (Supplementary Fig. 17), target sequences of miR525-5p and miR518a-5p showed conserved sequences in both Rhesus and Chimpanzee (Supplementary Figs 18, 19).

### Target validation assay

To validate the targets of miR-202-3p, miR-518a-5p and miR- 525-5p, the regions of *TRP1* 3′UTR encompassing their targets were PCR amplified (for miR-202-3p the amplicon size was 447 base pairs (bp), for miR-518a-5p: 507 bp and for miR-525-5p: 507 bp) and cloned into pmir-GLO dual luciferase vector with *SacI* and *XhoI* restriction sites. Subsequently, different chimeric constructs containing the target 3′UTR regions of *TRP1* were co-transfected along with each one of their corresponding pre-miRNAs into HeLa cells. Firefly luciferase reporter activity was acquired 48 hours after co-transfection. Negative control consisted of a nonspecific miRNA mimic, co-transfected with each chimeric construct into HeLa cells. Transfection of pre-miR-202, pre-miR-518a-5p and pre-miR-525-5p (40 nM) significantly reduced luciferase activity of *TRP1* gene (Fig. 4g). Pre-miR-202 reduced luciferase activity (relative expression 0.58 ± 0.02) by 41.94% compared to Vector and by 38.10% compared to Negative scramble control (NC). Pre-miR-518a-5p reduced luciferase activity (relative expression 0.73 ± 0.03) by 26.77% compared to Vector and by 23.26% compared to NC. Pre-miR-525-5p reduced luciferase activity (relative expression 0.83 ± 0.03) by 16.65% compared to vector and by 18.87% compared to NC. (Fig. 4g). These data clearly suggest that these three miRNAs bind to their specific target miR-recognition sites present in the 3′UTR of human *TRP1* resulting in downregulation of *TRP1* expression.

### Probable role of TRP1 in keratinocytes

**Figure 6:**
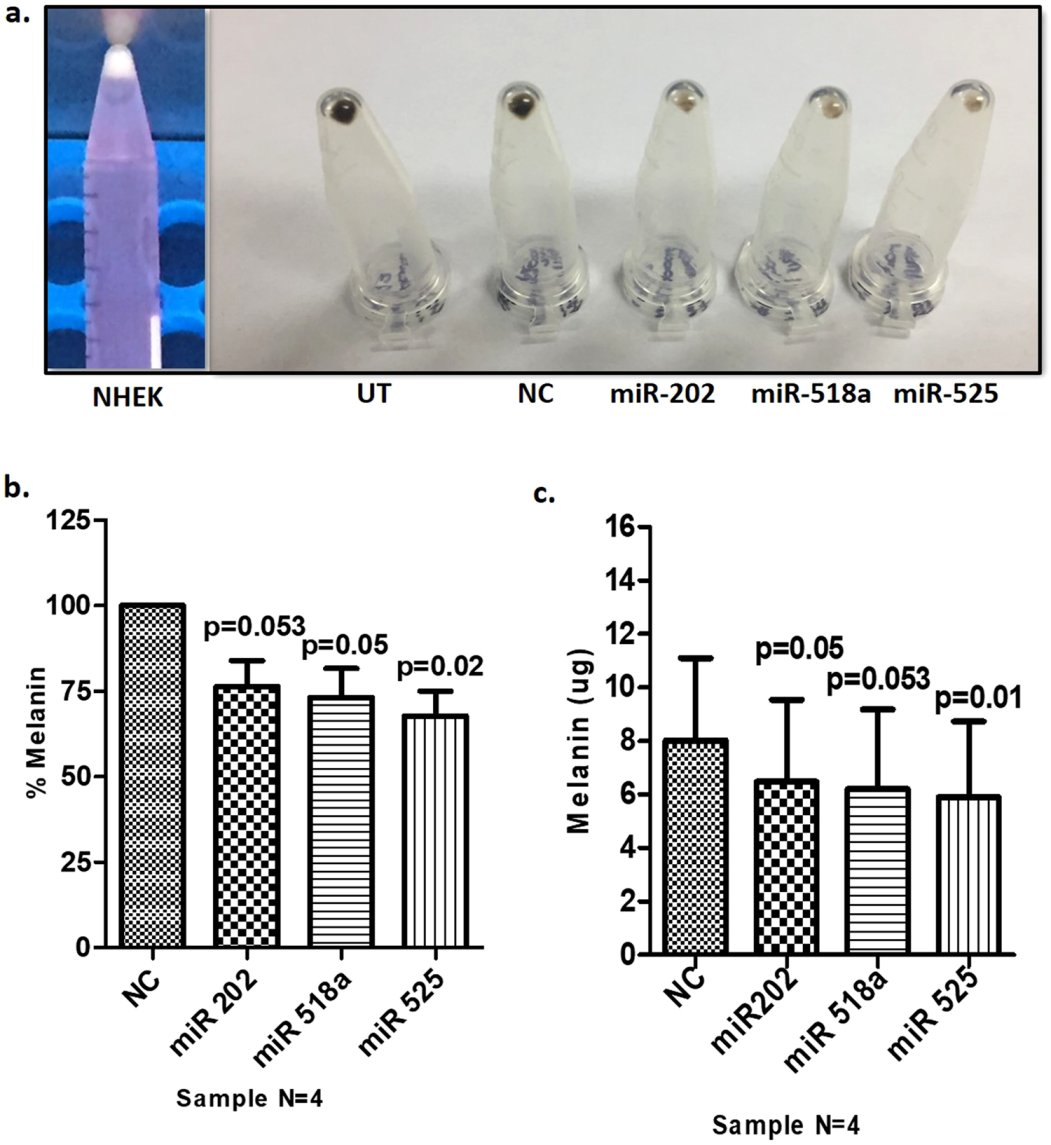
Melanosome transfer from melanocytes to keratinocytes after transfection of keratinocytes with selected pre-miRs. (**a**) Representative image for pellet color in pure Keratinocyte culture (NHEK, with no pigment) and keratinocytes transfected with miRNAs, co-cultured with melanocytes and differentially trypsinized to get only pure keratinocytes. Pellet color is lighter in transfected Keratinocytes co-cultured with Melanocytes as compared to scrambled control (NC). UT is Untransfected control. (**b**) Bar graph showing percentage decrease in Melanin content in keratinocytes that were transfected with, pre-miR-202, pre-miR-518a-5p and pre-miR-525-5p, co-cultured with melanocytes and obtained 48 hours post co-culture by differential trypsinization. As compared to the scrambled  control i.e. NC (100%, 8.03 ± 3.1 µg), miR-202 showed 76.32 ± 7.6% (6.48 ± 3.1 µg), miR-518a showed 73.2 ± 8.4% (6.21 ± 2.98 µg) and miR-525 showed 67.75 ± 7.3% (5.89 ± 2.8 µg) melanin content (**c**) Bar graph showing absolute melanin content in the keratinocyte cultures mentioned in (**b**) All the bars showing reduced expression are depicted as mean ± S.E.M mentioned above.

Down regulation of TRP1 in the NHEKs by the miRNAs, that were upregulated in lesional epidermis of vitiligo patients, led us to question the probable implication of this miRNA mediated downregulation of TRP1 in pathophysiology of vitiligo. To address this question, we cultured keratinocytes and transfected them with three pre-miRNAs for miR-202, miR-518a-5p and miR-525-5p, which were predicted and validated to target TRP1. These transfected keratinocytes were then co-cultured with primary epidermal melanocytes for 48 hours and thereafter keratinocytes and melanocytes were harvested by differential trypsinization. As is clear from Fig. 6a, pure naïve normal human epidermal keratinocytes (NHEKs) in culture don’t have any pigment and look absolutely white in pellet. However, keratinocytes co-cultured with melanocytes acquired melanosomes/melanocores from the melanocytes which is clear from the pellet colour of the keratinocytes harvested after differential trypsinization and Melanin estimation. Both, pellet colour visualization and melanin content revealed that miR-transfected keratinocytes, when co-cultured with melanocytes, showed significantly less amount of melanin content (Fig. 6a–c). As compared to the scrambled control (100%, 8.03 ± 3.1 µg melanin), miR-202 showed 76.32 ± 7.6% (6.48 ± 3.1 µg melanin), miR-518a showed 73.2 ± 8.4% (6.21 ± 2.98 µg melanin) and miR-525 showed 67.75 ± 7.3% (5.89 ± 2.8 µg) melanin content. These data suggest that miRNA mediated downregulation of TRP1 hampers either melanosome transfer to the keratinocytes or melanosome maturation in the keratinocytes resulting in reduced melanin content.

### Transfection of keratinocytes with TRP1 siRNA and co-culture with melanocytes

**Figure 7:**
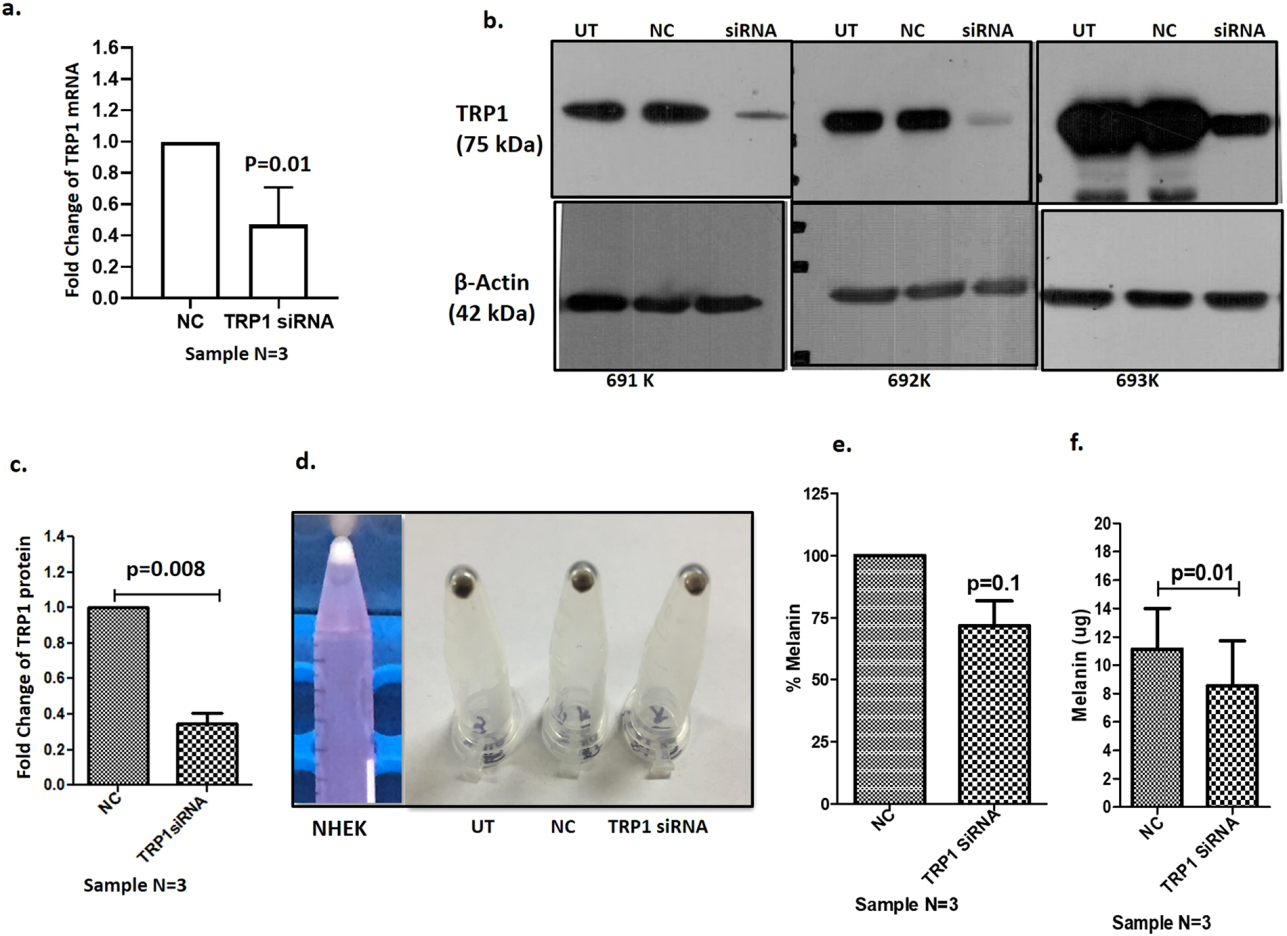
Melanosome transfer from melanocytes to TRP1 siRNA transfected keratinocytes. (**a**) Transcriptional expression of TRP1 in Normal human epidermal keratinocytes (NHEKs) transfected with TRP1 siRNA in 3 samples compared with scrambled control (NC). (**b**) Western blots for expression of TRP1 protein in the same 3 NHEK samples transfected with TRP1 siRNA (full gel pictures are shown in Supplementary Fig. 20. (**c**) Densitometric plots for TRP1 expression as compared with NC normalized with endogenous control β-Actin, confirm downregulation of TRP1 in siRNA transfected keratinocytes. (**d**) Representative image for pellet color in pure keratinocytes, TRP1 siRNA transfected keratinocytes that were co-cultured with melanocytes and harvested by differential trypsinization. Pellet color of keratinocytes transfected with siRNA and co-cultured with melanocytes, is lighter as compared to the keratinocytes transfected with scrambled control (NC) co-cultured with melanocytes. (**e**,**f**) Bar graphs showing percentage decrease in melanin and total melanin content in keratinocytes transfected with siRNA compared with that of keratinocytes transfected with scrambled control after co-culture with melanocytes. The melanin content in TRP1 siRNA transfected keratinocytes co-cultured with melanocytes was reduced from 100% (11.13 ± 2.87 µg) in NC to 71.68 ± 10.06% (8.53 ± 3.19 µg). While there is about 29% decrease, the percent decrease does not show statistically significant difference since for all the samples the reduction was calculated from 100%. However, absolute melanin content clearly shows significant reduction in melanin content in siRNA transfected keratinocytes.

Since miRNA mediated downregulation of TRP1 resulted in reduced melanin content in miRNA transfected keratinocytes after co-culture with melanocytes due to impaired melanosome/melanocore transfer and/or melanosome maturation in the keratinocytes, we wanted to validate whether this effect indeed was due to TRP1 downregulation by miRNAs. miRNAs have several targets and to validate that the impaired melanosome /melanocore transfer was actually happening through downregulation of TRP1 only, we transfected epidermal keratinocytes with TRP1 siRNA, validated its downregulation at mRNA (Fig. 7a) and protein levels (Fig. 7bc, Supplementary Fig. 20) in 3 keratinocyte cultures.

After transfection with TRP1 siRNA for 48 hours, NHEKs were co-cultured with primary epidermal melanocytes for 48 hours and thereafter keratinocytes and melanocytes were harvested by differential trypsinization. The melanin content in TRP1 siRNA transfected keratinocytes co-cultured with melanocytes was reduced from 100% (11.13 ± 2.87 µg) in negative control samples to 71.68 ± 10.06% (8.53 ± 3.19 µg) (Fig. 7e,f), suggesting that downregulation of TRP1 in keratinocytes resulted in impaired melanosome/melanocore transport or melanosome maturation in keratinocytes.

## Discussion

We report here the proteomic landscape of altered protein expression in keratinocytes induced by miRNAs that were upregulated in the lesional skin of vitiligo patients. We have studied the targets of the most significantly regulated miRNAs using a bioinformatics tool Target Scan and a proteomic tool i.e., iTRAQ mass spectrometry and further tried to elucidate the role of micro RNA induced down regulation of TRP1 in keratinocytes. Interestingly, while there were some targets common between the Target scan prediction and proteomic analysis, there were some unique targets identified by the two approaches. These differences in the two approaches are quite understandable since the target scan would predict any genes having the seed sequence match in the 3′or 5′UTR for a particular micro RNA. On the other hand, the proteomic analysis shows the actual regulation of proteins after miRNA transfection, where the targets could be both direct as well as indirect. Genes interact in integrated networks and functions and expressions of one gene may be modulated or altered by another, which is captured in the proteomic analysis and may be missed by the bioinformatics tools.

Since the identified miRNAs were upregulated in the lesional skin, which mainly comprises of keratinocytes, we transfected six miRNAs in the keratinocytes and studied the altered protein expression by Quantitative proteomics using iTRAQ mass spectrometry. Analysis revealed several proteins to be perturbed. An integrated effect of six micro RNAs i.e., miR-185, miR-202-3p, miR-525-5p, miR-326, miR-518a-5p and miR-518c perturbed several proteins involved in oxidative stress, Mitochondrial proteins, Keratins, Cellular apoptosis, Vesicle trafficking, and proteins involved in immune responses, all of which may be contributing to vitiligo pathogenesis in an integrated manner. The RABs found to be perturbed in proteomics data have not been characterized in vitiligo but their function is known in vesicular trafficking. One of the RABs, RAB5b whose silencing has been shown to significantly impair melanocore uptake by keratinocytes^25^, was also downregulated in the present study by miR-185 and miR-525-5p, the two micro RNAs which were upregulated in 14 and 12 of the 18 vitiligo patients’ lesions respectively (Supplementary Fig. 15).

Besides these proteins, TRP1 was found to be downregulated in the keratinocytes after transfection with all the six micro RNAs studied. TRP1 is a melanogenesis protein expressed in melanocytes, that plays a crucial role in mammalian pigmentation and has been known as the classic *brown* locus in mice^26–28^. TRP1 has been implicated as an autoantigen in vitiligo due to presence of autoantibodies to TRP1 in sera of vitiligo patients^29^. Several studies have shown that mutations in *TRP1* are associated with brownish coat colour in cats^30^, dogs^31^, cattle^32^, sheep^33^ and pigs^34^. A mutation in exon 2 of TRP1 gene in rabbits results in premature stop codon at position 190 resulting in a truncated TRP1 protein that is associated with *brown* coat colour in rabbits^35^. Human TRP1 displays high levels of homology with bovine and mouse counterparts^36^. It has been shown to be associated with melanocyte differentiation and malignant melanomas^37^. TRP1 is expressed on both the cell surface and intracellularly in human and mouse melanocytes and melanomas and can thus be a target for antibodies. Long-term treatment or passive transfer of monoclonal antibody against TRP1 led to melanoma regression and vitiligo like patchy depigmentation in mice^38,39^.

The 3′UTR region of TRP1 gene consists of 7.6 kilobases (ENST00000388918.5), and harbours several putative miRNA binding sites. Of the seven miRNAs screened, miR-185, miR-202-3p, miR-423-3p, miR-525-5p, miR-326, miR-518a-5p and miR-518c, we found seed sequences that could bind only miR-202-3p, miR-525-5p and miR-518a-5p in 3′ÚTR of the *TRP1* gene. The binding sites for all three miRNA seem to be poorly conserved. miR-202-3p site seems to be conserved in Chimpanzee, while miR-525-5p and miR-518-5p sites are conserved in both Rhesus and Chimpanzee. Target validation assay confirmed that the sites in 3′UTR of TRP1 gene predicted to be targets of these miRNAs were indeed targeted by them. Pre-miRNAs are expected to get processed into mature miRNAs and it is highly likely that they get processed to both 3p and 5p species, which would be true *in*-*vivo* as well. However, the aim of the present study was not to find out whether it is 3p of 5p species that is getting processed and regulating the protein expression, but to investigate the proteomic perturbations induced after transfection with the pre-miRNAs in keratinocytes in order to understand their role in aetiopathogenesis of vitiligo. So, we transfected keratinocytes with pre-miRNAs in order to get them processed into miRNAs as they would do *in*-*vivo*. Our results did show downregulation of TRP1 by three miRNAs which were validated to have the target sequences in TRP1 3′UTR. So, we further tried to find the relevance of this down regulation in the context of vitiligo.

TRP1 is a melanogenesis protein and its reduced expression in keratinocytes after transfection was quite intriguing as its role in keratinocytes has not been elucidated so far. So, we first confirmed the expression of TRP1 in normal human epidermal keratinocytes and then validated its downregulation after transfection with the miRNAs and also its expression in the lesional and non-lesional epidermis. We found that indeed TRP1 was expressed on the keratinocytes and was downregulated after transfection by all the micro RNAs studied (Fig. 4). The lesional skin, which shows higher expression of one or more selected micro RNAs (Supplementary Fig. 15), also showed significant downregulation of TRP1 (Fig. 5). One may argue that since the lesional skin lacks melanocytes, the melanogenesis gene *TRP1* would obviously be down regulated in the lesional skin. However, this downregulation of both the gene as well as protein was significant when compared to normal human keratinocytes as well (Fig. 5b,d,f), suggesting that TRP1 does have a definite role to play in keratinocytes which is defective in the lesional skin and this down regulation is miRNA (upregulated in the lesional skin) induced and not just due to the absence of melanocytes.

It is well established that melanocytes and keratinocyte live in a symbiotic relationship in the epidermis where melanocytes synthesize and transfer melanosomes to the surrounding keratinocytes to save them from the UV induced damage^17,18^. Keratinocytes, on the other hand, produce growth factors for melanocytes to proliferate and for melanogenesis^19^. Melanosomes are transported from the perinuclear space of melanocytes to their dendritic tips from where they are transported to surrounding keratinocytes^17^. A defect in melanosome transfer could be detrimental for the survival of both melanocytes and keratinocytes. So, we hypothesized that down regulation of TRP1 by these micro RNAs may have a role in melanosome transfer from melanocytes to keratinocytes or melanosome maturation in the keratinocytes. To validate the hypothesis we selected three micro RNAs, pre-miR-202-3p, pre-mirR-518a-5p and pre-miR-525-5p that were predicted and validated to have seed sequences in TRP1 3′UTR by Target scan software and target validation assay respectively. Co-cultures of keratinocytes (transfected with these three pre-miRs) with melanocytes resulted in reduced melanin content in the keratinocytes suggesting that this could be either due to reduced melanosome transfer or melanosome maturation, if stage II melanosomes were transferred which would mature in keratinocytes. However, this could not be elucidated whether the reduction in melanin content was due to reduced melanosome transfer or maturation.

Micro RNAs have several targets. To confirm that reduced melanin content in the keratinocytes was due to TRP1 downregulation only, and not by any other protein regulated by selected miRNAs, we used siRNA approach. Downregulation of TRP1 by siRNA in primary keratinocytes and co-culture with melanocytes also significantly reduced melanin content in keratinocytes, confirming the miRNA results that downregulation of TRP1 in keratinocytes hampers either melanosome transfer or maturation. Along with TRP1, we also observed downregulation of an early endosomal protein RAB5b by miR-185 and miR-525-5p. Silencing of RAB5b has also been shown to impair melanocore uptake by keratinocytes^25^. Seven of the 29 miRNAs that were studied, i.e. miR-326, miR-518a-5p, miR-518c, miR-185, miR- 202-3p, miR-423-3p and miR-525-5p induced down regulation of TRP1 (Fig. 4, Table 1). A combined analysis shows that seventeen of the 18 patients showed upregulation of one or more of these miRNAs in the lesional skin compared to the non-lesional skin (Supplementary Fig. 15), suggesting their role in downregulating TRP1 individually or in concert with the other miRNAs in a synergistic manner.

Two of the upregulated miRNAs, miR-326 and miR-185, have been shown to be upregulated in keratinocytes exposed to 30 or 60 mJ ⁄ cm2 of UVB after 24 hours of irradiation^40^. Since Vitiligo patients are exposed to ultra violet rays, it is difficult to say whether this upregulation in the lesional skin is a consequence of the irradiation or is an inherent characteristic of the lesional skin.

Thus, our data suggest that some miRNAs found to be upregulated in lesional epidermis caused downregulation of TRP1 and RAB5b in Keratinocytes which negatively affect melanosome/melanocore transfer or melanosome maturation in the keratinocytes resulting in probable retention of melanosomes in melanocytes leading to melanin induced cytotoxicity and may contribute to the loss of melanocytes in the lesional skin. Further, it seems that early melanosomes are transferred to keratinocytes and they mature there in the presence of melanosomal proteins like TRP1 and RAB5b, whose downregulation by miRNAs may be detrimental for both melanocytes and keratinocytes. Recently, Tarafder *et al*.^41^ analyzed the fate of pigment within the keratinocyte. They demonstrated that exocytosed polymerised melanin alone, i.e. melanocore are surrounded by a membrane that lacks TRP1, a protein that is generally present on the melanosomal membrane within melanocytes^42^. Presence of TRP1 in keratinocytes, as observed in the current study, may thus be required for maintenance of melanosome integrity within the keratinocytes after uptake of melanocores.

In conclusion, we unequivocally show that micro RNAs have a role in aetiopathogenesis of vitiligo as they regulate several key proteins involved in oxidative stress, melanosome transfer, keratinization, apoptosis and immune responses. Most importantly, regulation of TRP1 by miRNAs in keratinocytes seems to have a role in melanosome transfer, melanosome maturation or in maintaining melanosome integrity in keratinocytes. Further studies are required to clearly elucidate the mechanisms by which TRP1 helps in maintaining melanosomes in keratinocytes. It is not the effect of one or two micro RNAs, but several micro RNAs exert their effects in an integrated manner and orchestrate their effects through different networks which precipitate in the depigmenting disorder vitiligo, suggesting that inhibition of the upregulated miRNAs may have therapeutic implications which need to be further explored.

## Methods

### Vitiligo patient skin biopsies

Skin punch biopsies (3 mm to 4 mm) from non-lesional and lesional regions were obtained from North Indian subjects (N = 18) undergoing punch grafting/melanocyte transplants^43^ at Dr. Ram Manohar Lohia Hospital (RMLH), New Delhi, after obtaining informed consent. The Human ethical review committee of RMLH and the National Institute of Immunology (NII), New Delhi, approved this study and is in agreement with the Declaration of Helsinki Principles. Normal Human epidermal melanocyte and keratinocyte cultures were kind gift from Dr. T. N. Vivek, Institute of Genomics and Integrative Biology, New Delhi, who had purchased them from M/s Lonza (India). Epidermal RNA was isolated by standard protocol using Tri Reagent (Sigma-Aldrich, USA) and then purified by rDNAse treatment. RNAs from melanocytes and keratinocytes cultures were isolated using NucleoSpin TriPrep kit (MACHEREY-NAGEL, Germany) following the manufacturer’s standard protocols. Highly pure RNA was then eluted with Nuclease free water. RNA was quantified in NANODROP 2000 Spectrophotometer and the RNA integrity was evaluated by running it on 1% agarose gel (for details see Supplementary Methods).

### FlexmiR microRNA assay and analysis

FlexmiR MicroRNA Human Panel and FlexmiR MicroRNA labeling Kit (Luminex Corp., USA) were used to assay miRNA expression pattern across paired non-lesional and lesional epidermis following manufacturer’s instructions. First RNA was biotin labeled, then hybridized to capture microspheres, before probing with streptavidin-PE and signal was acquired on the Luminex IS 100. Data were analysed using FlexmiR data analysis tool A02.29. The background signal was subtracted, quality control beads were verified, and normalized using 5 normalization microspheres for various sno-RNAs. Fold change was calculated for each sample pair (Supplementary Methods for details).

### Bioinformatics analysis

The target prediction analysis was done to identify putative mRNA targets for the miRNAs of interest. This was accomplished using online software, Target Scan (http://www.targetscan.org).

### Transfection of the primary cells with miRNA

Primary Keratinocytes were transfected with pre-miRNAs, pre-miRNA-185, pre-miRNA-202, pre-miRNA-525-5p, pre-miRNA-326, pre-miRNA-423-3p, pre-miRNA-518a-5p and pre-miRNA-518c and also with fluorescent Dy547-labelled miRNA control (miRIDIAN microRNA Mimic Transfection Control, CP-004500-01-05 5 nmol, microRNA mimic based on the C. elegans miRNA cel-miR-67) to ascertain efficiency of transfection in six well plates as described in Supplementary Methods. Mature miRNA and stem loop sequences of the pre-miRNAs are shown in Table 3. Briefly, reaction Mix A was prepared by diluting 5 μl of SiPort amine (Gibco, USA) in 100 μl of OptiMem (Gibco, USA). 6 μl (6.25 μM) of pre-miRNA and negative control (Ambion, life technologies, USA) were diluted with 100 μl of OptiMem to give reaction B. Reaction A and B were mixed and incubated at RT for 10 min and added to respective wells. Untransfected wells contained only OptiMem. Thereafter, 2 ml of cell suspension (0.20 × 10^6^ cells/well) was added to each well and incubated for 48 hrs. Transfection efficiency was monitored using the fluorescent-labelled control in the RFP filter channel of fluorescent microscope (Olympus). After incubation, the cells were harvested for RNA and protein to assess the expression of different genes. They were further co-cultured with Melanocytes for 48 hours and thereafter Keratinocytes were harvested by differential trypsinization to assess Melanosome transfer using melanin content estimation.

**Table 3:** Sequences of pre-miRNAs transfected in the keratinocytes (Sequences provided in the package inserts of Ambion products).

Name of the pre-miR	Mature miRNA sequence	Stem loop sequence
hsa-miR-202 (PM12718 Ambion)	AGAGGUAUAGGGCAUGGGAA	CGCCUCAGAGCCGCCCGCCG UUCCUUUUUCCUAUGCAUAU ACUUCUUUGAGGAUCUGGCC UAAAGAGGUAUAGGGCAUGG GAAAACGGGGCGGUCGGGUC CUCCCCAGCG
hsa-miR-326 (PM10686 Ambion)	CCUCUGGGCCCUUCCUCCAG	CUCAUCUGUCUGUUGGGCUG GAGGCAGGGCCUUUGUGAAG GCGGGUGGUGCUCAGAUCGC CUCUGGGCCCUUCCUCCAGC CCCGAGGCGGAUUCA
hsa-miR-518a-5p (PM12865 Ambion)	CUGCAAAGGGAAGCCCUUUC	UCUCAAGCUGUGACUGCAAA GGGAAGCCCUUUCUGUUGUC UAAAAGAAAAGAAAGUGCUU CCCUUUGGUGAAUUACGGUU UGAGA
hsa-miR-518c (PM10882 Ambion)	UCUCUGGAGGGAAGCACUUUCUG	GCGAGAAGAUCUCAUGCUGU GACUCUCUGGAGGGAAGCAC UUUCUGUUGUCUGAAAGAAA ACAAAGCGCUUCUCUUUAGA GUGUUACGGUUUGAGAAAAG C
hsa-miR-185 (PM12486 Ambion)	UGGAGAGAAAGGCAGUUCCUGA	AGGGGGCGAGGGAUUGGAGA GAAAGGCAGUUCCUGAUGGU CCCCUCCCCAGGGGCUGGCU UUCCUCUGGUCCUUCCCUCC CA
hsa-miR-423-3p (PM13123 Ambion)	AGCUCGGUCUGAGGCCCCUCAGU	AUAAAGGAAGUUAGGCUGAG GGGCAGAGAGCGAGACUUUU CUAUUUUCCAAAAGCUCGGU CUGAGGCCCCUCAGUCUUGC UUCCUAACCCGCGC
hsa-miR-525-5p (PM10581 Ambion)	CUCCAGAGGGAUGCACUUUCU	CUCAAGCUGUGACUCUCCAG AGGGAUGCACUUUCUCUUAU GUGAAAAAAAAGAAGGCGCU UCCCUUUAGAGCGUUACGGU UUGGG

### Quantitative proteomic analysis using iTRAQ labelling

Proteins were extracted from miRNA transfected keratinocytes using RIPA buffer (Thermo scientific, USA) supplemented with 1X MS-SAFE protease and phosphatase inhibitor (Sigma, USA) at 4 °C for 30 mins followed by centrifugation at 4 °C at 13000 RPM for 30 mins as described in Supplementary Methods. Supernatant was collected as protein lysate and stored at −80 °C.

Protein concentrations were estimated using Pierce BCA protein assay kit (Thermo Scientific, USA) as per manufacturer’s protocol and described in Supplementary Methods.

For iTRAQ labelling 150 µg of protein samples (Control and test sample) were taken, concentrated, trypsinized and labelled with iTRAQ labels as described in Supplementary Methods. All samples of an experiment labelled with different tags were pooled, short spun, vacuum dried and resuspended in 1 ml of 8 mM Ammonium formate buffer and subjected to strong cation exchange chromatography. Elution was done by passing a series of Ammonium formate buffers in 30% Acetonitrile (ACN). All fractions were vacuum dried and run for LC MS/MS operation through AB SCIEX 6600 QTOF System and data was analysed in AB SCIEX protein pilot software which provided whole proteomic perturbation in test samples vs. control. The data so obtained was analysed for proteomic perturbations i.e. for relative expression of a protein in miRNA transfected keratinocytes normalised to scrambled negative control (NC). Relative expression cut off ≤ 0.8 was considered as downregulation and relative expression cut off ≥ 1.2 was considered as upregulation of protein expression. Heat maps for the proteins regulated by different miRNA were created with Microsoft Excel 2007 software (Microsoft). (Please see Supplementary Methods for details).

### Network analysis of proteins perturbed by selected miRNAs as revealed by quantitative mass spectrometry

The proteins which were found to be regulated after transfection of pre-miRs, pre-miRNA-185, pre-miRNA-202-3p, pre-miRNA-525-5p, pre-miRNA-326, pre-miRNA-518a-5p and pre-miRNA-518c in normal human epidermal keratinocytes (NHEKs) were broadly categorised into different networks. Networks were prepared by collecting data regarding interacting partners of the regulated proteins derived from the String database (https://string-db.org/) and the relative expression of proteins derived from the proteomics experiments, in Cytoscape Software version 3.6.1 (Institute of System Biology, Seattle, WA, USA) which depicted the network of different pathways involved in aetiopathogenesis of vitiligo.

### Western blot and densitometry

The protein samples from various experiments were resolved on SDS PAGE gel in TGS buffer and then electro-blotted onto Polyvinylidene fluoride (PVDF) membrane (Millipore, USA). The blot was blocked with 5% NFDM (Non-fat dried milk)/TBST or 5% BSA/TBST for 1 hour at room temperature (RT) and probed for the proteins of interest by hybridizing with primary antibodies, secondary antibodies and loading control to detect corresponding proteins in various samples. The hybridized western blots were exposed to X-ray films for 1–2 seconds in developing cassettes in dark rooms under red light and X-ray films were developed and fixed using standard protocol. The X-ray films were scanned in HP LaserJet printer (536 dnf) and densitometry was done using Image J software. Statistical significance for the protein expression was calculated using student’s t-test in Graph Pad Prism 5.0 software (Supplementary Methods for details).

### Target validation assay for miRNA

The human *TRP1* (NM_000550.2) 3′ UTR fragments that were predicted to have binding sites for miR-202-3p, miR-518a-5p and miR-525-5p in Target Scan were PCR amplified from cDNA obtained from cultured melanocyte mRNA, using specific primers designed to have *SacI* and *XhoI* restriction sites (Sigma, India). Primers used were miR 202-3p- FP: 5′-GTGAGCTCCACTC TTAAATAACCATTGGGTC-3′, miR202-3p RP: 5′-GTCTCGAGAAAAGTTTCTTCTGATCCTTTTGTTTC-3′; miR518a-5p FP: 5′-GTGAGCTCGCTAAGTCCATGAATATCCAATAATG-3′, miR518a-5p- RP: 5′-GTCTCGAGTGGATAAAAC ATCCAGGC-3′; miR525-5p-FP: 5′-GTGAGCTCGTGAGCAAGGCTATGATAAAG-3′, miR525-5p RP: 5′-GTCTCGAGTTTTGGAGCATAAATAAAAAAGAATG C-3′. Negative control/scrambled control for target validation assay was MISSION®miRNA (HMC0003 Sigma) with Mature Sequence CGGUACGAUCGCGGCGGGAUAUC.

The amplified products (~500 bp) had *SacI* and *XhoI* restriction sites at terminal position, so they were digested with *SacI* and *XhoI* restriction enzymes and subsequently cloned downstream of the firefly luciferase coding sequence between *SacI* and *XhoI* sites of dual luciferase pmir-GLO reporter vector (Promega Life science, Madison, WI). To validate that miR-202-3p, miR-518a-5p and miR-525-5p target *TRP1* mRNA, the chimeric reporter constructs of pmir-GLO were co-transfected into HeLa cells along with 40 nM each of the test miRNA or scrambled control miRNA using Lipofectamine 3000 reagent (Invitrogen, Germany) as per manufacturer’s protocol. After 48 hours cells were lysed in 1X passive lysis buffer (Promega) and the lysates were used to measure Firefly and Renilla luciferase activity by Dual Luciferase reporter assay kit (Promega) as per manufacture’s protocol. The luminescence was measured in Orion 2 microplate Luminometer (Berthold detection systems, Germany). Renilla luciferase activity was used as control reporter for normalization. Results were expressed as relative luciferase activity compared to scrambled miRNA control transfected cells and vector only transfected cells. Target validation assay was done three times with three transfections for each miRNA in every experiment resulting in nine luciferase readings.

### Quantitative Real Time PCR (qPCR) and data analysis

200 to 1000 nanograms of RNA was reverse transcribed using Super Script III First-Strand Synthesis System kit (Invitrogen, USA) as described in Supplementary Methods. The RNA equivalent of cDNA so synthesized was stored at −20 °C.

Quantitative Real time PCR was performed using the standard SYBR Green method on ABI 7500 Fast Real Time PCR system (Applied Biosystems, USA) employing standard PCR conditions in triplicate as described in Supplementary Methods. The expressions of the target genes were normalised to endogenous controls like *18S rRNA*, *GAPDH*, or *β*-*ACTIN*. The mean Ct values of target genes were normalised to the mean Ct values of endogenous control for corresponding samples, which gives ∆Ct value. ∆∆Ct was calculated as (Test ∆Ct − Control ∆Ct). Fold change was calculated applying formula 2^−∆∆Ct^.

### Melanosome transfer

The keratinocytes, transfected with Pre-miRNA, TRP1 siRNA (Dharmacon GE Healthcare, USA) and scrambled control for 48 hours, were co-cultured with melanocytes (1:1 ratio) for 48 hours in M254 and KSFM media (1:1). Thereafter keratinocytes were harvested by differential trypsinization and analysed for melanosome transfer using pellet colour visualization and Melanin content estimation.

### Melanin Estimation

Melanin estimation was performed to assess the amount of melanosomes transferred to the keratinocytes after co-culture with melanocytes using standard NaOH method (Supplementary Materials for details).

### Statistical analysis

The statistical significance for various parameters was calculated using student’s t-test in Graph Pad Prism 5.0 software. p values of ≤0.05 were considered statistically significant.

## Supplementary information

Supllementary Materials

## Data Availability

Most of the data is available in Supplementary Materials. However, it is also available to be deposited whenever required.
