# Author Correction: Micro RNAs upregulated in Vitiligo skin play an important role in its aetiopathogenesis by altering TRP1 expression and keratinocyte-melanocytes cross-talk

**DOI:** 10.1038/s41598-020-58949-w

**Published:** 2020-02-04

**Authors:** Utpreksha Vaish, Avinash A. Kumar, Swati Varshney, Shreya Ghosh, Shantanu Sengupta, Chandni Sood, Hemanta K. Kar, Pankaj Sharma, Vivek T. Natarajan, Rajesh S. Gokhale, Rajni Rani

**Affiliations:** 1 National Institute of Immunology, New Delhi, 110067 India; 2 CSIR-Institute of Genomics & Integrative Biology, Mathura Road, Sukhdev Vihar, New Delhi, 110025 India; 3 Academy of Scientific and Innovative Research (AcSIR), CSIR-IGIB, Mathura Road, Sukhdev Vihar, New Delhi, 110025 India; 4 Dr. Ram Manohar Lohia Hospital, New Delhi, 110001 India

Correction to: *Scientific Reports* 10.1038/s41598-019-46529-6, published online 12 July 2019

In Figure 4c, the TRP1 blot for Sample 2 was inadvertently duplicated from the TRP1 blot for Sample 1. As a result, Figure 4e is incorrect. The correct Figure 4 appears below as Figure 1.

**Figure 1:**
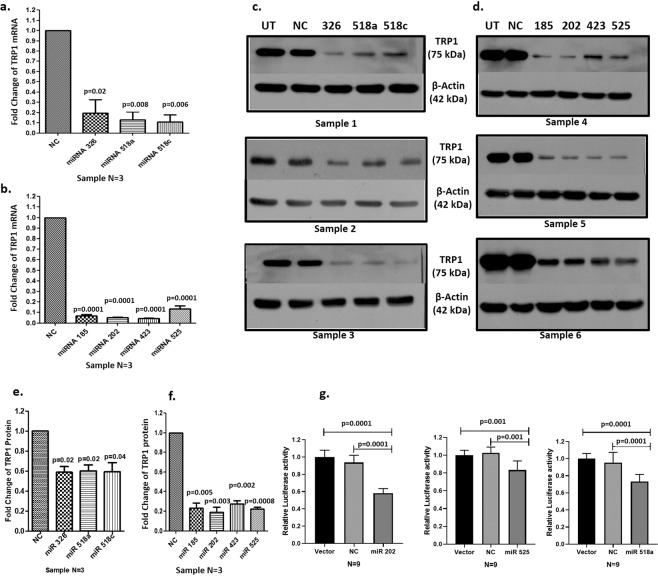
.

Additionally, in the Supplementary Information file, in the corresponding full blot image in figure S12 the TRP1 blot for Sample 2 was inadvertently duplicated from the TRP1 blot for Sample 1. The correct supplementary figure appears below as Figure 2.

**Figure 2:**
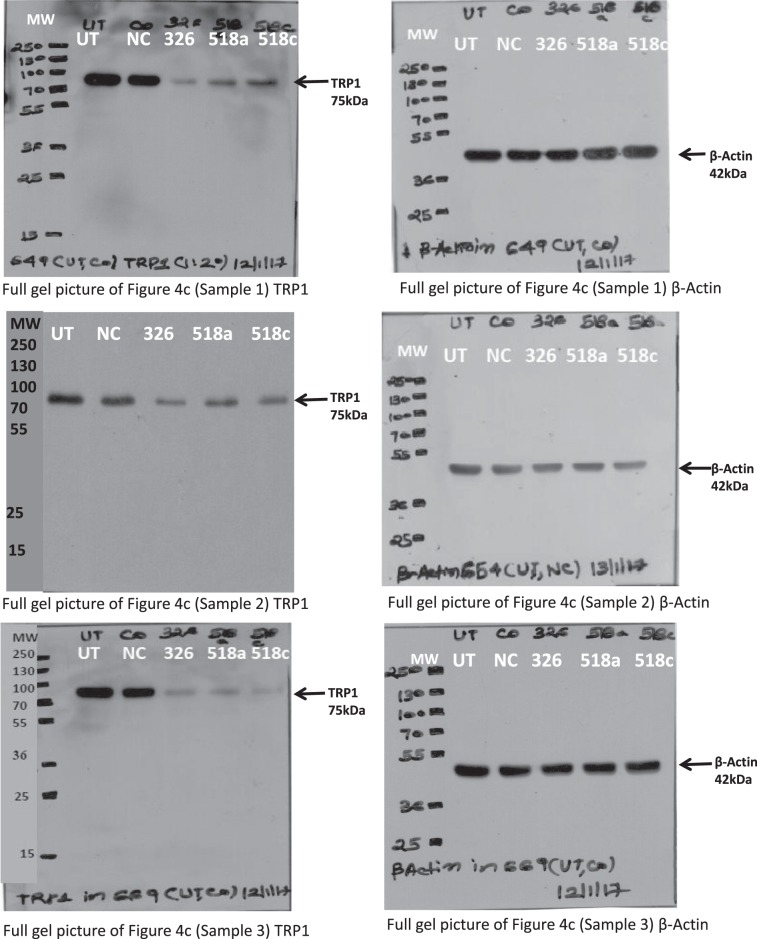
.

