# Supplementary material for: Micro RNAs upregulated in Vitiligo skin play an important role in its aetiopathogenesis by altering TRP1 expression and keratinocyte-melanocytes cross-talk
Source: Sci Rep. 2019 Jul 12;9:10079. doi: 10.1038/s41598-019-46529-6 (PMC6625998; doi:10.1038/s41598-019-46529-6)
Supplement: Supplementary file 1 — Supllementary Materials [file 41598_2019_46529_MOESM1_ESM.pdf]

Micro RNAs upregulated in Vitiligo skin play an important role in its aetiopathogenesis by altering TRP1 expression and keratinocyte-melanocytes cross-talk

Utpreksha Vaish[1], Avinash A Kumar[1], Swati Varshney[2,3], Shreya Ghosh[2], Shantanu Sengupta[2,3] , Chandni Sood[1], Hemanta K Kar[4] , Pankaj Sharma[4], Vivek T Natarajan[2,3], Rajesh S Gokhale[1,2], Rajni Rani[1*]

1. National Institute of Immunology
   New Delhi, 110067, India

2. CSIR-Institute of Genomics & Integrative Biology
   Mathura Road, Sukhdev Vihar
   New Delhi-110025, India

3. Academy of Scientific and Innovative Research (AcSIR), CSIR-IGIB,
   Mathura Road, Sukhdev Vihar
   New Delhi-110025

4. Dr. Ram Manohar Lohia Hospital
   New Delhi-110001, India

*Correspondence:

Dr. Rajni Rani
National Institute of Immunology
New Delhi-110067
India
Email: rajni@nii.ac.in

Supplementary Methods

Isolation of epidermis from skin biopsies

Skin punch biopsies (3 mm to 4 mm) from non-lesional and lesional regions were obtained from North Indian subjects (N=18) undergoing punch grafting/ melanocyte transplants at Dr. Ram Manohar Lohia Hospital (RMLH), New Delhi, after obtaining informed consent. The Human ethical review committee of RMLH and the National Institute of Immunology (NII), New Delhi, approved this study and is in agreement with the Declaration of Helsinki Principles. Biopsies were transported on ice and processed the same day by first washing with HBSS and surface sterilization in 70% ethanol for 1 minute followed by an HBSS wash. The biopsies were placed in 0.25% Dispase II solution and kept overnight at 4 °C. The epidermis was then removed using forceps and placed in Tri-reagent for RNA isolation or taken for single cell suspension for melanocyte-keratinocyte primary cultures.

Total RNA isolation from Tri-reagent

RNA was isolated from Tri-reagent according to the manufacturer's protocol. Briefly samples were homogenized in Tri-reagent and $1/5^{th}$ the volume of chloroform was added and mixed thoroughly. Samples were allowed to stand for 10 minutes and centrifuged at maximum RPM for 15 minutes at 4 °C. The aqueous supernatant was collected in a fresh tube, and precipitated with isopropanol (½ the volume of original Tri-reagent). The RNA pellet was collected by centrifugation at maximum RPM and was washed with 70% ethanol, semi dried and resuspended in 87.5µl Nuclease free water (NFW) (Ambion, USA). RNAs were purified using NucleoSpin TriPrep kit (MACHEREY-NAGEL, Germany) following the manufacturer's standard protocols. Highly pure RNA was quantified using the NANODROP 2000 Spectrophotometer and was run on a 1% agarose gel in TBE buffer to determine RNA integrity.

FlexmiR MicroRNA Assay and analysis

We used the FlexmiR MicroRNA Human Panel (version B) and FlexmiR MicroRNA labelling Kit (Luminex Corp., USA) to assay for differences in miRNA expression across non-lesional and lesional epidermal RNA. The assay was performed according to the manufacturer's instructions which included, first biotin labelling the RNA, and then hybridising them to the capture microspheres, before probing with streptavidin-PE and acquiring the signal on the Luminex IS 100. The entire assay procedure in brief is as follows: A minimum of 0.5-1.0 µg of labelled RNA is required per microsphere pool. Since we used five pools we labelled 2.75 µg of total RNA per sample in 11 µl total volume. To each sample we added CIP buffer (2.75 µl), CIP enzyme (2.75 µl), and non-biotinylated control RNA (2.2 µl). The reaction mix was placed in thermocycler with the following steps: 1) 37 °C 10 min 2) 95 °C 5 min 3) 4 °C end. The product was taken for biotinylation immediately, in which biotinylation buffer (16.5 µl) and biotinylation enzyme (8.25 µl) were added followed by incubation at 16 °C in a thermocycler for 60 minutes, and an inactivation step at 65 °C for 15 min. The labelled RNA was stored on ice till used in the hybridisation to FlexmiR human panel. The hybridisation was performed in 8-tube strips or PCR 96 well plates. 8 µl of microsphere pools (each pool separately) was added to background and sample wells. 14 µl hybridisation buffer and 8 µl labelled RNA was added to each reaction and made up to 50 µl. The reactions were incubated on a thermocycler at 95 °C for 3 min, followed by 60 °C for 1 hour before transferring to a 96-well filter plate (activated with wash buffer). Samples were washed using the vacuum manifold in the filter plates with wash buffer heated to 60 °C twice. The samples were resuspended in reporter solution and acquired on the Luminex IS 100. The data was analysed using a FlexmiR data analysis tool A02.29 which is an MS Excel macro-based tool. The background signal was subtracted, quality control beads verified, and normalised using 5 normalisation microspheres for various sno-RNA. From the

background subtracted, normalised data fold change was calculated by the formula: lesional signal/non-lesional signal for each miRNA for each sample pair. miRNA that gave a signal below 50 MFI in both lesional and non-lesional were ignored. miRNA where fold change was >1.5 were analysed in GraphPad Prism 5 for regulation using a paired T test.

miRNA transfection of NHKs

Pre-miR, transfection assay reagents and negative controls were obtained from Ambion (USA). They were transfected into normal human keratinocyte cultures by a reverse transfection procedure using SiPort Amine transfection reagent (Ambion, USA) according to the manufacturer's protocol. Briefly, NHKs in their 3rd passage were trypsinized and resuspended in complete KSFM at a density of 10^5 cells/ ml and transfected in six well plates at frequency of 0.20 x 10^6 cells/ well. 5 µl transfection reagent was diluted to 100 µl volume with OptiMem (Invitrogen) incubated for 10 min at RT. The pre-miRs at 6 µl (6.25 µM) per transfection well were also diluted to 100 µl with OptiMem. 100 µl of diluted transfection reagent and 100 µl of diluted pre-miRNA were mixed with a pipette and allowed to incubate at RT for exactly 10 minutes. The miR-reagent complex was dispensed in the wells of a 6-well culture plate, and 2 ml of diluted cell suspension was added to each well and mixed thoroughly. An untransfected control with only 200 µl OptiMem was also maintained. Cells were incubated at 37 °C and 5% CO_2 humidified incubator for 48 hours. Transfection efficiency was monitored using the fluorescent-labelled negative control (NC) using a fluorescent microscope (Olympus) in the RFP filter channel. After incubation, the cells were harvested for RNA and protein and also co-cultured with Melanocytes for 48 hours and thereafter Keratinocytes were harvested for Melanin estimation and Melanosome transfer.

Protein Isolation, Quantitation and iTRAQ labelling for proteomic analysis

Protein extraction was performed from samples like primary cells, cells harvested at the end of various experiments and lesional as well as non-lesional punch biopsies from Vitiligo patients. Protein was extracted using lysis buffer RIPA (Radio immune precipitation assay) supplemented with 1X MS-SAFE protease and phosphatase inhibitor. For cell cultures, RIPA was directly mixed with cell pellet whereas for patient samples the punch biopsies were churned with RIPA buffer with porcelain mortar and pestle on ice. Lysis was carried out at 4°C for 30 mins and thereafter sample was centrifuged at 4°C at 13000 RPM for 30 mins. Supernatant obtained after centrifugation was collected, as protein lysate in a separate eppendorf and stored at -80°C for further use.

The protein samples were estimated for their concentrations using Pierce BCA protein assay kit. A series of albumin standards were prepared in same diluents as that of protein samples to be tested. Working reagent was prepared and 200 µl was added to each well containing standards and unknown protein samples and the plate was incubated at 37°C for 30 min. The absorbance was measured at 562 nm in ELISA reader (Bio TEK, USA) and standard curve was drawn. The unknown protein sample's concentration was extrapolated from standard curve.

For iTRAQ labelling, 150 µg of protein samples from keratinocytes transfected with miRNAs (scrambled control and test sample) were taken, concentrated and buffer exchanged by 0.5M of Triethylammonium bicarbonate (TEAB) using 3 KDa Millipore centrifugal filters (Millipore, USA) at 4°C. The concentrated protein samples were again quantified. 50 µg of each protein was treated with 2 µl of 25mM of Dithiothreitol (DTT) (Sigma, USA) at 60°C for 30 min and then 1µl of 55mM Iodoacetamide (IAA) (GE Healthcare, USA) in dark for 15 min. Then, 2.25 µl of Trypsin (5 µg) (PROMEGA, USA) was added to each tube and samples were incubated at 37°C dry and shaking bath for 16-18 hours.

Next day, iTRAQ labels (AB Sciex, USA) were thawed on ice and resuspended in 70 µl of absolute ethanol. Then iTRAQ tags were gently vortexed, spun and added to respective samples. The samples were incubated at RT for 2 hours and reaction was quenched with 50 µl LCMS grade water (Fluka, USA). All samples of an experiment labelled with different tags were pooled, short spun, vacuum dried and resuspended in 1ml of 8mM Ammonium formate buffer and subjected to strong cation exchange chromatography. Elution was done by passing a series of Ammonium formate buffers in 30% Acetonitrile (ACN). All fractions were vacuum dried and run for LC MS/MS operation through AB SCIEX 6600 QTOF System and data was analysed in AB SCIEX protein pilot software which provided whole proteomic perturbations in test samples vs. scrambled control in xlsx format. The data so obtained was analysed for proteomic perturbations i.e. for relative expression of a protein in miRNA transfected keratinocytes normalised to scrambled control, as per following criteria, relative expression cut off ≤0.8 for downregulated proteins and relative expression cut off ≥1.2 for upregulated proteins and this way proteomics data was analysed.

Generation of Heat Map

Heat maps for the proteins regulated by different miRNA were created with Microsoft Excel 2007 software (Microsoft). First, the proteins regulated by each miRNA were arranged in increasing values of relative expression and thereafter "New rule" option of Conditional formatting function was used to assign colour code, as per the relative expression values i.e. 0 to 0.8 for downregulated proteins and 1.2 to 8.75 for upregulated proteins. Since our data set has highest relative expression as 8.74, we kept the scale for colour coding of upregulated protein as 1.2 to 8.75 and subsequently heat maps were generated for proteins involved in different pathways (Figure 2).

Western Blot analysis and densitometry

Western blot analysis was performed on proteins extracted in RIPA buffer. Protein preparation was resolved on SDS-PAGE gel (10% resolving and 5% stacking gel) and run at 25mA for 45 minutes in electrophoresis buffer. The resolved proteins were electroblotted onto a PVDF membrane (activated in absolute methanol for 2 minutes) using wet transfer (in transfer buffer containing 14.4g Glycine, 3.03g Tris and 20% methanol). The transfer was carried out for 90 minutes at 285mA at 4°C. The PVDF membrane containing the transferred proteins was then blocked with 5% NFDM (Non fat dried milk)/TBST or 5% BSA/TBST for 1 hour at RT and incubated overnight at 4°C with appropriate concentration of primary antibody. The membrane was then washed thoroughly with TBST and incubated with appropriate secondary antibody labelled with HRP (1;10000) for 1 hour at room temperature. ECL reagent was added and the signal was detected on an X-ray photographic film. The x-ray film was developed and fixed in the dark. Densitometry was done using ImageJ software and statistical significance calculated.

cDNA synthesis

Reverse transcription was performed using Super Script III First-Strand Synthesis System kit (Invitrogen, USA). Briefly, 8µl of RNA (generally 200 to 1000 nanograms of RNA), 1µl Random hexamers (50ng) and 1µl dNTP mix (1mM) were added and incubated at 65°C for 5 min. and then immediately chilled on ice for at least 1 min. To those tubes another master mix containing 2µl of 10X Reverse Transcription buffer (1X), 4µl of 25mM $MgCl_2$ (5mM), 2µl of 0.1M DTT (1mM), 1µl Superscript III enzyme (200U) and 1µl RNase OUT (40U) was added per reaction. The reaction mix was incubated first at 25°C for 10 min, for annealing followed by an extension step at 50°C for 1 hour. The RNA equivalent cDNA so synthesized was stored at -20°C.

Real Time PCR (qPCR) and data analysis

Quantitative Real time PCR was performed using the standard SYBR Green method on ABI 7500 Fast Real Time PCR system employing standard PCR conditions. The 15µl reaction was composed of 5ng RNA equivalent cDNA, 1X Maxima SYBR green (Thermo scientific, USA) and 0.2µM forward and reverse primer each (Sigma-Aldrich, India) (1µl of 3µM stock each). All the reactions were performed in triplicates. The expressions of the target genes were normalised to endogenous controls like *18SrRNA*, *GAPDH*, or *β-ACTIN*. The result files were extracted in XLSX format. The mean Ct values of target genes were normalised to the mean Ct values of endogenous control for corresponding samples, which gives ΔCt value. ΔΔCt was calculated as (Test ΔCt – Control ΔCt). Fold change was calculated applying formula $2^{-\Delta\Delta Ct}$. Student's t-test was employed to calculate the significance of difference between the test and the control samples using GraphPad Prism 5.0 software (GraphPad Software, La Jolla, CA, U.S.A.) and p value equal to or less than 0.05 was considered statistically significant.

Melanosome transfer studies

The keratinocytes were transfected with Pre-miRNA, TRP1 siRNA and scrambled control for 48 hrs as explained previously. After 48 hrs, the melanocytes were plated over these keratinocytes (1:1 keratinocytes to melanocytes) and further co-cultured for 48 hrs in mixture of M254 and KSFM media (1:1). At the end of 48 hours, the keratinocytes were harvested by differential trypsinization since melanocytes need less trypsinization to be eluted as compared to keratinocytes and analysed for melanosome transfer using total melanin estimation. After harvesting, equal number of keratinocytes were taken and lysed in 1N NaOH by incubating at 80°C water bath for 2hrs. In parallel, serial dilution of synthetic melanin was prepared in 1N NaOH and also incubated in same way. All the tubes were then centrifuged at maximum

speed for 10 min. The supernatant was subjected to absorbance measurement at 405nm.The melanin contents was deduced from the standard curve prepared from the synthetic melanin.

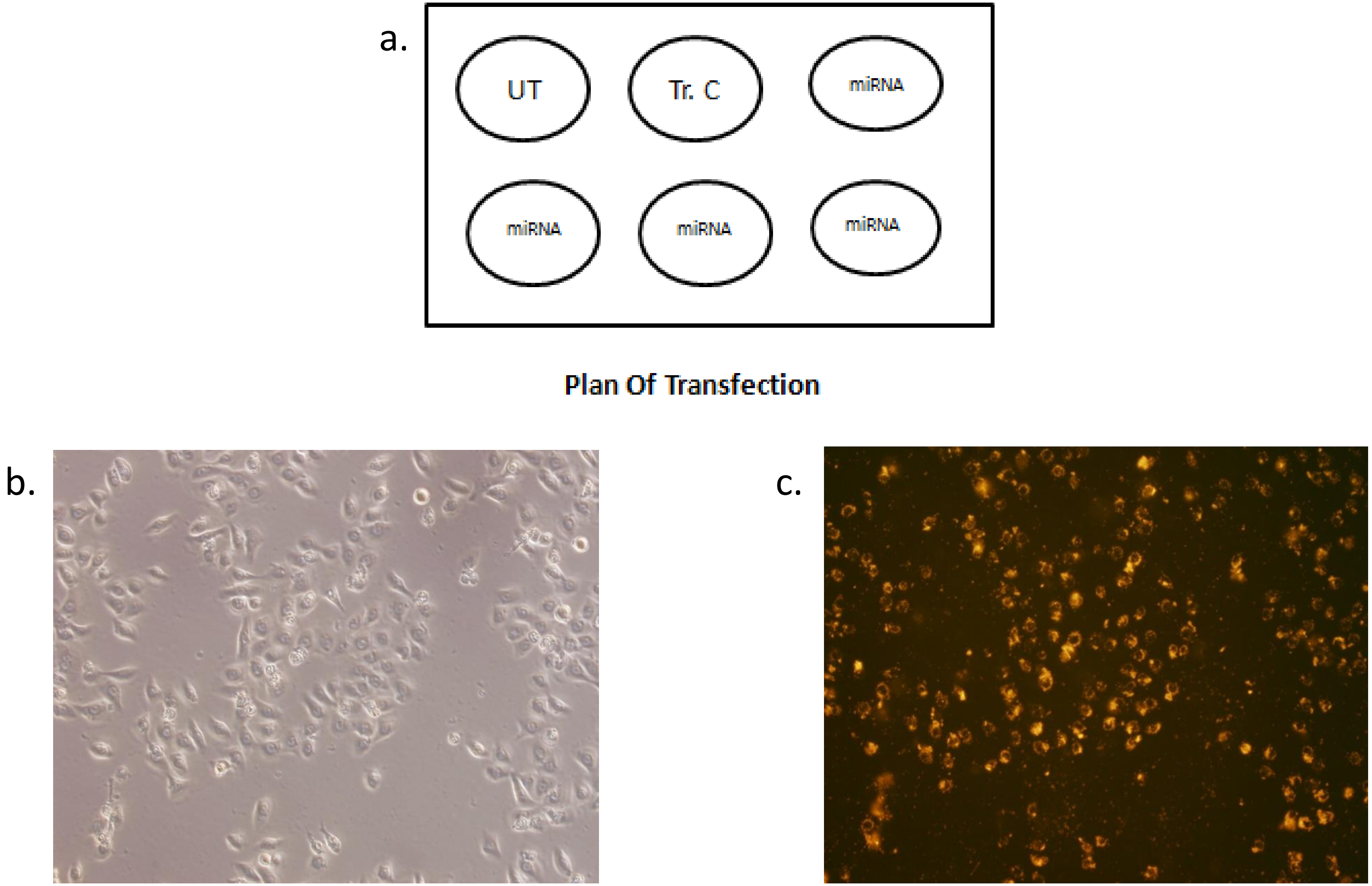

Supplementary Figure 1: Plan of Transfection and Transfection efficiency control: a. represents the plan of transfection where UT is Un-transfected Control and Tr. C is Transfection control or scrambled control. **b** and **c** are representative images showing transfection efficiency of pre-miRNA transfections as determined by the fluorescent Dy547-labelled transfection control, b. Bright field and c. WIGA (RFP) filter showing fluorescent transfected cells. Transfection Efficiency was more than 90%.

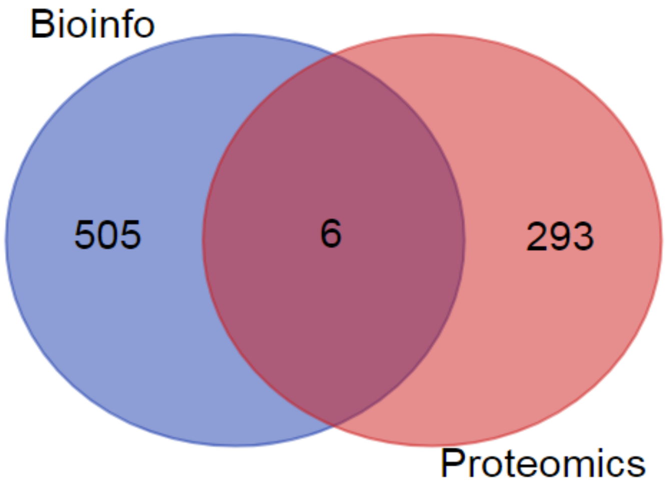

miRNA-326 : Common targets predicted by Target Scan and actually downregulated at proteomics level = 6
NAT10 ,MAPK1, DYNLL1, GLG1, LASP1 and PTBP1.

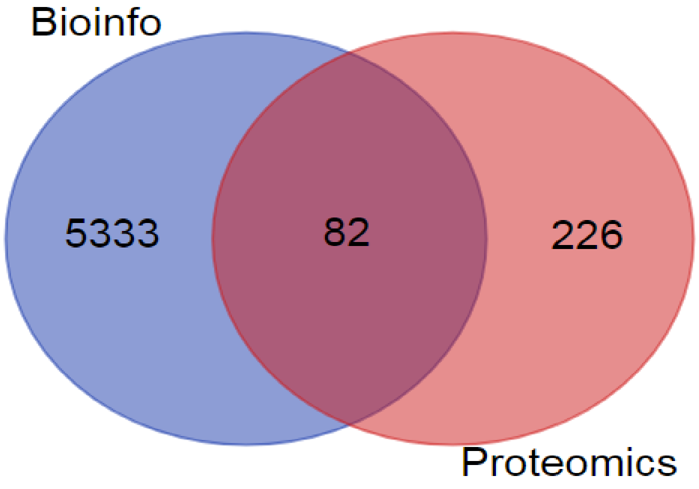

miRNA-518a: Common targets predicted by Target Scan and actually downregulated at proteomics level = 82
CD44, PTCD3, NAT10, GRPEL1, EIF1, PREP, GLG1, KRT15, RDX, LDLR, **TYRP1,** ACLY, STMN1, FAM3C, EIF2AK2, TNPO1, DNPEP, CFL2, RBBP7, HADHB, BICD2, CHP1, DEK, EEA1, NUP93, PAFAH1B1, OSTF1, USP10, HK1, CDC42, ALDH7A1, LASP1, ATAD3A, PTBP1, FXR1, NCKAP1, RTN4, OCIAD2, CD59, DNAJA2, PGRMC1, ATP5L, CANX, EIF2S2, KPNA6, H2AFY, CAND1, PALLD, ALCAM, MAPRE1, NOMO1, FAM114A2, HARS, ITGB1, ATP6V1A, PRKDC, IPO5, UBE2L3, BUB3, MAPK1, SNX9, H2AFV, NFKB1, UGGT1, PACSIN2, ACOX1, TARS, RBBP4, AK2, TXLNA, CHMP4B, KRT2, SEC23A, ARPC2, IKBIP, SERPINH1, NPEPPS, RALA, DLD, NOLC1, PFN1, CLIP1.

Supplementary figure 2. Venn Diagrams showing proteins predicted by Target Scan to be regulated and actually down regulated in Proteomics data by miR-326 and miR-518a. Common target proteins between prediction and proteomics data are listed above.

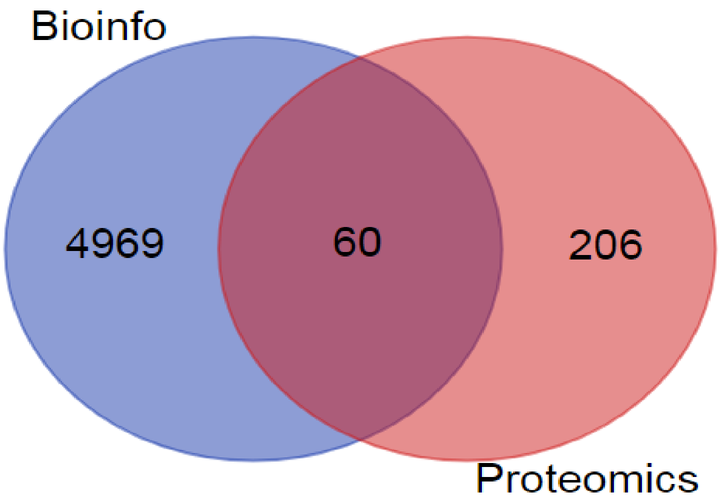

miRNA-518c: Common target Proteins predicted by target Scan and actually downregulated at proteomics level = 60
DECR1, FAHD1, ITGA3, UFL1, LDLR, NT5E, FAM213A, LRRFIP1, STMN1, NMT1, GNB1, NUP93, OSTF1, AHCY, ILK, SLC38A2, ALDH7A1, LASP1, CD59, SYK, RPL13, NDUFA9, CKAP5, KPNA6, PMPCA, EIF3M, FAM114A2, CNDP2, HARS, CORO1C, ATP6V1A, ITGA6, GLOD4, MYO1E, GFPT1, MAPK1, DYNLL1, CTPS1, CPOX, ARL8B, SYAP1, COPZ1, TOM1L2, TARS, VAPB, PSMB2, STRAP, VDAC1, CTSD, AK2, TXLNA, FAF2, GAPVD1, TPM4, CAPRIN1, RALA, SORD, VAMP3, ARF3, ANXA11.

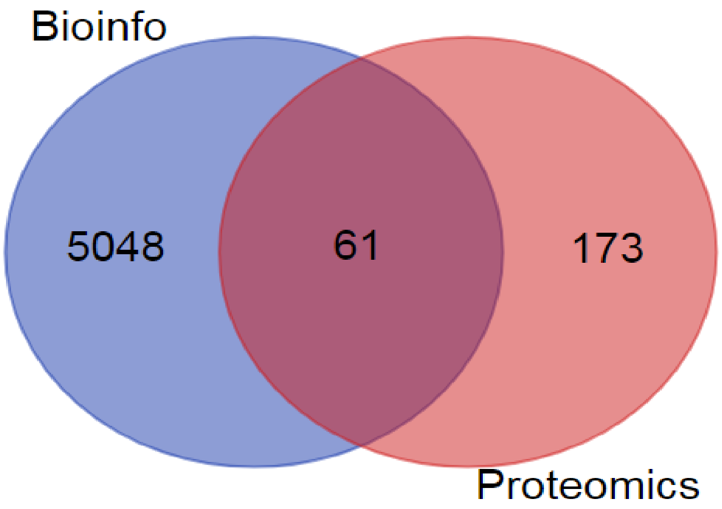

miRNA-185: Common target Proteins predicted by Target Scan and actually downregulated at proteomics level = 61
SOD2, PROSC, ITGA3, MAOA, RPS28, PDAP1, FIS1, NT5E, EIF2AK2, RPL28, OAS2, CDC37, PPP2CA, JUP, MYBBP1A, RCN2, DBNL, PDLIM7, ACACA, PLCG1, PRKAR2A, SKP1, ALDH4A1, PI4K2A, ILK, ANXA6, MYO18A, NAPA, CIRBP, ATP1B3, NDUFS1, GSS, RAB35, **RAB5B**, FAM83H, TOR1AIP1, HNRNPA0, HARS, VPS35, PFAS, BLVRA, RBP2, RPS6KA1, ELAVL1, USP5, RPL11, PCBP2, MTCH2, PLOD1, IDH2, PLCB3, INF2, LMOD1, RBBP4, CTSD, LIMK2, TTLL12, SERPINB6, PKM, NAPRT1, ANXA11.

Supplementary figure 3. Venn Diagrams showing proteins predicted by Target Scan to be regulated and actually down regulated in Proteomics data by miR-518c and miR-185. Common target proteins between prediction and proteomics data are listed above.

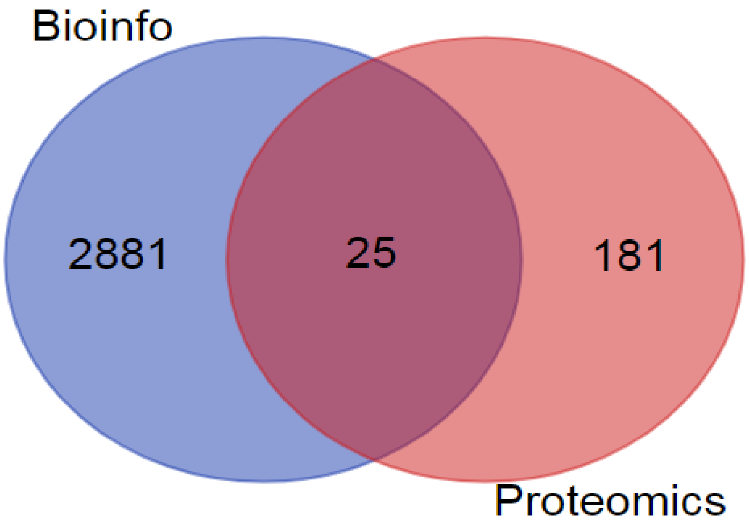

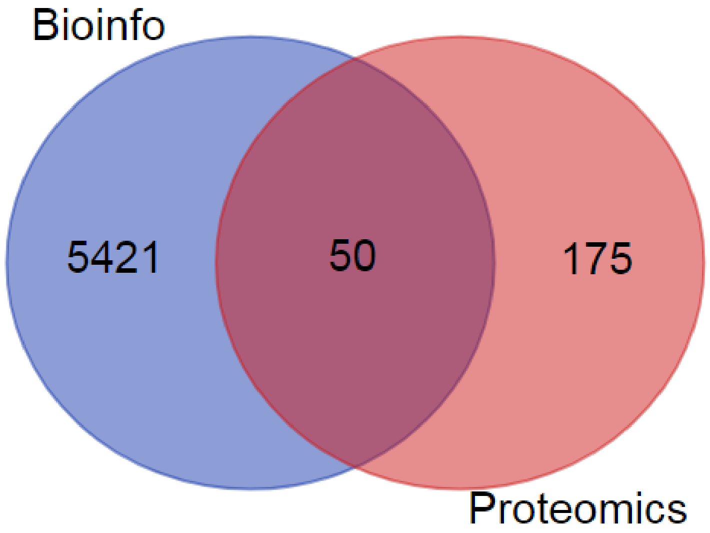

miRNA-202: Common target Proteins predicted by Target Scan and actually downregulated at proteomics level =25
CD44, DDB1, IGF2BP3, NDUFS1, SOD2, ATL2, TPBG, **TYRP1**, EIF2AK2, CFL2, ABCF1, SLC25A10, ERC1, GMPR2, AIFM1, SKP1, MYO6, AKR7A2, NAA10, EIF3F, GTF2F1, FEN1, ATP6V1G1, NUDT5, GIPC1.

miRNA-525: Common target Proteins predicted Target Scan and actually downregulated at proteomics level = 50
CD44, ITGA3, GCLM ,TPBG, RDX, **TYRP1**, ARSA, LMNB1, PPP5C, API5, ABCF1, RPL18A, DDX21, PPP2CA, LPP, ACACA, LYPLA1, FAS, PI4K2A, AIM1, LDHA, GNB2, NCKAP1, NUCB2, SEC24C, DUT, CKAP5, RANGAP1, NDUFS1, **RAB5B**, YWHAH, GFPT1, PFAS, ACOT13, SF3A1, MUT ,TCEB1, GORASP2, ELAVL1, ,USP5, ERC1, EIF3B, IDH2, RPS3, AP2M1, MRRF, KPNA1, GNB2L1, CLNS1A, UBE2D2.

Supplementary figure 4. Venn Diagrams showing proteins predicted by Target Scan to be regulated and actually down regulated in Proteomics data by miR-202 and miR-525. Common target proteins between prediction and proteomics data are listed above.

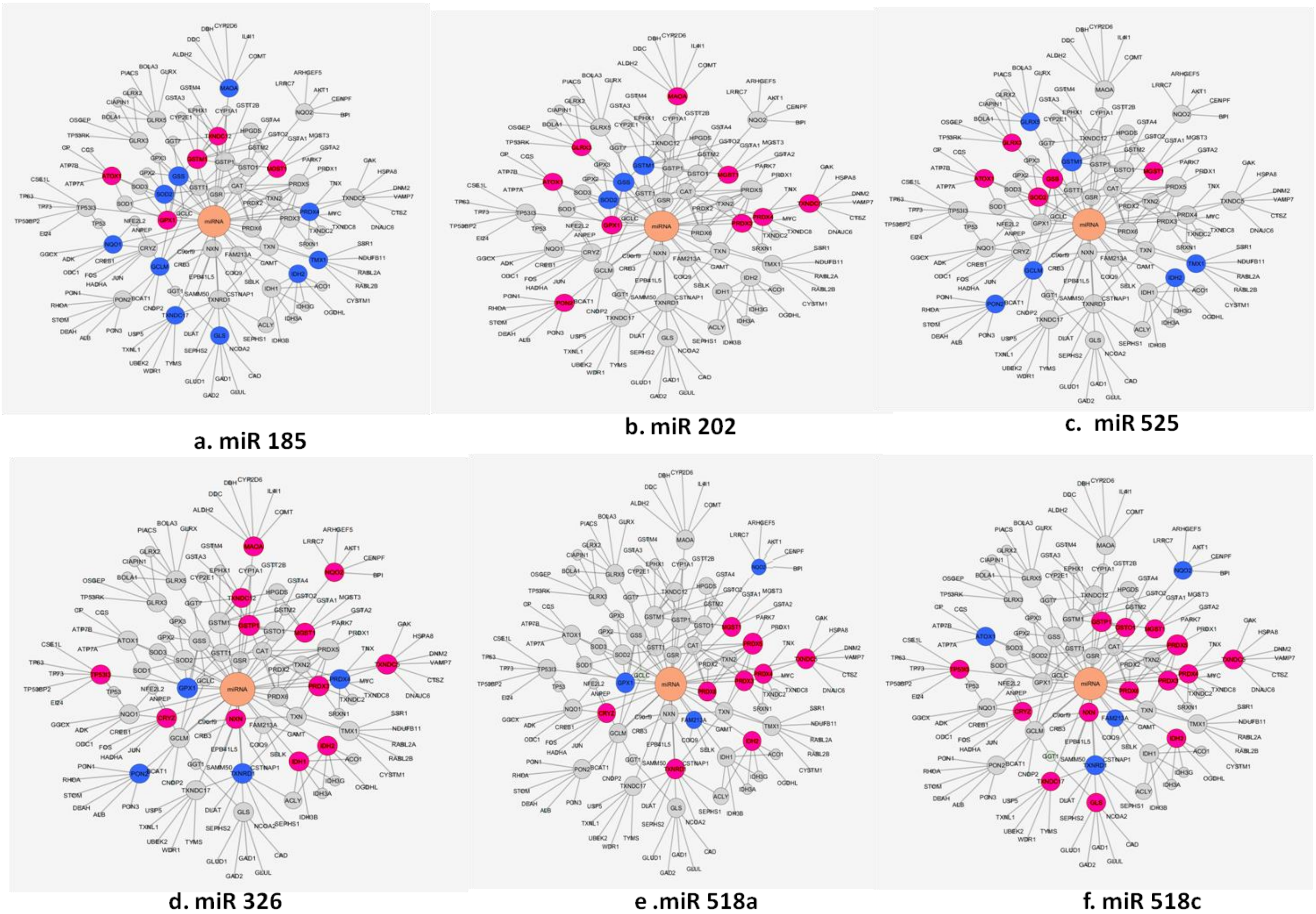

Supplementary figure 5. Regulation of Oxidative Stress network by six micro RNAs as revealed by iTRAQ mass spectrometry. Pink circles depict upregulated, blue downregulated and grey not regulated proteins.

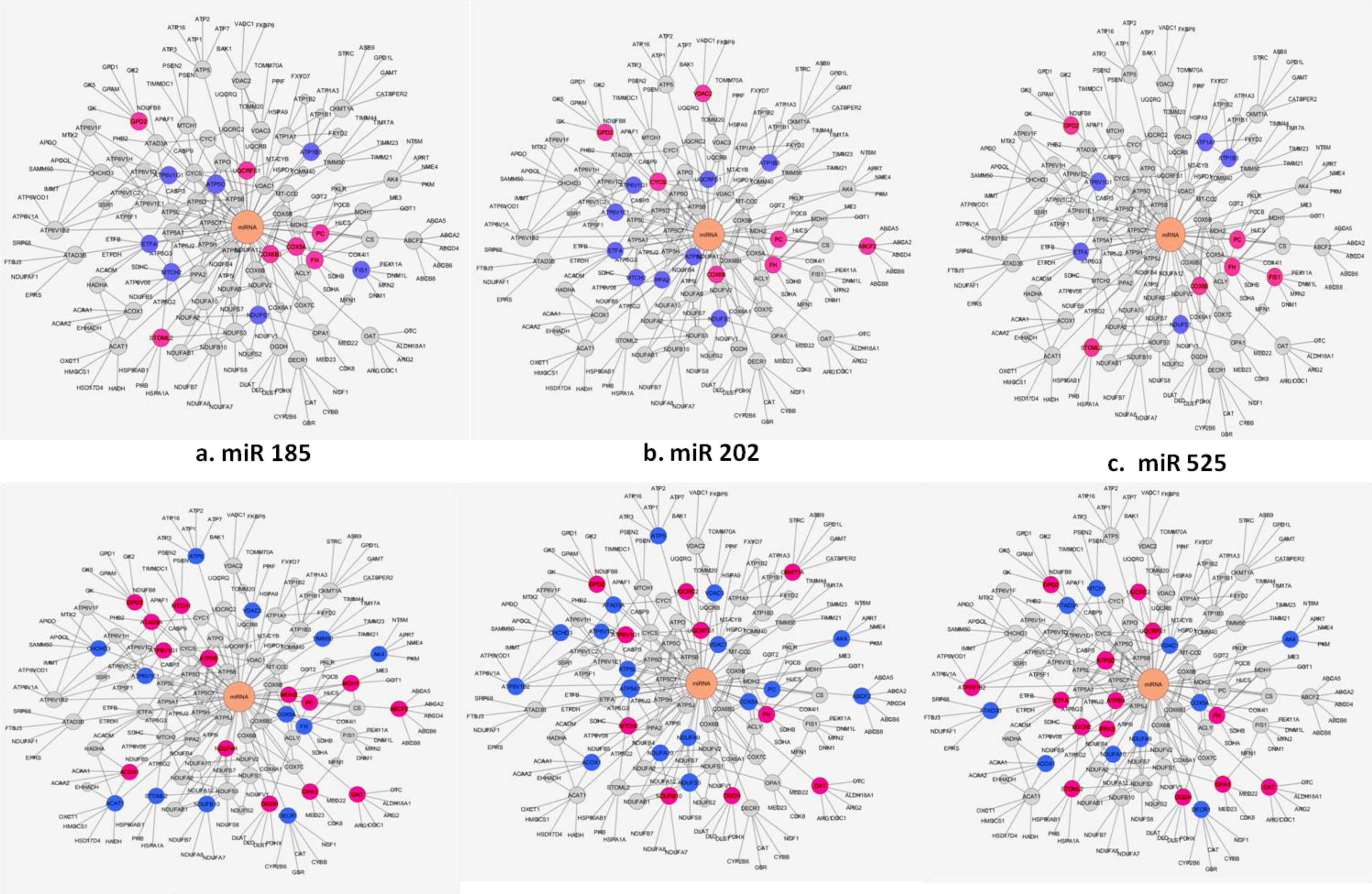

a. miR 185

b. miR 202

c. miR 525

d. miR 326

e .miR 518a

f. miR 518c

Supplementary figure 6. Regulation of Mitochondrial proteins network by six micro RNAs as revealed by iTRAQ mass spectrometry. Pink circles depict upregulated, blue downregulated and grey not regulated proteins.

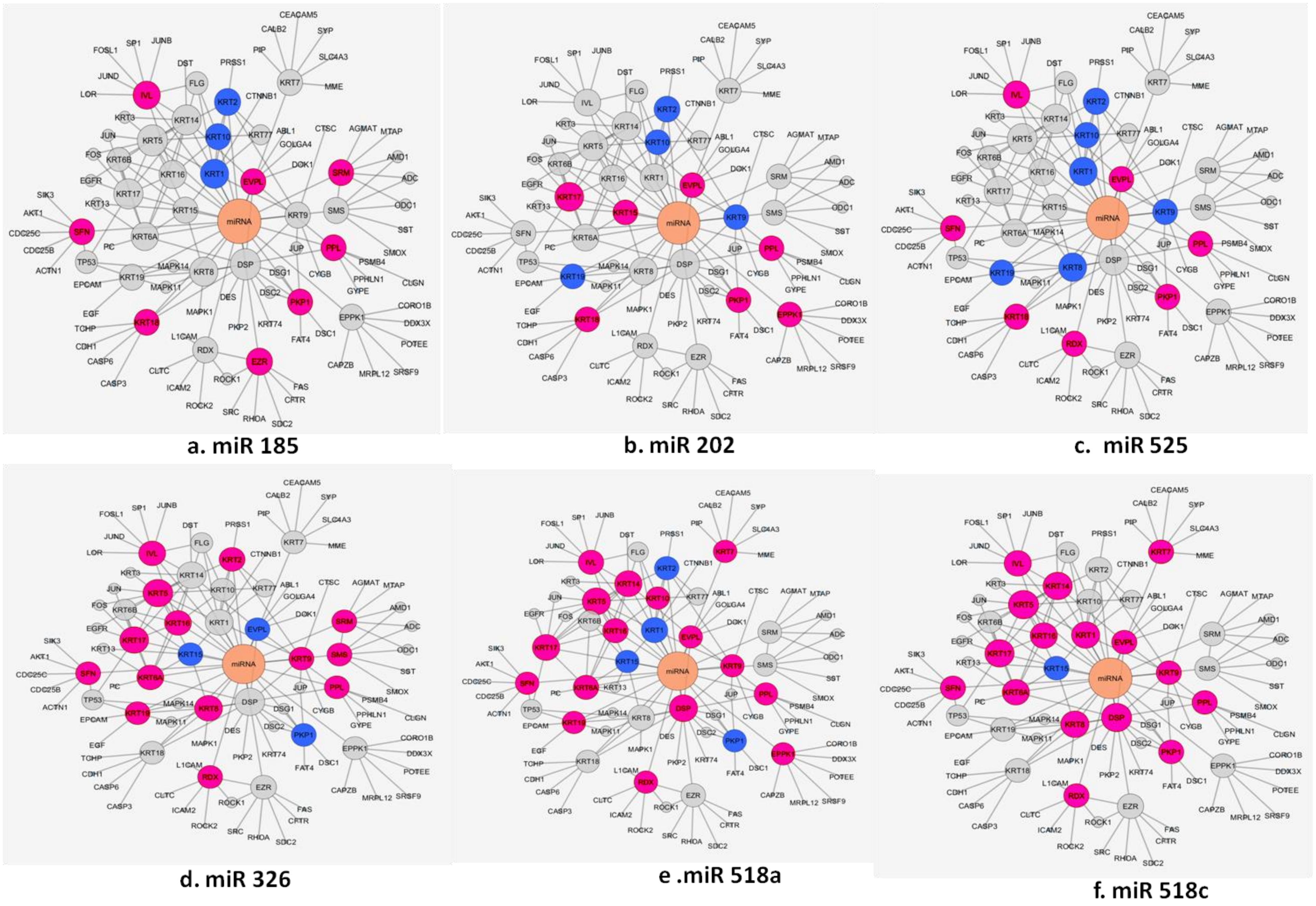

Supplementary figure 7. Regulation of Keratins and structural proteins by six micro RNAs as revealed by iTRAQ mass spectrometry. Pink circles depict upregulated, blue downregulated and grey not regulated proteins.

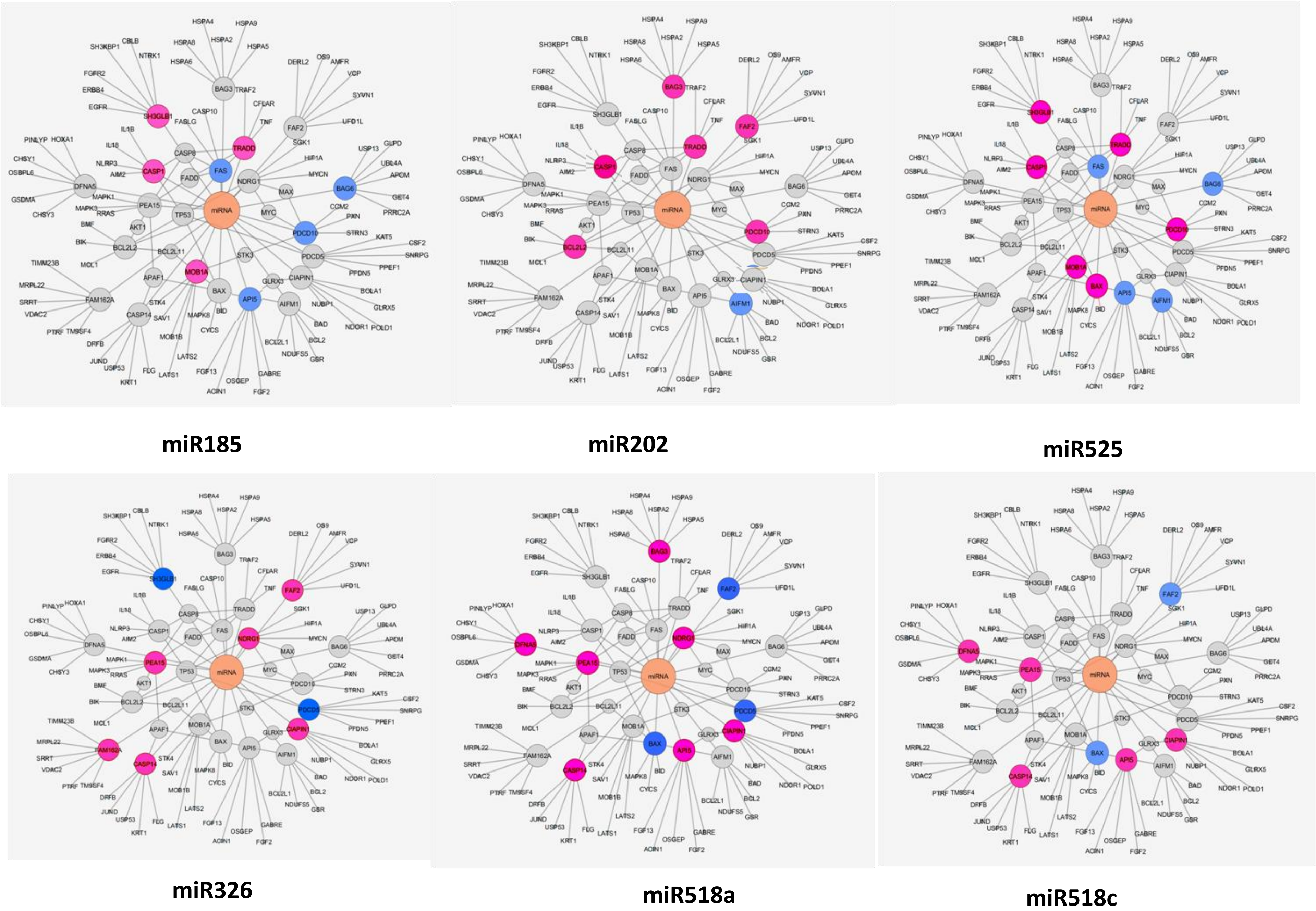

Supplementary figure 8. Regulation of Apoptosis network by six micro RNAs as revealed by iTRAQ mass spectrometry. Pink circles depict upregulated, blue downregulated and grey not regulated proteins.

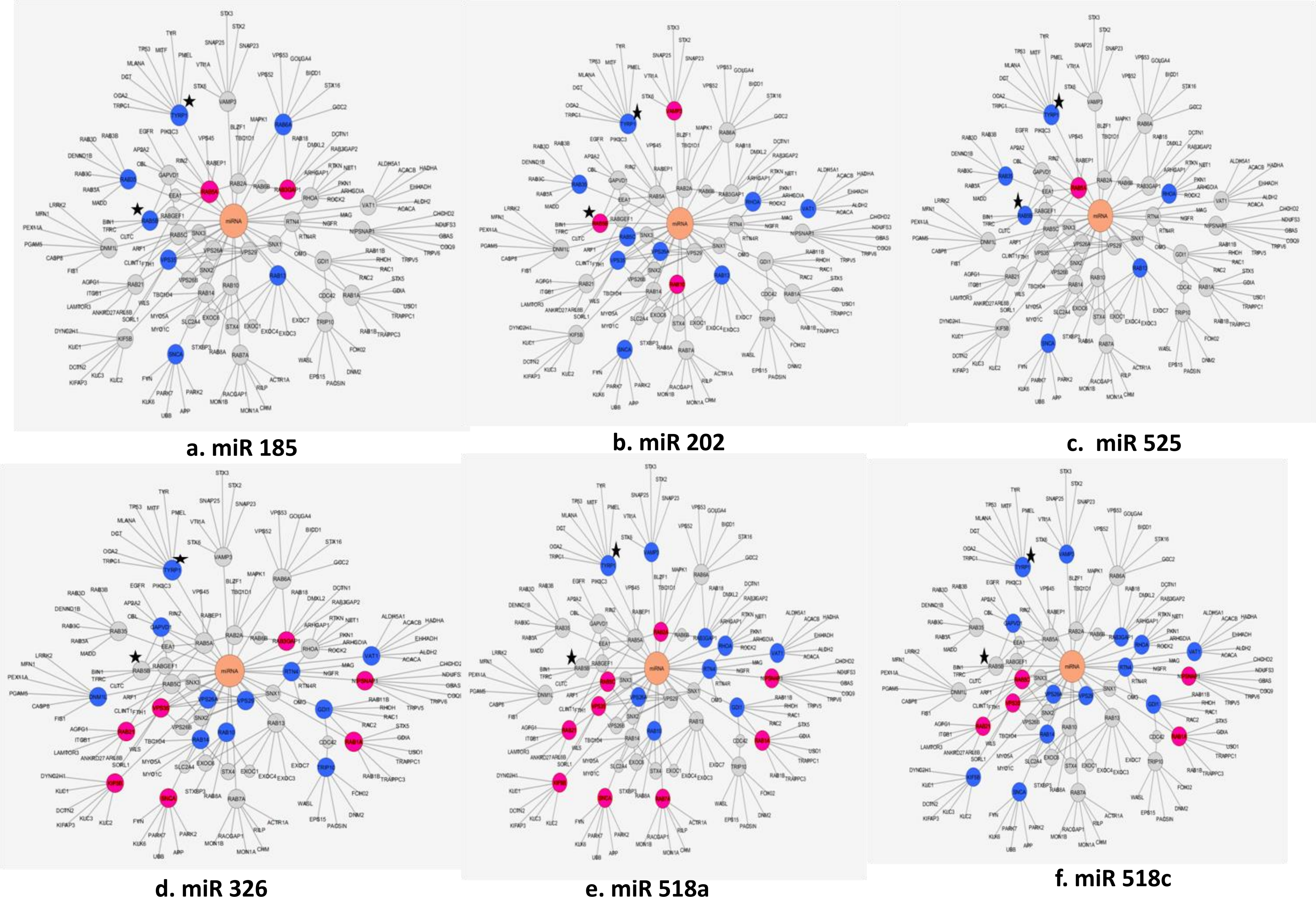

Supplementary figure 9. Regulation of Vesicular and trafficking partners by six micro RNAs as revealed by iTRAQ mass spectrometry. Pink circles depict upregulated, blue downregulated and grey not regulated proteins. * shows TRP1 and RAB5b proteins regulation.

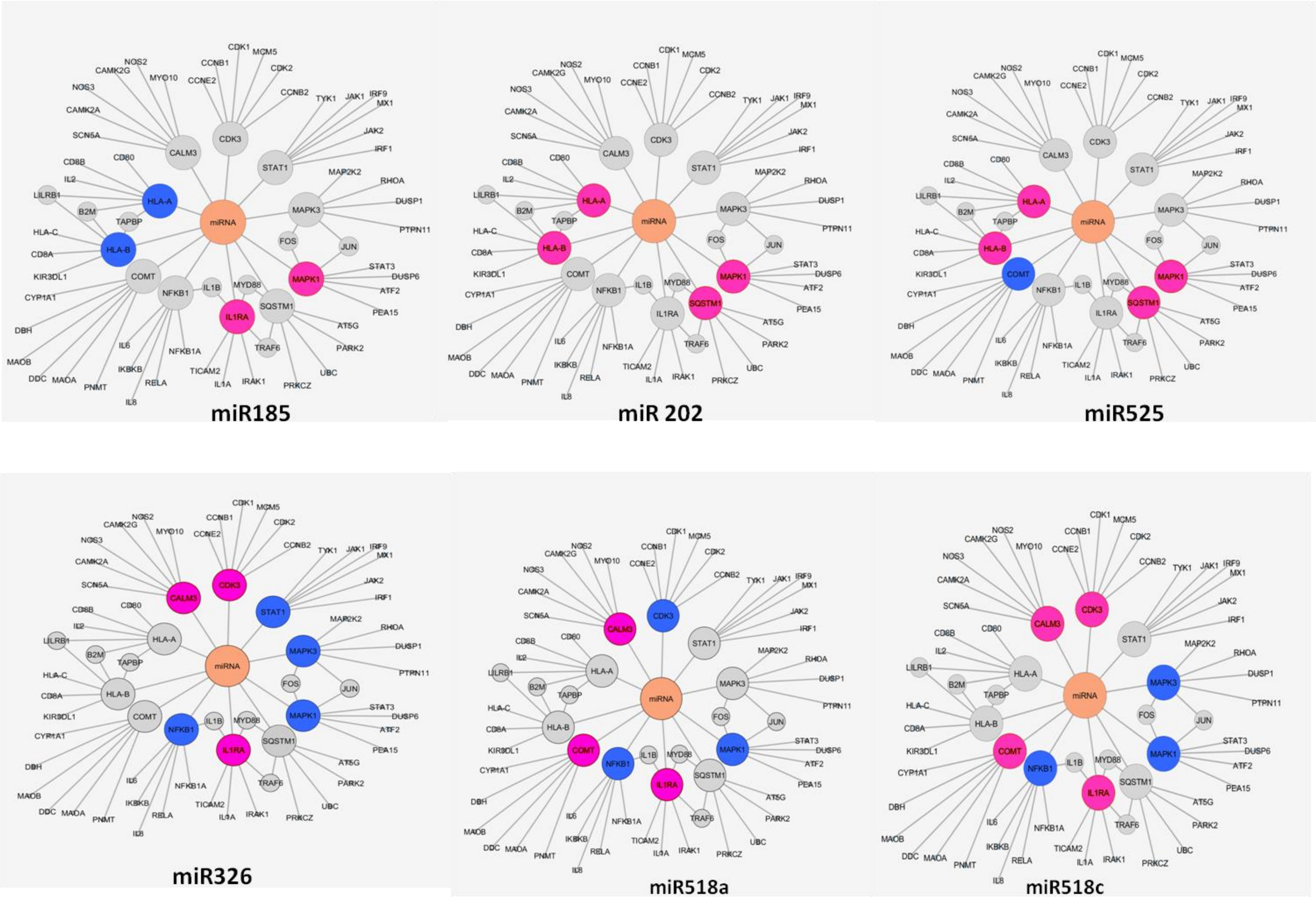

Supplementary figure 10. Regulation of Immune system related proteins and kinases by six micro RNAs as revealed by iTRAQ mass spectrometry. Pink circles depict upregulated, blue downregulated and grey not regulated proteins.

a.

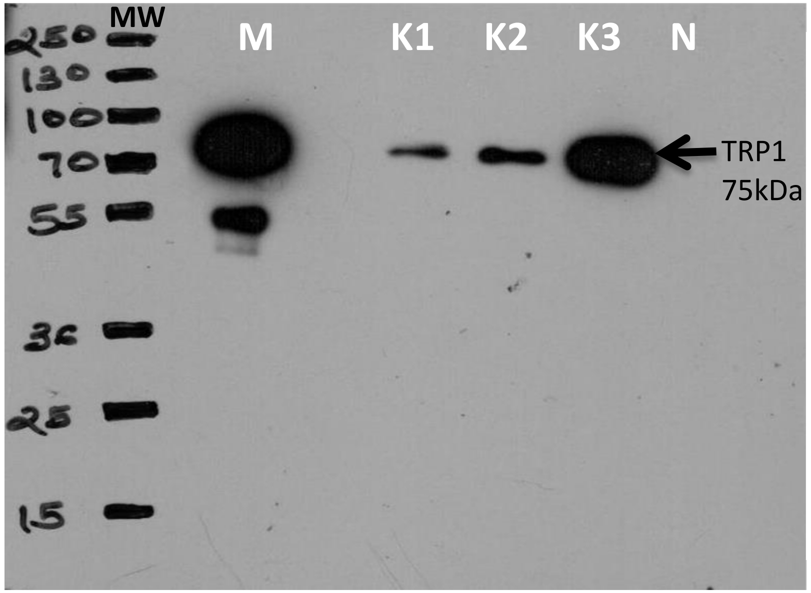

Full gel of Figure 3d TRP1(Exposure time:2 Seconds)

b. Anti-TRP1 ab17867 (Abcam)

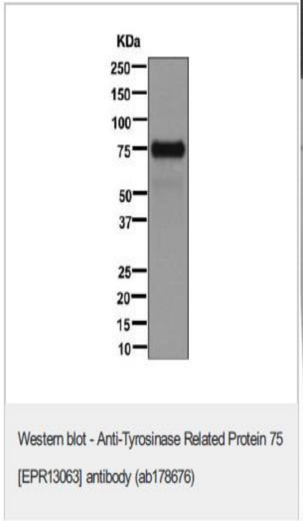

c.

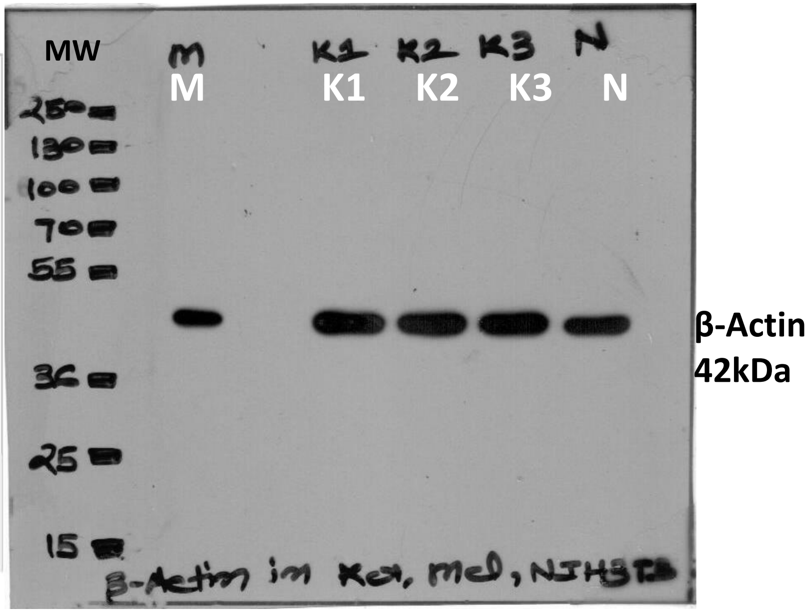

Full gel of Figure 3d β-Actin
(Exposure time: 1-2 Seconds)

d.

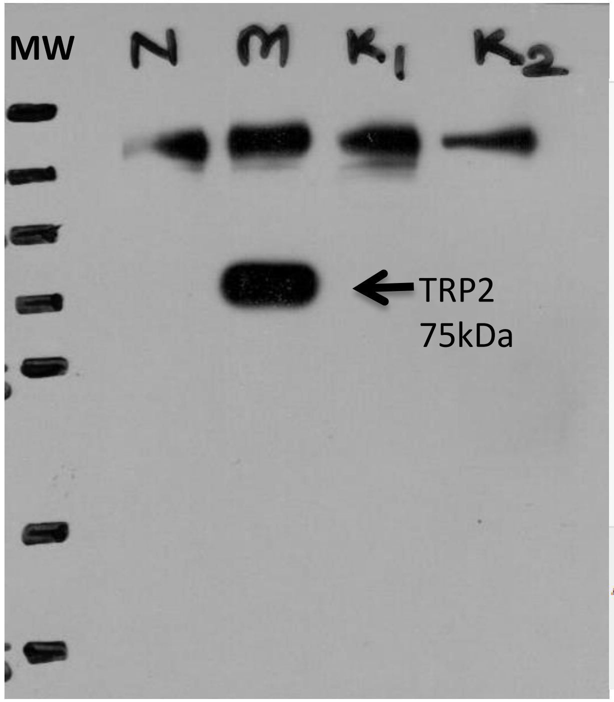

Full gel of Figure 3e TRP2

e. Anti-TRP2 ab74073 (Abcam)

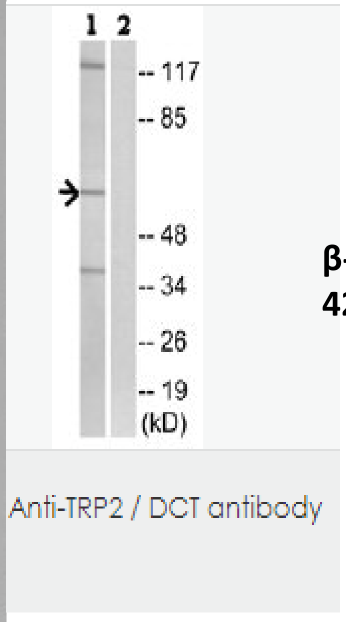

f.

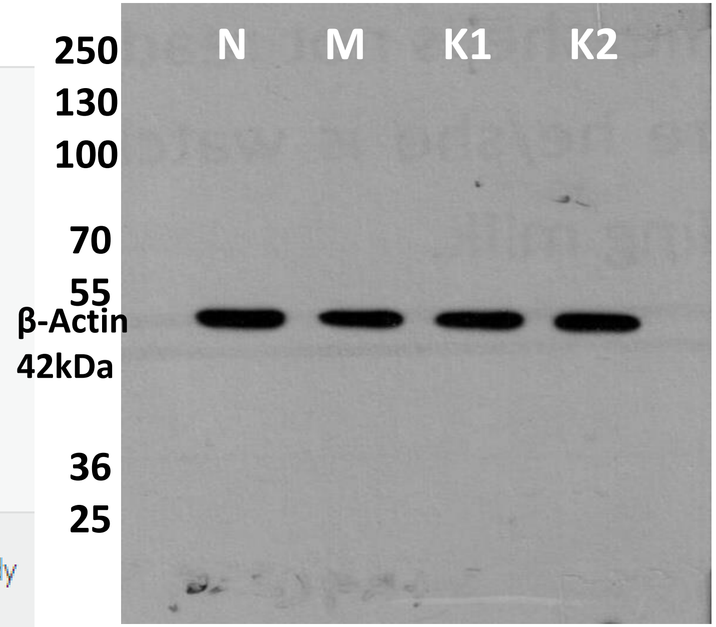

Full gel of Figure 3e β-Actin

Supplementary figure 11

Supplementary figure 11. Full gel pictures for figures 3 c and d showing expression of TRP1 (a), beta actin (c, f) and TRP2 (d) at protein level, along with the predicted sizes shown in the product insert (b, e) for the specific antibodies used. MW is molecular weight marker. Full gel picture of Figure 3c shows expression of TRP1 at protein level in Keratinocyte cell lysate in K1, K2 and K3 samples showing variable expression. M is Melanocyte lysate as positive control where TRP1 expression is very abundant whereas N is NIH3T3, negative control where there is no expression of TRP1. c. Full gel picture of figure 3d showing expression of TRP2/DCT in melanocytes only and not in keratinocytes and negative control.

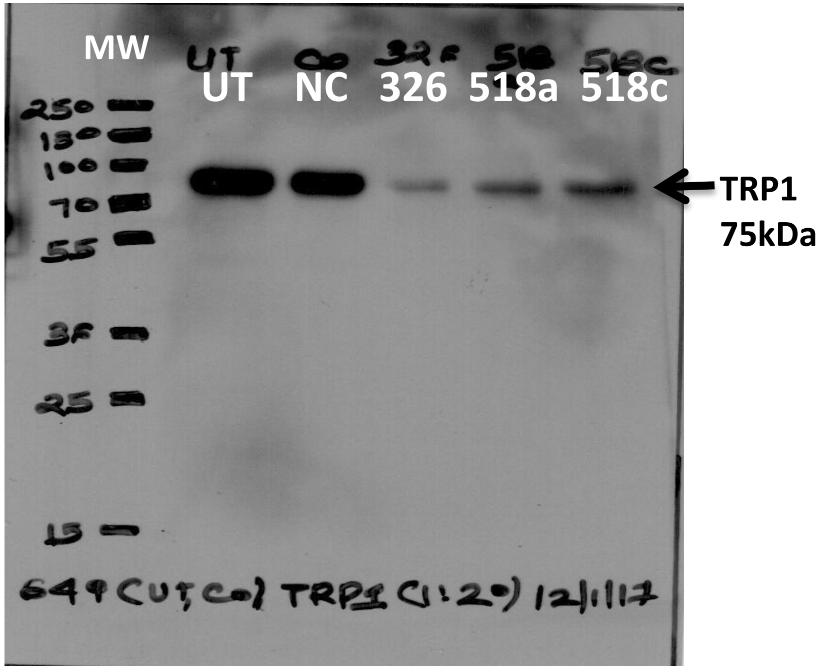

Full gel picture of Figure 4c (Sample 1) TRP1

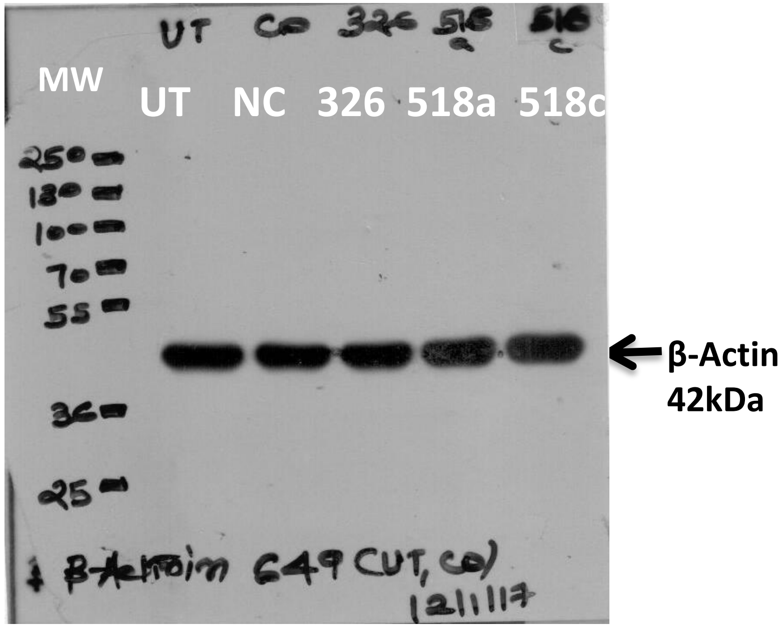

Full gel picture of Figure 4c (Sample 1) β-Actin

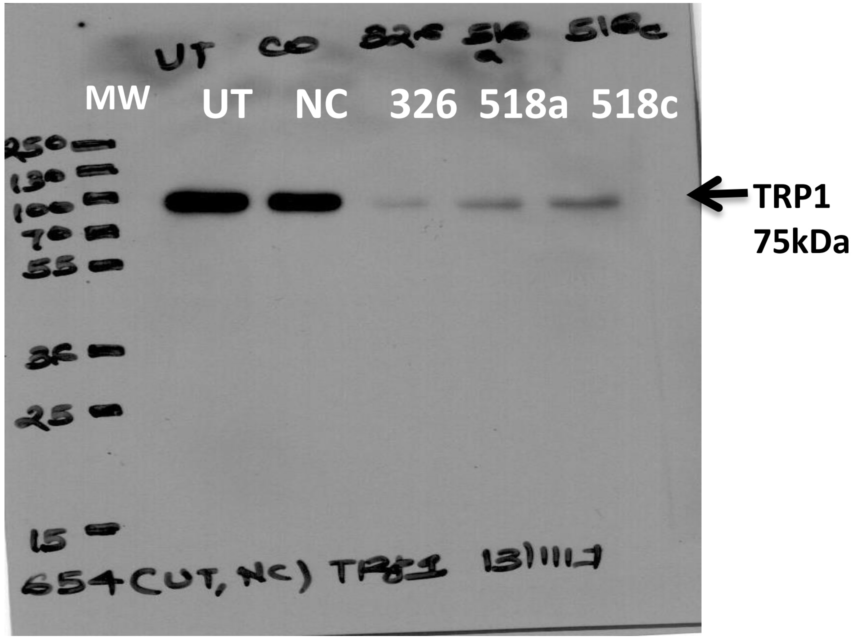

Full gel picture of Figure 4c (Sample 2) TRP1

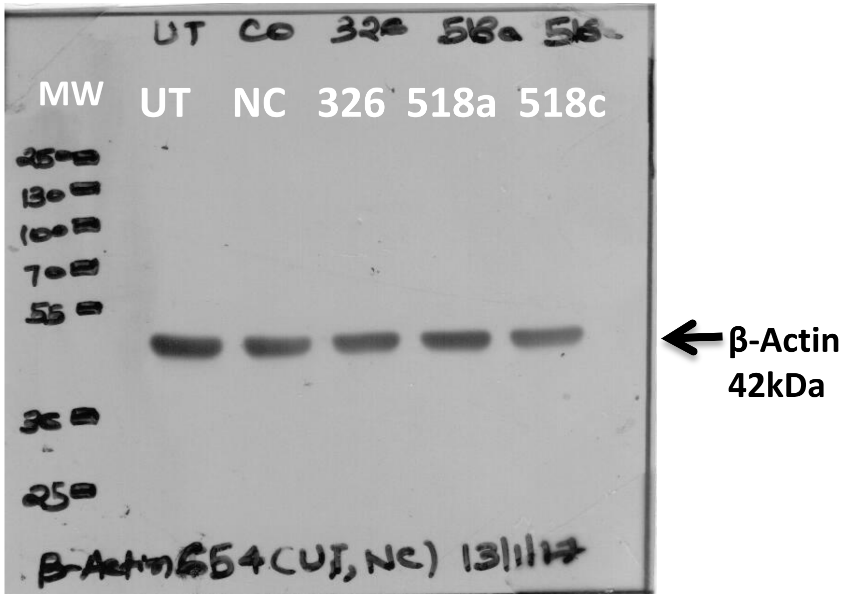

Full gel picture of Figure 4c (Sample 2) β-Actin

Supplementary figure 12

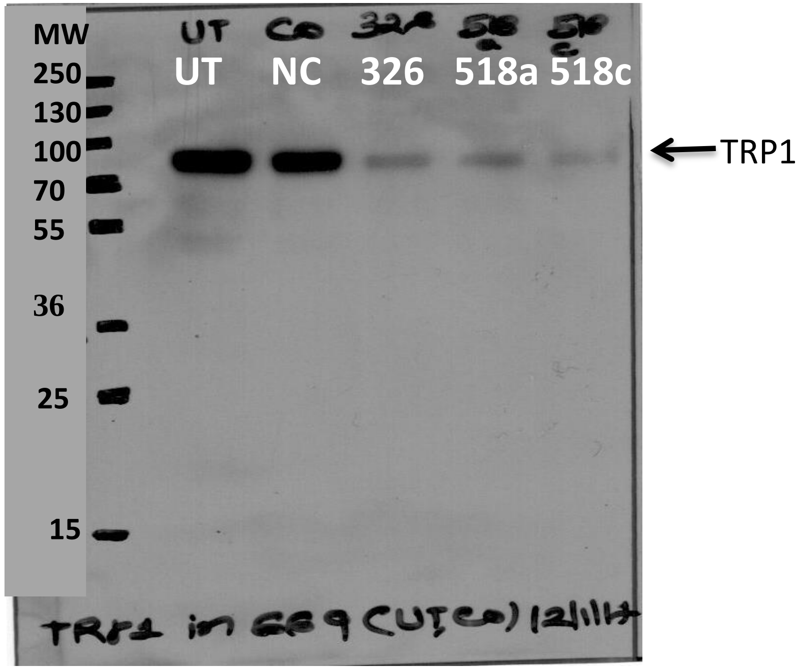

Full gel picture of Figure 4c (Sample 3) TRP1

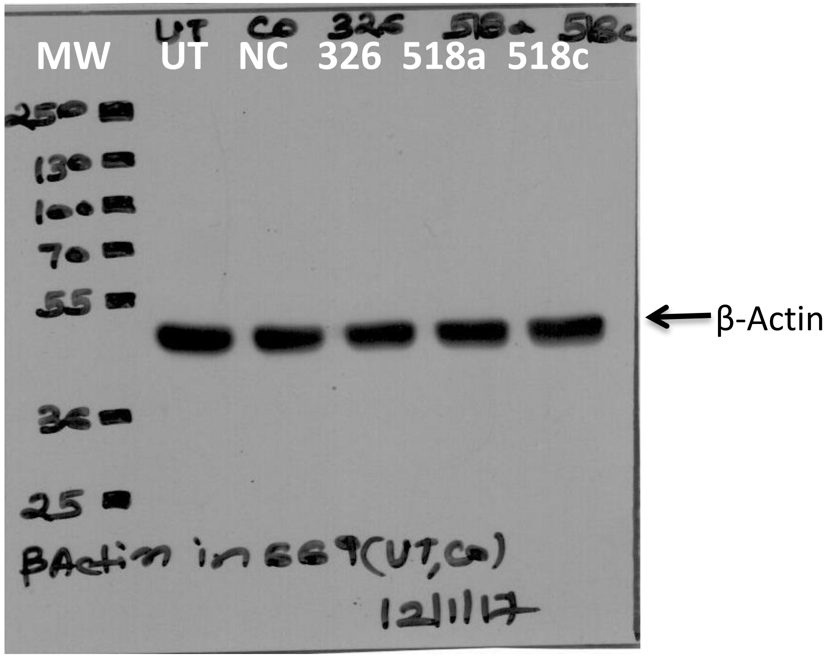

Full gel picture of Figure 4c (Sample 3) β-Actin

Supplementary figure 12. Full gel pictures of figure 4c showing Western blot of TRP1 and β-actin expression in three keratinocyte samples after transfection with pre-miR-326, pre-miR-518a, pre-miR-518c. UT is Untransfected control. NC is scrambled negative control.

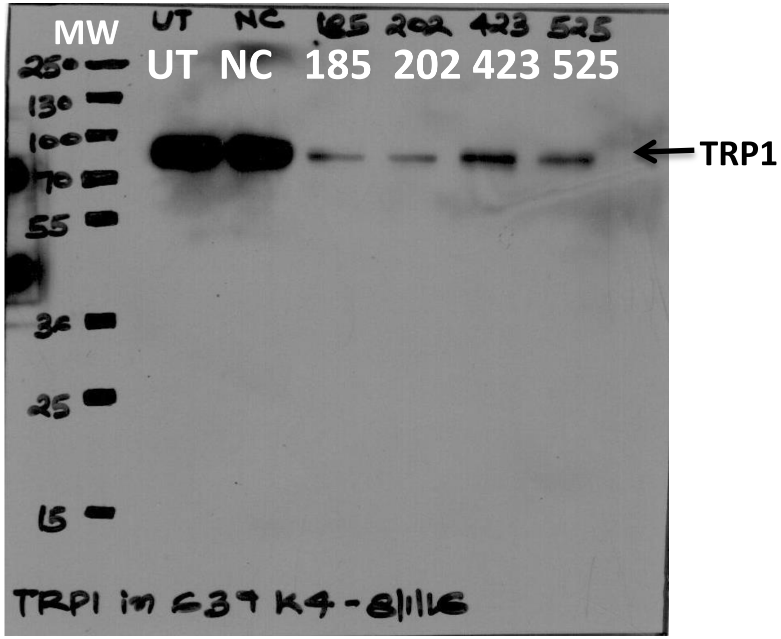

Full gel picture of Figure 4d (Sample 4) TRP1

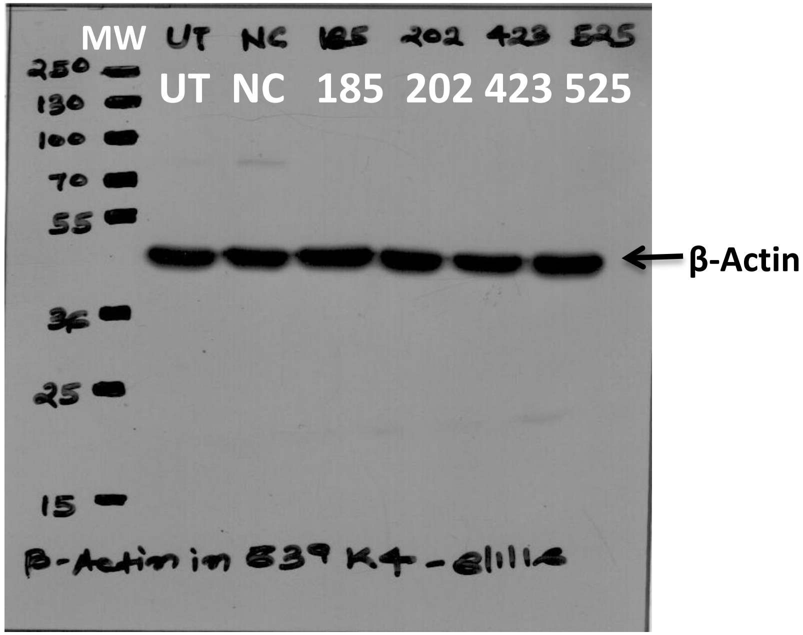

Full gel picture of Figure 4d (Sample 4) β-Actin

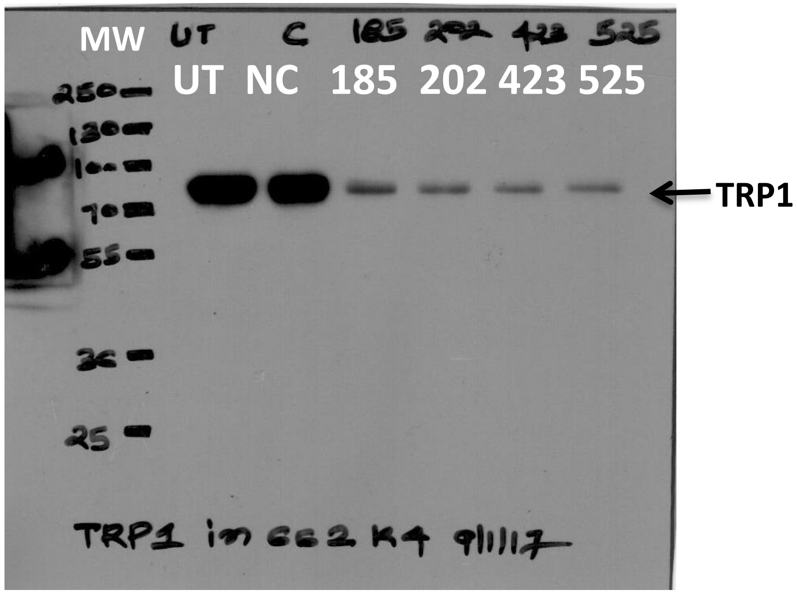

Full gel picture of Figure 4d (Sample 5) TRP1

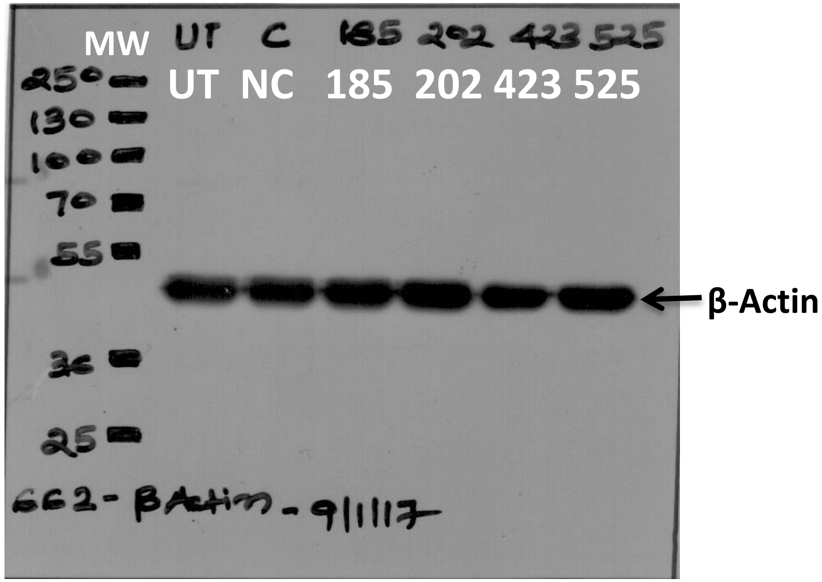

Full gel picture of Figure 4d (Sample 5) β-Actin

Supplementary figure 13

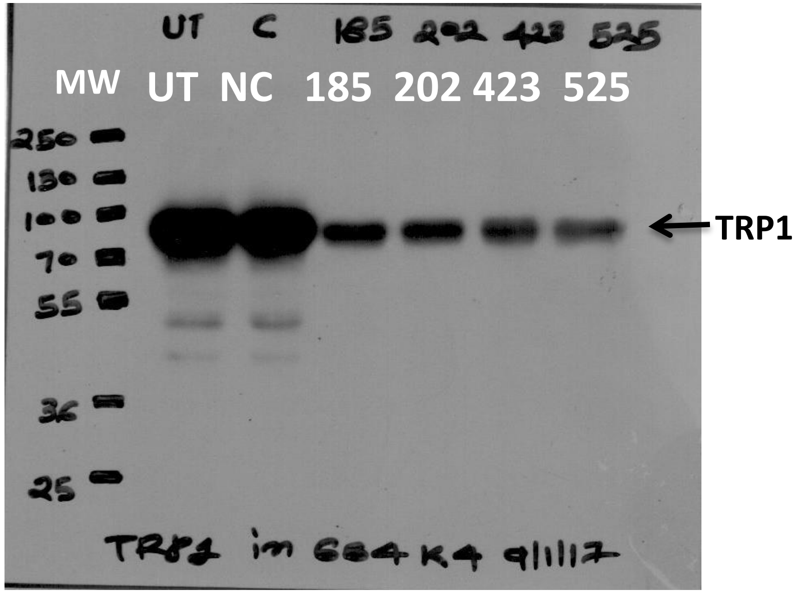

Full gel picture of Figure 4d (Sample 6) TRP1

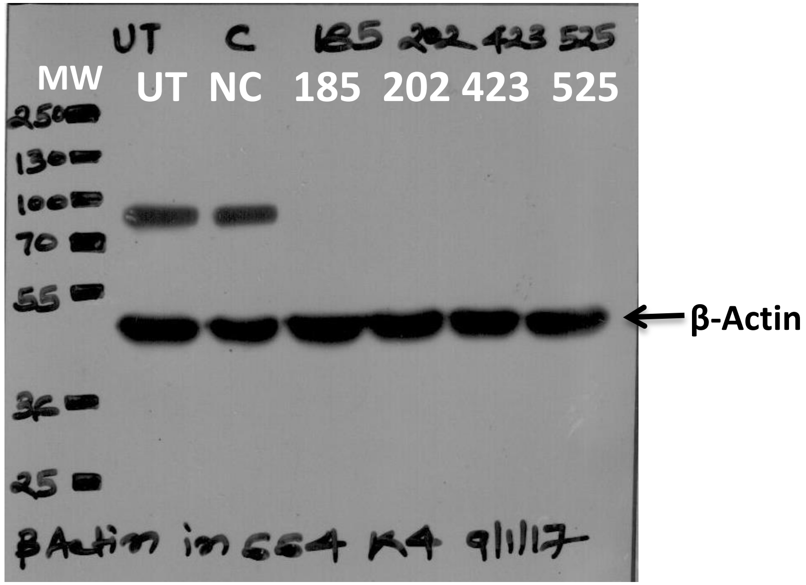

Full gel picture of Figure 4d (Sample 6) β-Actin

Supplementary figure 13. Full gel pictures of figure 4d showing expression of TRP1 and β-actin in three keratinocyte samples transfected with miRNA 185, 202, 423 and 525. MW is molecular weight marker

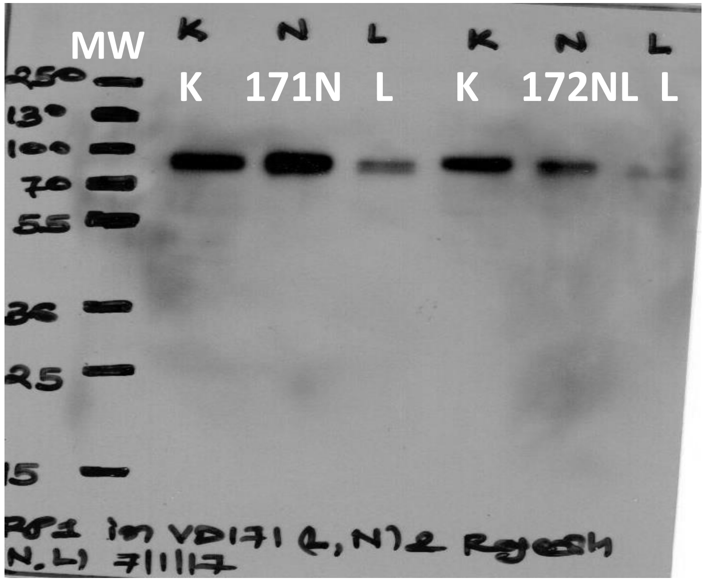

TRP1

Full gel picture of 5d (1) TRP1

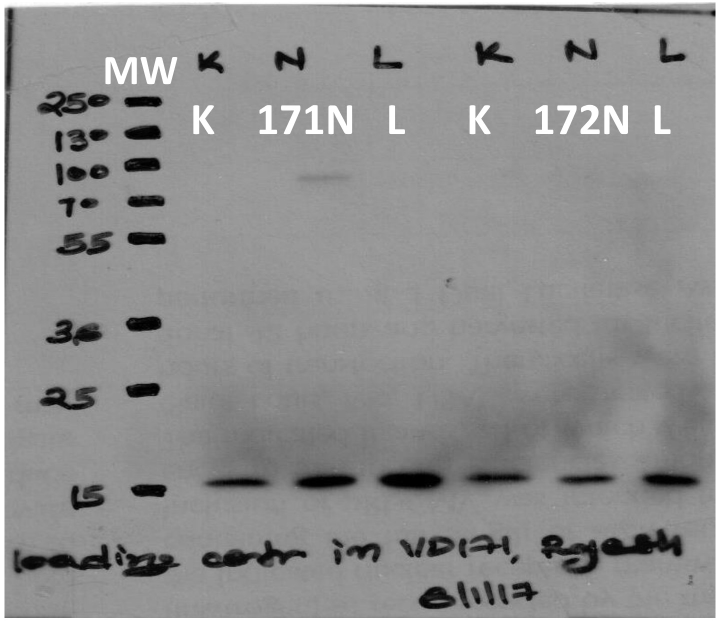

Cox-IV
15kDa

Full gel picture of Figure 5d (1) Cox-IV

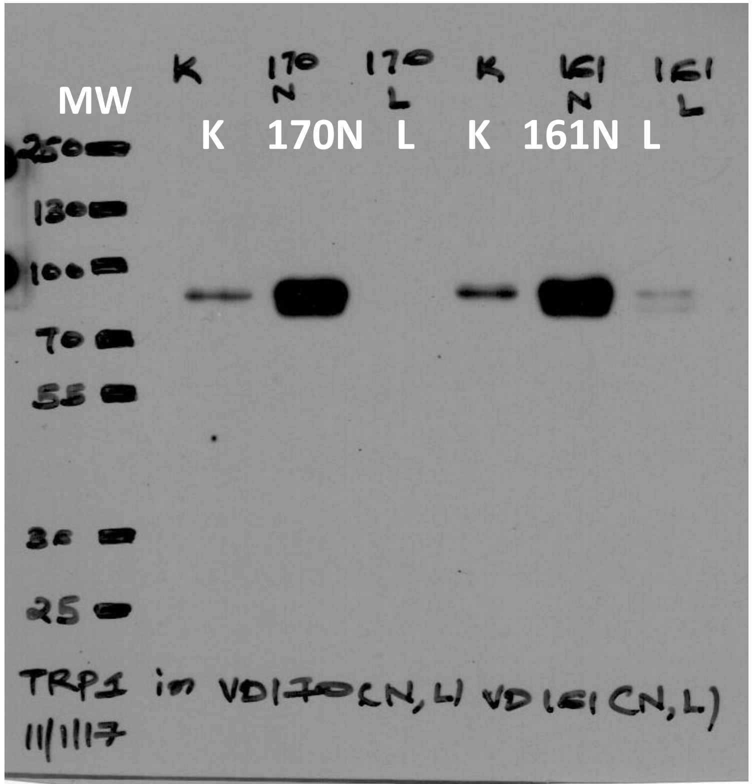

TRP1

Full gel picture of Figure 5d (2) TRP1

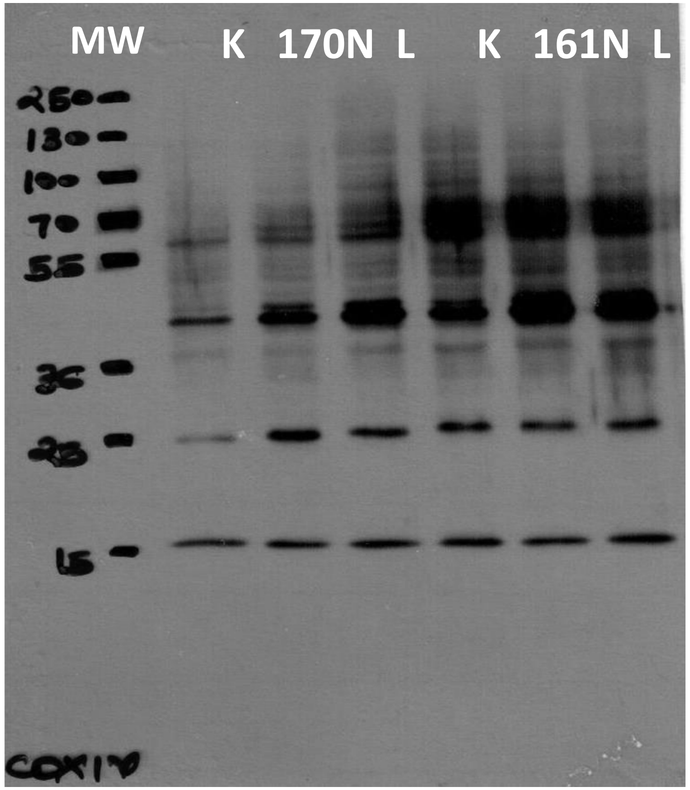

Cox-IV
15kDa

Supplementary figure 14

Full gel picture of Figure 5d (2) Cox-IV

Supplementary figure 14. Full gel pictures of figure 5d. Western blots for expression of TRP1 in four samples of Non lesional epidermis vs. lesional epidermis and normal human epidermal keratinocytes (678K) at protein level show downregulation of TRP1 in lesional epidermis compared to non-lesional epidermis and NHEKs. Multiple bands in the cox-iv gel for figure 4d (2) are due to many antibodies tested on the same gel.

Sample ID	miR-185	miR-525	miR-423	miR-518c	miR-202	miR-326	miR-518a
VD 13			1.38		0.66	1.47	
VD 15	0.35	0.19	1.36	0.17	0.08	0.22	1.62
VD 16	3.02	2.36	2.29	2.30	4.52	3.95	0.57
VD 18	1.66	0.74	3.50	1.20	1.25	1.48	
VD 20	6.33	4.89	10.16	6.87	13.09	10.83	1.22
VD 22	4.18	3.49	5.16	5.95	7.83	6.32	7.09
VD 24	18.69	19.45	8.23	14.12	16.81	9.01	13.48
VD 31	2.76	2.57	3.52	2.69	4.67	1.38	1.24
VD 33	2.60	2.80	5.97	2.31	2.92	3.12	0.96
VD 36		0.73	0.76	0.79		0.57	0.54
VD 39	0.84	0.74	2.11	0.99	0.75	0.88	0.63
VD 40	3.75	1.70	3.95	2.18		2.88	
VD 44	3.61	2.98	4.47	3.69	3.48	2.02	0.99
VD 46	2.44	2.53	4.42	2.05	2.90	1.74	
VD 47	1.66	6.48	1.21	2.38	1.82	1.79	1.71
VD 49	3.07	0.85	1.82	0.93		0.58	0.57
VD 54	1.20	2.69	9.71	2.10	2.49	1.59	1.29
VD 55	2.27	1.74	9.59	1.93	77.96	1.79	0.62

0 – 0.8 Down regulated

1.2 – 78 Upregulated

Not regulated

Supplementary figure 15. Expression (Fold change) of seven micro RNAs in the lesional skin compared to non-lesional skin. These micro RNAs induced down regulation of TRP1 in keratinocytes. Of the 18 subjects, 17 showed up regulation (shown in pink) of one or more of the seven miRNAs in the lesional skin compared with the non-lesional skin. One subject (VD36) did not show upregulation of any micro RNA of 375 tested. Blue colour depicts down regulation and black, no regulation.

	Predicted consequential pairing of target region (top) and miRNA (bottom)	Site type
Position 7486-7492 of TYRP1 3' UTR<br>hsa-miR-525-5p	5' ...AAAGCAGGUGAUGCU-UCUGGAAA...<br>3' UCUUUCACGUAGGGAGACCUC	7mer-A1
Position 4066-4072 of TYRP1 3' UTR<br>hsa-miR-518a-5p	5' ...AAGGAGGCAGGAGUUUUUGCAAA...<br>3' CUUUCCCGAAGGG--AAACGUC	7mer-A1
Position 3123-3130 of TYRP1 3' UTR<br>hsa-miR-202-3p	5' ...UGAACAACCUAGCUUAUACCUCA...<br>3' AAGGGUACGGGAUAUGGAGA	8mer

Supplementary Figure 16: Target Prediction for miRNAs: Target Prediction was performed for all selected miRNAs employing Target Scan algorithm wherein miRNA 202, miRNA 518a and miRNA 525 were PREDICTED to be directly targeting TRP1 mRNA.

.

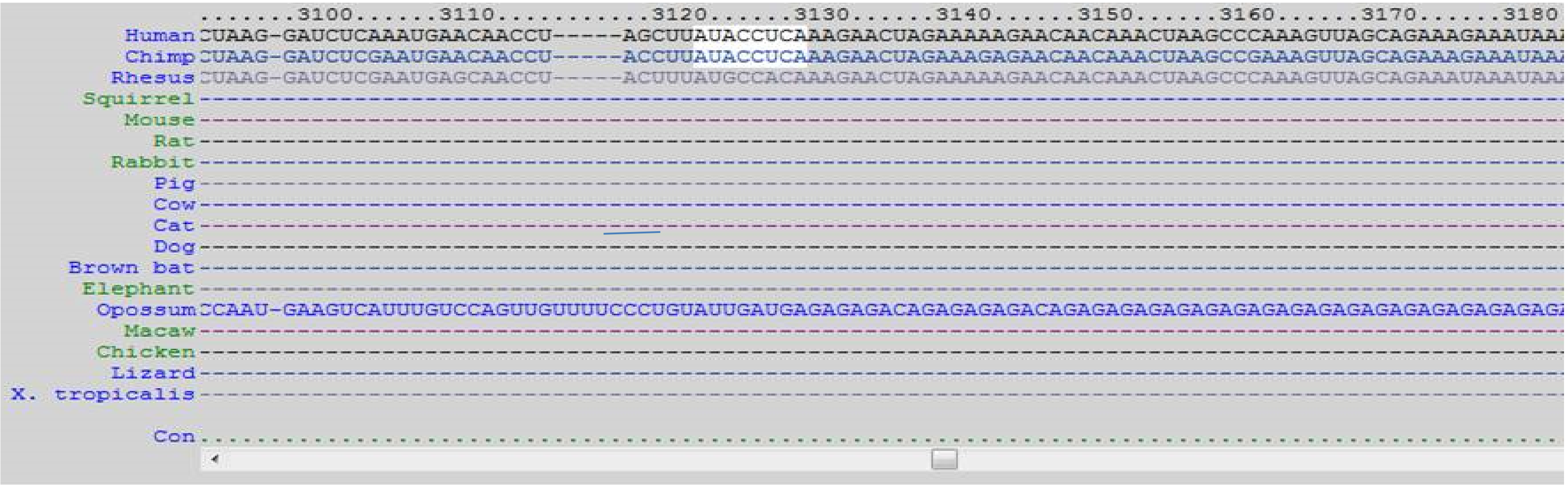

Poorly conserved

	Predicted consequential pairing of target region (top) and miRNA (bottom)	Site type	Context++ score	Context++ score percentile	Weighted context++ score	Conserved branch length	P_{CT}
Position 3123-3130 of TYRP1 3' UTR<br>hsa-miR-202-3p	5' ...UGAACAACCUAGCUUAUACCUCA...<br>||||||| 3' AAGGGUACGGGAUAUGGAGA	8mer	-0.19	85	0.00	0.017	N/A

Context++ score and features that contribute to the context++ score are evaluated as in Agarwal et al., 2015.

Conserved branch lengths and P_{CT} are evaluated as in Friedman et al., 2008, with an expanded 84-species alignment as described in Agarwal et al., 2015.

Supplementary figure 17. Screen shots of Target scan prediction for miR 202 shows that it's target sequences are poorly conserved with only Chimpanzee showing the conserved sequences (highlighted in white) in 3ÚTR of TRP1 gene

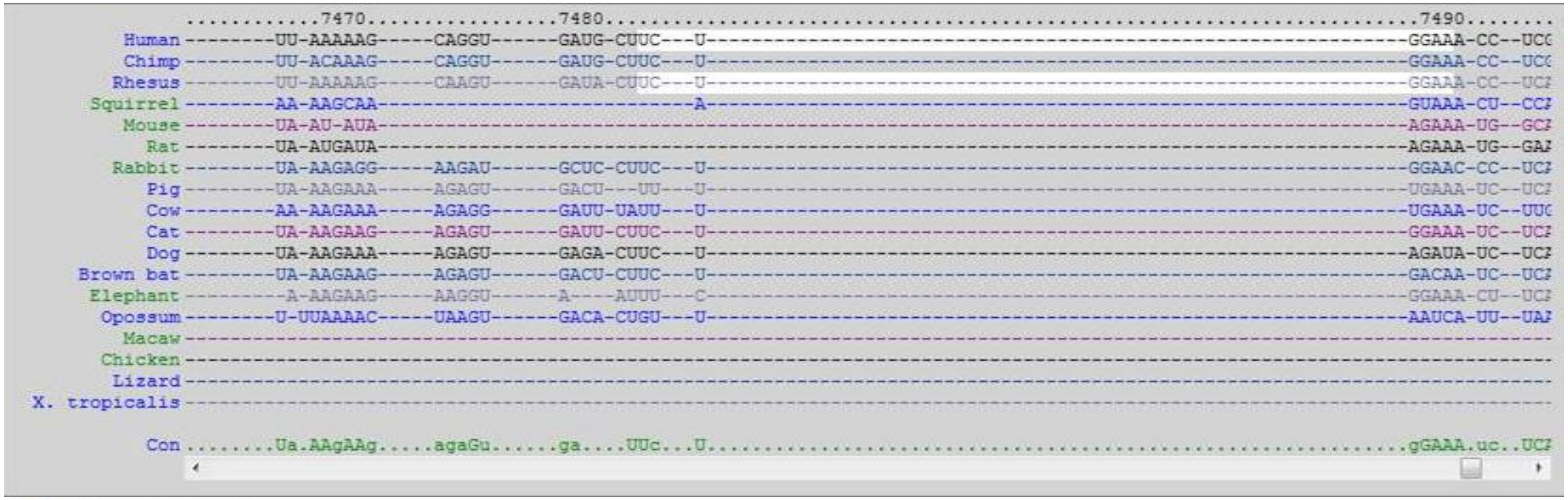

Poorly conserved

	Predicted consequential pairing of target region (top) and miRNA (bottom)	Site type	Context++ score	Context++ score percentile	Weighted context++ score	Conserved branch length	P_{CT}
Position 7486-7492 of TYRP1 3' UTR<br>hsa-miR-525-5p	5' ...AAAGCAGGUGAUGCU-UCUGGAAA...<br>||| |||||| 3' UCUUUCACGUAGGGAGACCUC	7mer-A1	-0.06	62	0.00	0.145	N/A
Position 7486-7492 of TYRP1 3' UTR<br>hsa-miR-520a-5p	5' ...AAAGCAGGUGAUGCUUCUGGAAA...<br>|||||| 3' UCUUUCAUGAAGGGAGACCUC	7mer-A1	-0.04	54	0.00	0.145	N/A

Context++ score and features that contribute to the context++ score are evaluated as in Agarwal et al., 2015.

Conserved branch lengths and P_{CT} are evaluated as in Friedman et al., 2008, with an expanded 84-species alignment as described in Agarwal et al., 2015.

Supplementary figure 18. Screen shots of Target scan prediction for miR 525-5p shows that its target sequences are poorly conserved with only Rhesus and chimpanzee showing the conserved sequences in 3ÚTR of TRP1 gene

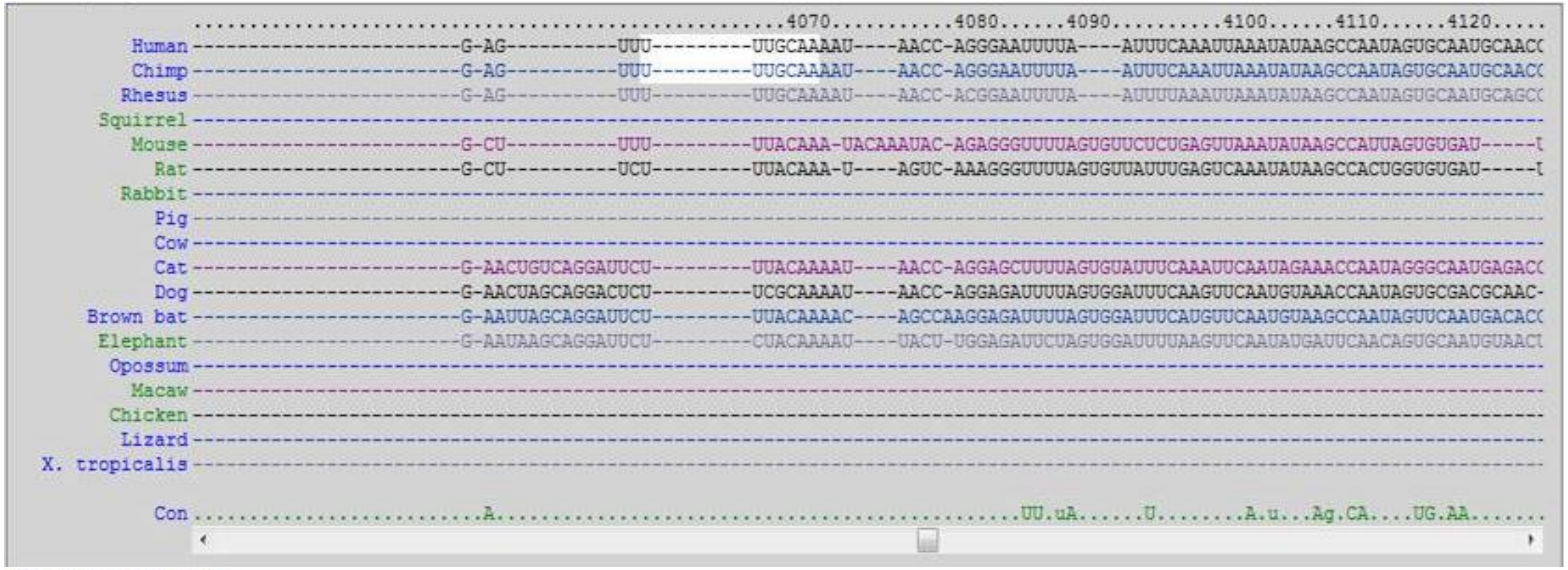

Poorly conserved

	Predicted consequential pairing of target region (top) and miRNA (bottom)	Site type	Context++ score	Context++ score percentile	Weighted context++ score	Conserved branch length	P_{CT}
Position 4066-4072 of TYRP1 3' UTR<br>hsa-miR-527	5' ...AAGGAGGCAGGAGUUUUUGCAAA...<br>||| |||||| 3' CUUUCCCGAAGGG--AAACGUC	7mer-A1	-0.03	44	0.00	0.017	N/A
Position 4066-4072 of TYRP1 3' UTR<br>hsa-miR-518a-5p	5' ...AAGGAGGCAGGAGUUUUUGCAAA...<br>||| |||||| 3' CUUUCCCGAAGGG--AAACGUC	7mer-A1	-0.03	44	0.00	0.017	N/A

Context++ score and features that contribute to the context++ score are evaluated as in Agarwal et al., 2015.

Conserved branch lengths and P_{CT} are evaluated as in Friedman et al., 2008, with an expanded 84-species alignment as described in Agarwal et al., 2015.

Supplementary figure 19. Screen shots of Target scan prediction for miR 518a shows that its target sequences are poorly conserved. Chimpanzee and Rhesus only are showing the conserved sequences in 3'UTR of TRP1 gene.

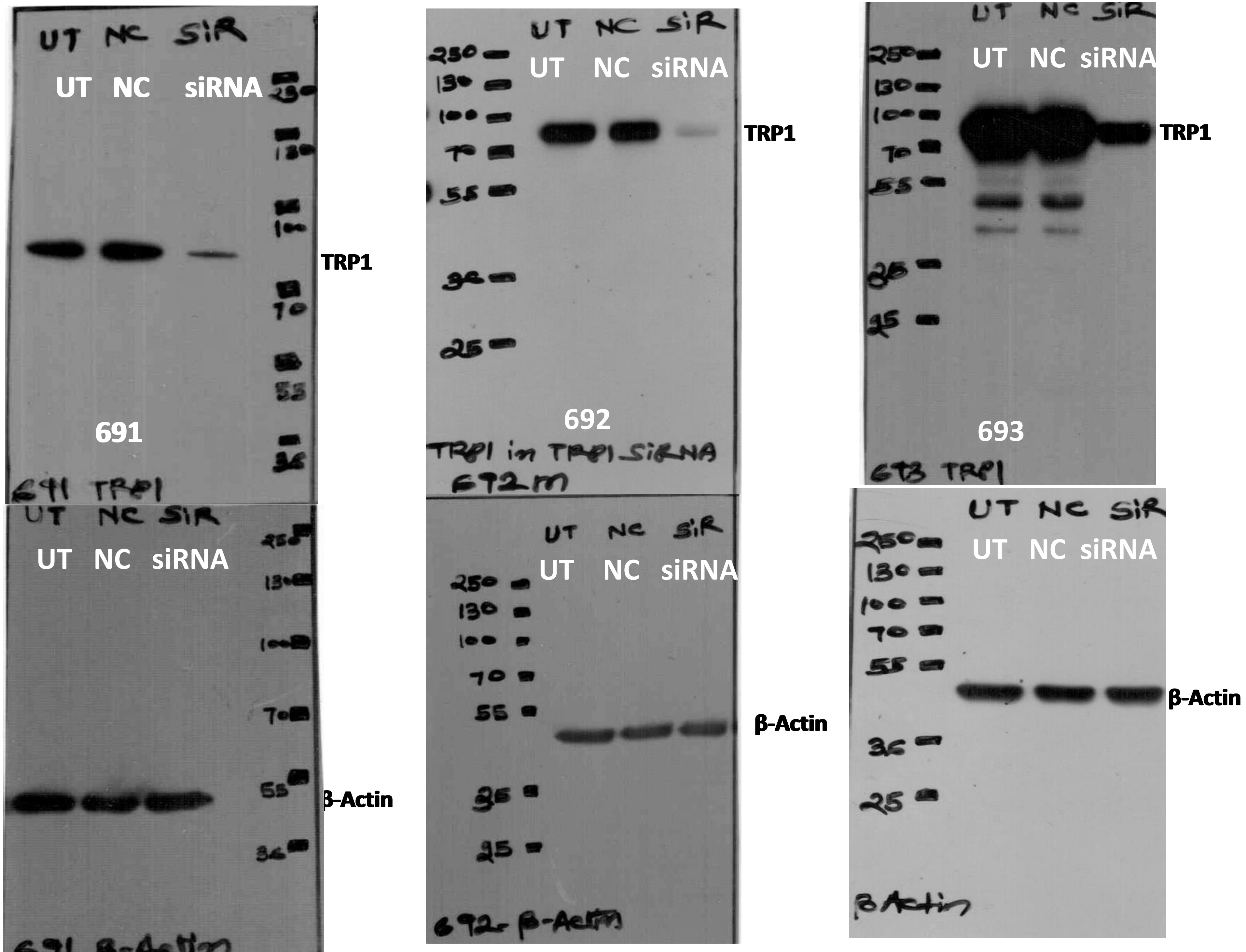
UT NC siRNA
TRP1
691
UT NC siRNA
TRP1
692
UT NC siRNA
TRP1
693
UT NC siRNA
β-Actin
UT NC siRNA
β-Actin
UT NC siRNA
β-Actin

Supplementary figure 20. Full gel pictures of figure 7 b showing expression of TRP1 and beta actin in siRNA transfected keratinocytes in 3 samples.

Supplementary Table 1. miRNA-dependent regulation of gene expression at protein level showing proteins potentially regulated by miR-326, miR-518a and miR-518c: data obtained from iTRAQ mass spectrometry.

S.No.	miR-326 Downregulated Proteins	miR-326 Upregulated Proteins	miR-518a Downregulated Proteins	miR-518a Upregulated Proteins	miR-518c Downregulated Proteins	miR-518c Upregulated Proteins
1	POLR2B	ACAT	RAP1GDS1	UBE2K	RAP1GDS1	GART
2	AK4	MICALL1	TNPO1	EIF4E	HNRNPH1	CCDC47
3	EIF4H	PHB	LARP1	CALD1	REXO2	PSMD5
4	USP10	PPA1	VDAC3	RPL30	KRT15	MTHFD1
5	ZC3HAV1	A2ML1	CAND1	CKAP4	SERPINB7	RPS3
6	PKP1	NCOAT	BAX	NUP62	CTPS1	DNAJB11
7	PPID	PROSC	FAM213A	KRT7	ZYX	GARS
8	COPB2	HMOX2	PPME1	FLOT2	ANP32	LGALS1
9	HIST1H2AA	PSAP	HIST1H1B	CAB39	EIF4H	EDF1
10	MTPN	ALB	ANP32	NRD1	ITGAV	EFTUD1
11	CTPS1	BCAT2	DNAJA2	PARP	ALDR	PSMD1
12	GDI1	KPNA3	NDE1	SQRDL	GAPVD1	CARS
13	CKAP5	UBAP2L	ADSS	COMMD9	MRPS36	GSTO1
14	HIBADH	XRCC5	DKFZp686A13 23	TRA1	NAT10	HSPA4L
15	PPM2C	KRT17	GLG1	AP2M1	NQO2	RPS26
16	DUT	PIP4K2C	MISP	IMPDH2	MTPN	RAB1A
17	EIF2A	LIS1	RABGAP1	RPL14	OCIAD1	ARPC3
18	CCT5	RPL12	FXR1	PGAM	EIF3M	OAT
19	DUSP3	MATB2	TYRP1	CDH1	SPRR1B	TXNDC5
20	PRKDC	U2AF1	ITPR3	SCFD1	TPM1	HSPA5
21	GPX1	RPS28	MYO18A	WARS	CKAP5	ARF6
22	HIST1H1B	ATAD3B	GSK3A	RPRC1	SYAP1	PHGDH
23	Sep-11	MACF1	RACK1	HNRNPL	PFDN4	KTN1
24	TYRP1	CDK3	FARSLA	KRT14	ABI1P1	ATL3

25	YWHAG	HADH	ZYX	PTMS	MISP	PRRC1
26	NUMB	RPL11	PRKCDBP	PDLIM5	HIST1H1B	SRP54
27	ABRACL	SLC25A5	SFXN1	OTUB1	PGRMC1	S100A9
28	ANP32	UBE2Z	FAM114A2	SFRS3	NACA	PDIA3
29	SNRPB	CKAP4	IASPP	KRT10	USP10	COPA
30	ITGAV	PSMD8	GDI1	RTN3	RPN2/PSMD1	POLR2B
31	ATP5	UBR4	SERPINB2	TBCA	MYPT1	FH
32	SPRR1B	EIF5B	SCARB2	PDLIM1	AK4	RPS7
33	KRT15	ACTN4	FACL3, ACSL3	ESYT1	LRRC47	K2C8
34	PSMD11	PDIA4	STRAP	COMT	TPR	CDC37
35	AARS	TAX1BP3	CLIP1	TUBB4B	PRKDC	SRPRB
36	BSG	MYO1B	EIF2AK2	TNKS1BP1	TXNRD1	GSTP1
37	MAPK1	RPL5	AK4	RPL9	HIST1H2AA	HEXB
38	AASDHPPT	CORO1C	ESD	ACTR1A	ADSS	COP9
39	FKBP2	DNAJB11	USP10	ADAR	SNRPB	PP
40	GMPS	NDRG1	PRKDC	TCTE1L	ARF3	COMMD9
41	MYO1E	PSMD7	THRAP3	IVL	NUTF2	EHD4
42	ESD	HN1	S100A2	GNB2	NFKB1	RPS21
43	PPME1	OPA1	ALDH7A1	PSMA1	RPL3	MYO6
44	PGRMC1	HSPA5	QRSL1P2	PSMD7	ZC3HAV1	KRT14
45	ADAR	VASP	STMN1	ALDOA	SF3B1/ SF3B2	IVL
46	MRPS36	EIF3A/ELF6	ALDR	RAB7A	ILK	RPS24
47	ROCK1	TPD52L2	NQO2	SFN	IPO5	RPS6
48	TXLNA	GNAI2	KRT15	UPP1	NT5E	PTBP1
49	TXNRD1	KRT9	MRPS31	KRT19	TYRP1	EIF5B
50	PTCD3	CDKN2A	SLC	TOP1	RRAS	DNPEP
51	LDLR	TCP1	ILK	CARS	ATL2	DSP
52	Sep-07	PFKL	UFL1	TXNDC5	CS	EHD2
53	DNM2	MTHFD1	SPRR1B	RPS21	ADK	RAP1B
54	CHCHD3	C7orf55	HEXB	UBE2N	NUP93	HNRNPA3
55	RAB14	MDH2	PC	UQCRC2	QRSL1P2	IMPDH2

56	TNPO1	ELF2S3	CAPRIN1	API5	RAD23BP2	SSB
57	CAPRIN1	TXNDC5	DNPEP	PHPT1	LMNB2	CKAP4
58	ARF3	SLK, FYN	IGF2BP3	NDRG1	RALA	TRIM28
59	HNRNPC	IMPA1	OCIAD2	RPL35	ALDH7A1	ACTN4
60	ALDH7A1	NOLC1	DNM2	S100A8	FAF2	PKP1
61	TCEB2	RPL17	NONO	PFKM	TAX1BP3	C7orf55
62	MRPS31	RPS2	CD9	OGDH	FAM114A2	ARMET
63	RDX	RPS7	HCG	EIF3C	SLC38A2	RPL8
64	CTSD	PSMA7	ARF3	HSPA4	PTCD3	UBE2K
65	VAT1	GSTP1	CAPNS1	SRSF1	GDI1	RPL14
66	STOML2	KIF5B	NFKB1	PRKRA	ROCK1	PRKCSH
67	SEC14L2	KRT19	S100A11	EVPL	BUB3	HNRNPAB
68	FAM114A2	ATP5O	NACA	RPL5	SARG	BCAP31
69	DYNLL2	MDH1	CCDC47	NMT1	TOM1L2	ACTA2
70	STMN1	KPNB1	ACOT7	FUBP	FAHD1	CDC42
71	SMBP	HIST1H2BL	S100A10	GLOD4	SEC31A	GIG18
72	STRAP	APP	CTSD	KRT16	PCBP2	PFDN6
73	MISP	BAIAP2	EIF2S2	KRT17	BAX	EEF1B2
74	GAPVD1	CSTB	CBR1	YWHAE	CAPRIN1	LIMA1
75	OCIAD2	UPP1	S100A6	TPD52	FBL	PP1A
76	TRAP1	SARG	H2AFY	EPRS	ATAD3B	SFN
77	EIF3G	FARSA	MYBBP1A	PHB	SNCA	MIF
78	ZMPSTE24	RAB3GAP1	LASP1	HNRPL	LYPLA2	RPL5
79	UGDH	RPS8	VAT1	NUTF2	PRDX6 (PRDX5)	JUP
80	EIF1AY	CAV1	PGRMC1	RIC8A	HNRNPC	CAPNS1
81	SERPINB9	PTMS	PAFAH1B1	HMGA1	RBBP4	PSMA1
82	IDI1	Sep-09	TRIP6	RNASEH1P	YWHAG	TCP1
83	NAT10	ETHE1	IKBIP	FSCN1	DUSP3	UQCRC2
84	PFDN4	CLTB	PDCD5	DARS	FAM213A	DFNA5
85	NANS	ANP32A	ABCD3	MACF1	NDUFA9	CSTB
86	SNU13	P5CS	GARS	PCBP1	Sep-07	FSCN1

87	MX1	ME1	ITGAV	SHMT	RTKN	KHSRP
88	NUP93	DARS	RBBP7	MRPL44	SORD	C2orf4
89	UGGT1	LUZP1	YARS	ACY1	PCBP1	LRPAP1
90	PIN1	KRT6A	ACOX1	PSME1	HADHB	PIP4K2C
91	GRPEL1	CLE	LDLR	PSMA7	GSK3A	STOML2
92	GSK3A	YWHAH	TMED9	G3BP	DECR1	IL1RA
93	APOBEC3A	DNPEP	EIF3G	SERA	PAFAH1B1	OGDH
94	LYPLA2	RARS	ZFP36L1	HSP70	SNRPC	ARPC1B
95	ATIC	RAB1A	RPL13	SNRPB	RPL23	TXNDC17
96	EDF1	MAOA	TOM1L2	LAMB3	AKAP12	GLS,GLS2
97	SEC31A	SCP2	AKAP12	HIST1H1E	VPS4B	RPN2
98	SCARB2	ACOX1	DUT	TUBA3C	ZFP36L1	LUZP1
99	IKBIP	SFN	NADH	LGALS7	EIF5	PSMA2
100	CD9	ABCF2	MAPK1	PKP3	PAPSS1	PSMA5
101	SF3B1	IVL	VPS26A	RPL27	PSMD7	HNRPUL1
102	IGF2BP2	EFTUD2	CDK3	RANBP1	MYO1E	C1orf116
103	RACK1	EIF1	BUB3	RPL11	RACK1	TES
104	FAHD1	PCYT1A	DLD	CKAP5	CD2AP	TAGLN2
105	PRKCDBP	LUC7L2	PAPSS1	CRABP2	ACOX1	PTMS
106	AK1	SFPQ	SNX9	RPS27A	S100A6	LAD1
107	PCBP1	TTLL12	SMD3	RAC2	ATAD3A	TPD52L2
108	EIF3J	NQO2	DUSP3	TM9SF2	VPS29	ATP6V1B2
109	NDUFB10	CBX3	PYG	GSDMA	TCEB2	NIPSNAP1
110	RPL23	CIAPIN1	EIF3B	PCSK4	RPS9	PPP2CA
111	LRRC47	RPL30	NANS	MTCH2	PMPCA	DCTN
112	ITGA6	S100A9	IPO5	BAF	CD59	PKM
113	CLIP1	FKBP1A	AK1	ACTN4	ALB	NUDT5
114	ATP6V1E1	RPL9	CD44	CLIC3	KPNA6	AP1B1
115	GLG1	CLIC3	ATXN2L	RPL38	TNPO1	PCYT1A
116	FLII	FUBP3	ATP6V1A	RHOC	CSNK2A2	SF3B2
117	PTPN11	PREP	LMNB2	VPS4B	RPL13	KRT8

118	CCDC47	NSDHL	NAT10	C1QBP	MARS	SRRM1
119	MRPS27	FAM162A	BICD2	NDUFB10	DEAD/DEAH	ACY1
120	RUVBL2	SRI	MTHFD1	NCOAT	HPRT1	IGF2BP3
121	PFKM	TUBA3C	RDX	CSTB	CDA	USO1
122	REXO2	ERO1A	ZMPSTE24	EIF3L	GRPEL1	TTLL12
123	CPNE3	LAMB3	NUP93	PCBP2	PSAP	EIF6
124	ITPA	MAP2K1	ATIC	UQCRFS1	RPLP2	CMPK1
125	VPS4B	OGDH	LRRFIP1	ERP44	S100A2	BLVRA
126	CSN5	SERPINB8	ATP5	LIMA1	RHOA	ME1
127	CAND1	IL1RA	MAPRE1	NIT2	G3BP1	CALD1
128	CALD1	SRSF7	VASP	ERO1A	HMGB3	RPS14
129	HNRPUL1	CRYZ	CDC42	DGKA	TRAP1	CLIC3
130	TECR	GPD2	COPB2	NUDT5	PACSIN2	DYNC1LI1
131	S100A6	SHMT	SNU13	SYNC	AK2	RABGAP1
132	NRD1	HIST1H4H	RBBP4	SF3B3	NOMO1	KRT6A
133	NFKB1	KRT2	CACYBP	AKR1A1	Sep-11	ALDH9A1
134	EEA1	DNAJA2	LNPEP, PLAA	DSP	MAPK1	SERPINB6
135	ARL8B	HNRNPA3	KLC2	Sep-07	STMN1	SYNC
136	G3BP1	NXN	RTKN	SNCA	PEBP1	P5CS
137	FKBP3	CAPZA1	NELFB	EPPK1	TARS	ADH5
138	PRDX4	RAB21	ATAD3A	KRT6A	RUVBL2	A2ML1
139	RPL3	OCIAD1	DYNLL1	PSMD6	AHSA1	PTRF
140	VIM	PTGR1	CHCHD3	DDB1	GLG1	GSDMA
141	TRIP10	FTH1	CYB5R3	PRDX4	ACOT7	PSMA7
142	ALDR	AIM1	SYK	FH	APOBEC3A,	SNRP70
143	TPR	RANBP1	HDLBP	LRPAP1	VAMP3	EVPL
144	UBE2D3	MTAP	RALA	ANXA	DYNLL1	NAMPTP1
145	FACL3, ACSL3	FDFT1	Sep-11	EIF3F	BCAT2	TUBB2B
146	PPP2R2A	EEF1A	ALYREF	MTPN	BSG	TOP1
147	LRRFIP1	SNRPD2	FAU	LAD1	PSMB2	PYCARD
148	RTKN	EIF2AK2	NME1-NME2	LEREPO4	GSN	RPL18A

149	Hcg	KRT5	TCEB2	SERPINB6	COPZ1	COMT
150	STAT1	SUI1	COPZ1	LAMA3/4	ARL8B	ATXN2L
151	UFL1	UBE2N	CHMP4B	HNRNPA3	HSD17B4	VPS35
152	AP2M1	HSGP25L2G	OSTF1	PPID	CLIC	SF3B3
153	SORD	COPS7B	SRP14	EIF5B	PTPN11	GOLGA3
154	ADSS	MYO1C	PSMC3	RAB2A	TM9SF2	EIF1
155	SRM	RPL	EIF5A	ALDH9A1	ATOX1	ATP5H
156	COTL1	ITGB6	PTCD3	CAD	RPL23,RLP17	RAC2
157	CD59	ITPR3	FDPS	SLK, FYN	KIF5B	COPG1
158	ATP6V1A	PPIF	DEK	BCAP31	NANS	SDHAP2
159	CDA	LNPEP, PLAA	PAI2	CKMT1A	EIF3G	NONO
160	VDAC3	RPS14	SERPINB7	HNRNPH1	MAPK3	THRAP3
161	TSG101	NEAT1	EEF1A1	RANGAP1	DKFZp686A13 23	ECHS1
162	CNN3	KPNA4	NEDD8-MDP1	VPS35	HK1	PROSC
163	PTBP1	GRHPR	NDUFA9	ATL3	TPM4	KRT7
164	SF3B2	TPD52	CFL2	RPS23	ABCD3	IPO7
165	RTN4	SYNC	PEBP1	A4	MRPS31	TPD52
166	CHP1	NUTF2	NDUFA10	VPS4A	UFL1	LTA4H
167	PDCD5	NONO	EIF4H	IDH2	NMT1	DDX6
168	ITGA2	PLAP	ACLY	PYCARD	CNN2	FUBP3
169	RTCB	ATP6V1G1	VAMP3	PSMD5	SNRPD3	FDFT1
170	SMS	RNPEP	APRT	ME1	CLIP1	MTAP
171	EIF3K	IDH2	NOMO1	ACTA2	RDX	ATP5D
172	CTNND1	OAT	OLA1	YWHAH	S100A10	API5
173	GNB1	DYNC1LI1	PACSIN2	SCP2	DNM2	GRHPR
174	HSD17B4	SDHAP2	THIL	ANXA6	EIF3B	TACSTD2
175	PSMD6	CAPNS1	HNRNPF	SNRP70	NRD1	ETFA
176	RRAS	VPS35	ITGB1	RPL3	CACYBP	PPL
177	STK24	KRT16	ATL2	FKBP2	ANXA5	DDX21
178	CAPN2	METAP1	TARS	SERPINB9	S100A11	ACTR1A

179	RALA	LAMA3/4	HNRNPC	FTH1	CTSD	MPRIP
180	YARS	LAMC2	SSB	PSMA6	CAPN2	C1QBP
181	IFI16	NDUFA9	RPS13	RAB1A	ARL1	LARS
182	NACA	BUB3	RHOA	CASP14	VARS	CRABP2
183	RPN2,PSMD1	EIF6	SEC23A	CAPN1	OSTF1	ETHE1
184	IDH1	ARF6	ZC3HAV1	PAK1, PKN1	EFTUD2	SERPINB8
185	HP	PSMA6	NAA10	NAPG	CPOX	KLC3
186	HSPA4L	SF3B3	MX1	KTN1	BAF	Sep-09
187	PSMA2	S100A8	ATP6V1B2	TARDBP	ATP1	NAMPT
188	PKP3	LMNB2	HK1	PTGR1	SARS	PSMA6
189	PPP1R7	ACY1	NDUFS3	KIF5B	TSN	UQCRFS1
190	ABI1P1	GLOD4	PKP1	RAB5C	STRAP	CAPN1
191	C2orf4	NPM1	PACSIN3	APP	PSMC1	HIST1H2BL
192	SNCA	FAF2	IFI16	SFPQ	PSMC3	PPIF
193	HADHB	PEA15	TXLNA	TPM1	LDLR	TCTE1L
194	RPL23/17	TXNDC12	PC4	KPNA4	GLO1	KRT17
195	IARS2	SRP54	ABCF2	NEAT1	CPNE3	EIF2B1
196	EIF3B	PSMD5	FAM3C	GRHPR	HNRNPF	LAMA3/4
197	LASP1	DDX21	PREP	C2orf4	NUMB	ESD
198	PAK2	RIC8A	PTBP1	PSMC4	TMSB10	MTCH2
199	CD44	COMMD9	RPLP2	BAG3	MICALL1	NELFB
200	HNRPDL	CRABP2	ARHGDIA	SNRPD2	PPM2C	UBE2N
201	DYNLL1	GRSF1	ATP6V1E1	HSGP25L2G	SERPINB2	PAI2
202	H2AFJ	DDX1	GPX1	OCIAD1	GNB1	YWHAH
203	MYH10	HNRNPH1	FLII	RAP1B	IASPP	CIAPIN1
204	PLEK2	ERF1	ADK	COL17A1	CBR1	PSMD14
205	PYGL	CSRP1	RPS15	NPM1	FKBP3	FLOT2
206	NUDT5	SNX6	COPA	FARSA	CORO1C	NXN
207	PYG	IGF2BP3	LGALS3	TUBA1A	RHOC	ANP32A
208	TROVE2	RAC2	NPEPPS	C17orf25	ELF2S3	RPL26
209	NUMA1	HSP90AB3P	TSN	P5CS	RAB3GAP1	MGST1

210	ACAT1	HIST1H2AB	DDX6	LMAN2	PSMC4	CDH1
211	CS	S100A13	PFN1	PPP2CA	RBBP7	LMAN2
212	SH3GLB1	ERP44	ABRACL	KRT9	LRRFIP1	RPS23
213	TWF2	FABP5	VDAC1	ZNF185	YWHAQ	NEAT1
214	NAP1L1, NAPSB	NAMPT	GIG18	IL1RA	LASP1	PFKL
215	PFDN6	TIM14	AK2	UGDH	H2AFJ	SFPQ
216	PABPC4	TP53I3	ALCAM	RPL28	MYO18A	ESYT1
217	PSMC3	VBP1	COP9	WASF2	CNDP2	LAMB3
218	ANXA4	PHP14	PSMB2	MRPS36	TXLNA	PEA15
219	RBBP7	GNB2	PA2G4	SNX6	IGF2BP2	EFHD2
220	MAPK3	MGST1	ACAD	KRT5	PYG	CBX3
221	CNDP2	APRT	PGK1	ETHE1	GFPT1	NPM1
222	DHX9	PSMD1	ARPC2	PPP1R12A	DGKA	DDX1
223	ILK	MYPT1	RPL7	S100A13	NDUFA10	HIST1H4H
224	NHP2L1	LMAN2	HMGB3	PSMD1	VAT1	HNRNPL
225	HNRNPF	NELFB	HARS	CSRP1	MX1	PRDX3
226	NOMO1	K2C8	SNRPC	RPS14	RTN4	KRT16
227	WARS	PPL	KPNA6	RAB21	TRIP6	LAMC2
228	TSNAX	PSMD4	SSBP1	SBSN	HSP70	HSGP25L2G
229	RALB	DDX20	DDX39B	GMPS	VAPB	RPL9
230	PON2	RPL26	UGGT1	PEA15	P4HA1	FABP5
231	FH	CAPN1	PSME3	RPS24	SYK	EIF2A
232	TALDO1P1	UBE2K	CNN2	MPRIP	NAGK	NSUN2
233	RPS3	PAK1, PKN1	AIM1	FABP5	USP5	GPD2
234	IPO5	CDH1	ANXA5	S100A9	TBCA	IDH2
235	NADH	EIF5	EIF1	DYNC1LI1	SRSF7	MYO1B
236	ABCD3	PRDX3	VIM	PPIF	SERA	RAB5C
237	TPM1	NIPSNAP1	TRAPPC3	RPS28	PAFAH1B2	STAM1
238	THRAP3	ZNF185	RPL23A	EFHD2	RPL30	YWHAB
239	NUP62	NAMPTP1	SF3A2	TPD52L2	DDX39B	PSME3
240	S100A2	HNRPR	MARS	HIST1H2AB	FACL3, ACSL3	PPME1

241	SYK	PRPF19	NCKAP1	TACSTD2	UQCRC1	PPA2
242	SRRM1	A4	VARS	CAPZA1	HDGF	PRPF19
243	TIMM50	NOL5A	TMSB10	DDX1	VASP	OPA1
244	PCBP2	EFHD2	RPL18	LAMC2	PAK2	TUBA3C
245	DNM1L	KLC3	COPS6	MYO1C	VDAC1	ITPR3
246	ATP1	PDAP1	H2AFJ	CIAPIN1	GLOD4	KRT5
247	RPL23A	PC	FAF2	EIF2A	SNU13	CD44
248	NEDD8-MDP1	RPRC1	COX5A	SNRPD3	PFKM	ZNF185
249	COPG1	ACTR1A	KRT1	PES1	ATP6V1A	SNRPD2
250	JUP	SFXN1	TRAP1	LYPLA2	UGDH	HSP90AB3P
251	ANXA6	PFDN2	UBE2L3	PFDN2	VPS26A	DNAJA2
252	EIF3D	HN1L	GNB1	RPL8	ANXA11	DDB1
253	VPS29	SBSN	STK24	YBX3	RAB14	PAK1, PKN1
254	COX5A	FXR1	ITGB4	SLC38A2	TNKS1BP1	DNPH1
255	SF3A1	SAM50	CALR	PRPF19	ITGA6	CRYZ
256	MOGS	PXN,PXDN	RAB3GAP1	CRYZ	PALLD	HNRPR
257	TMSB10	YWHAB	PLEK2	MGST1	PRKCDBP	KRT1
258	VPS26A	CASP14	HSPE1	NIPSNAP1	COX5A	KRT9
259	YBX3	RPS9	IQGAP1	EIF6	HARS	PRDX4
260	DNPH1	CALM3	CHP1	PDHB	AHCY	RAB21
261	GSN	ARHGEF1	RUVBL1	SERPINB8	RPS13	ERP44
262	DEAD/DEAH	LAMTOR1	TWF2	PLAP	SUI1	SH3GLB2
263	PMPCA	LARP1	ACSF2	CSN5	DLD	NAPG
264	ADK		JUP	ITPA	PACSIN3	BAIAP2
265	TARS		CANX	FDFT1	CD9	CSRP1
266	PAFAH1B2		RPS12, MRPS12	AARS	ITGA3	CDK3
267	SLC3A2		RAB10	MYO1B		ABRACL
268	DECR1		DNCL1	DNAJC7		PPID
269	NIT2		SLC3A2	FBL		TP53I3
270	PALLD		EEA1	PPL		HIST1H2AB
271	PSME3		KRT2	EIF5		FXR1

272	LRPPRC		APOBEC3A	ITGB6		GNB2
273	TUFM		CNN3	ATP6V1G1		PSMD4
274	G6PD		DDX17	LRRC47		LNPEP
275	EHD1		HPRT1	FUBP3		CAPZA1
276	HMGB3		ATP5L	ARF6		YBX3
277	NAGK		MYH10	PABPC4		ERF1
278	RAD23BP2		FKBP9,FKBP1B	HIST2H2BD		SBSN
279	NPTN		MVP	COPG1		ITGA2
280	ANXA5		PALLD	PRDX3		SMBP
281	PAICS		HADHB	DNPH1		PFDN2
282	XPO1		ATP5A1	ERF1		PDAP1
283	RAB10		FKBP3	COTL1		EEF1A
284	NPEPPS		HSPH1	IGF2BP2		A4
285	ARL1		GRPEL1	EHD4		PXN,PXDN
286	PCMT1		XPO2	TXNRD1		SNX6
287	SLC		NOLC1	MATB2		RPRC1
288	S100A10		TMEM43	SF3B1/B2		CASP14
289	TNKS1BP1		RPS16	KLC3		VBP1
290	PPFIBP1		CD59	TSPAN1		DDX20
291	TKTL		RPL23,RLP17	RPL26		LAMTOR1
292	GART		RRBP1	DFNA5		SFXN1
293	CMPK		H2AFV	OAT		CALM3
294	SEC23A		PSMD11	NAMPTP1		SAM50
295	CD2AP		SERPINH1	POLR2B		LARP1
296	FTL		RTN4	NAMPT		HN1L
297	RPS21		LDHA	UBR4		
298	EVPL		SARS	NOL5A		
299	CLINT1		PAICS	GPD2		
300			PCMT1	RPS9		
301			YWHAQ	SMBP		
302			ACSL1	EEF1A		

303			DNAJB11	YBX1		
304			RPS11	SAM50		
305			GPI,GNPDA1	VBP1		
306			PRDX6 (PRDX5)	HP		
307			ITGA2	PDAP1		
308			GLO1	PXN,PXDN		
309				ARHGEF1		
310				ALB		
311				HN1L		
312				CALM3		
313				LAMTOR1		

Supplementary Table 2. miRNA-dependent regulation of gene expression at protein level showing proteins potentially regulated by miR-185, miR-202 and miR-525: data obtained from iTRAQ mass spectrometry

S.No.	miR-185 Downregulated Protein	miR-185 Upregulated Protein	miR-202 Downregulated Protein	miR-202 Upregulated Protein	miR-525 Downregulated Protein	miR-525 Upregulated Protein
1	GCP60	PPP2R2A	GCP60	RPL35	B4E1T1	Q59G24
2	TIMMDC1	COX5A	GTF2F1	MYG1	GCLM	MRPS36
3	ATXN10	VPS4B	GNB2	DPP3	KRT19	Sep-09
4	PLOD1	RPL7A	B4E1T1	SERPINH1	TIMMDC1	APEX1
5	OAS2	HNRPU	PSMB5	RPS8	CD44	SFXN1
6	PRKAR2A	CDV3	B2R7W4	KRT17	ITGA3	ACTN4
7	FEN1	LAMA3	LMNB1	Q53F64	TCEB1	hCG
8	PPP2CA	EIF2S1	GMPR2	ERP44	TMPO	B2R608
9	TIGAR	AHNAK	TYRP1	SNRPD1	AACS	PDCD10
10	ACOT13	EIF3B	TIGAR	STK24	FEN1	AKR7A2
11	HLA-B	RAC1	B3KWV6	PRDX4	RAB35	GNS
12	ITGA3	A8K9K6	SOD2	ITGA3	ACACA	SOD2
13	LMNB1	EZR	PI4K2A	BOLA2	API5	ARPC2
14	YWHAH	TSTA3	GSS	PFD5	LMNB1	ZNF185
15	GMPR2	B2R5U3	TPP2	YWHAQ	TYRP1	SFN
16	TPR	HNRPL	EIF3I	A8K897	Q53FN0	B4DTP3
17	RALB	TOM40	B2RB07	RPS7	ANXA8L2	EIF3D
18	EIF3I	Q53GF0	OAS2	SERA	ACOT13	Q8NCF7
19	TMX1	ANXA1	FEN1	GRP78	ABCF1	IVL
20	PFAS	LYRIC	PTPN12	ACTH/POMC	RBBP4	B7Z6Y3

21	BAG6	AHNAK2	Q53GR7	SYK	PLEK2	EIF4B
22	RAB35	CKAP4	ABCF1	ARCN1	HIST1H1B	COPG1
23	HLA-A	C17orf25	Q8NCF7	NMT1	KRT2	DYNLL2
24	B2RB07	LPP	DTYMK	LRPPRC	MUT	GSS
25	CD44	ECHS1	ATL3	SDF2	Q53FC3	UAP1
26	SCAMP3	SFN	ATL2	MX1	SRPR	PSMD12
27	TYRP1	UBE2D2	Q2TTR7	DIAPH1	BAG6	GCP60
28	AKAP12	SDF2	PPP2CA	S100A6	STK3	FAM49B
29	KRT2	NSDHL	M6PR	KIAA1967	SLIRP	SERA
30	Q53FG5	LETM1	INF2	NIBAN	ARHGEF2	PPM1G
31	CIRBP	DYNC1LI2	EIF6	RRAS	TPBG	COX6B1
32	Q2TTR7	ERP44	B2R6M5	RPL7A	GNB2	AHNAK2
33	RAB13	PC	PRPS2	CYCS	NENF	MOB1A
34	SERPINB6	GPX1	ACACA	IRF6	PRKAR2A	RPS26
35	DNAJC7	MYO1E	PFKL	HSPH1	Q53GR7	TUBA4A
36	UBXN1	RPL9	DNAJC9	DHX9	B4DHQ3	ECH1
37	Q53GE2	SRSF5	YWHAH	CASP1	AKAP12	ARCN1
38	PDLIM7	CLNS1A	TPBG	UCHL3	B3KY60	HLA-B
39	TPBG	PSMD2	ARSA	P4HA1	PP1B	ZO2
40	SYPL1	FH	GSTM1	EFHD2	ATXN10	C17orf25
41	MAT2A	Q69YG1	CD44	PACSIN3	LPP	RPL3
42	ABCF1	RPS20	GIPC1	SRP68	TPR	DLD
43	RBBP4	DLD	MRPS36	FYCO1	GSTM1	EIF2S1
44	EIF4H	DHX9	TIMMDC1	KRT18	SERPINB6	HNRPL
45	TSPO	TNPO1	H13	NAPRT1	YWHAH	SH3BGRL

46	IGF2BP3	RPS26	ALDH9A1	EIF3K	GORASP2	B2R5U3
47	HNRNPA0	IMPDH2	A8K168	VASP	Q53G17	Q6NVC0
48	NCLN	GOLGB1	CARS	TXNDC5	PPP2CA	BPNT1
49	RPL28	DCD	B2RAY1	Q6NVC0	PON2	STOML2
50	LMOD1	CAST	KRT19	METAP1	Q2TTR7	HSP90AB3P
51	Q8N995	CBX3	NDUFS1	Q53HN4	ADSS	AIMP1
52	LIMK2	P4HA1	SERPINB6	NEDD8	Q59EL4	GTF2F1
53	SEC24C	ZO2/TJP2	CLIP1	CAD	Q53GJ8	A2ML1
54	GCLM	ERF1	RPS6KA1	HNRNPF	Q8NCW8	RCC1
55	PROSC	RPS25	ATP1B3	DNAJA1	GIPC1	S100A8
56	PHP14	CCDC58	CIRBP	LIS1	Q59EK3	RPL9
57	PRMT5	TBCA	CKAP5	HSD17B12	SNCA	EIF6
58	MYBBP1A	FKBP4	ERC1	DNPH1	ARSA	DIAPH1
59	Q8NCF7	Q53GX4	AACS	B4DRS6	P5CS	Q7Z612
60	API5	PTRF	B2RAH5	RPL9	Q8N995	SAM50
61	ACACA	SERA	B2R5N2	CALU	LYPLA1	RPS25
62	NUP107	ACTN1	B3KS50	A8K878	M6PR	B4DRS6
63	SUCLG2	Q53FN0	Q8N8J5	HIST2H2AA3	Q8N8J5	GRP78
64	PKM	HNRNPUL2	AIFM1	CHCHD6	B4DF00	A8KA84
65	CAZA1	PSMC2	TMPO	PSMC2	B3KWV6	NAA50
66	B4DEA6	SLC25A10	LARS	BCL2L2	B2R7W4	PGRMC1
67	ACY1	ERC1	UBXN1	BAG3	EIF3J	GARS
68	TMPO	DYNLL2	Q8N995	FTL	RPL28	SH3GLB1
69	MRPS36	PSMA5	TPD52	PRDX3	Q59EH3	PSMD2
70	PARP1	SLC3A2	CAPZA1	Q53FV3	ATL2	RAC1

71	PRDX4	MCTS1	SNCA	EIF2S1	RAB13	A8K529
72	ALDH9A1	BPNT1	ADH5	HNRNPC	MVD	HSPA4L
73	DDX21	GNS	TRMT112	UBE2K	KRT10	RPS27L
74	USP5	DDX17	B3KR50	SCRN1	TMX1	CCDC47
75	LONP1	Q9UNM1	ANP32A	PGRMC1	SF3B2	B2R7T8
76	DTYMK	SRM	BAIAP2	A4GYY8	LIMK2	CIRBP
77	MUT	SUI1	Q59EL4	EVPL	KRT1	BAIAP2
78	Q59EK3	DUT	ITGAV	CSRP2	Q53F62	TRIM29
79	HADH2	RPL8	Q59GB4	ABCF2	AHSA1	HIST2H2BF
80	GFPT1	PDIA4	FAM129B	SERBP1	RHOA	CAST
81	IKBIP	DLST	VPS35	TPM1	NUP107	PTPN11
82	Hcg/HTC2	STOML2	PLCB3	SFPQ	MPRIP	CTNNB1
83	GSS	IVL	IGF2BP3	VAMP3	ERF1	HSD17B4
84	PDAP1	ACTN4	UQCRFS1	LYPLA2	NDUFS1	PDLIM5
85	Q54A51	HNRNPF	KRT10	REEP5	Q59GB4	TCEB2
86	RBP2	PTGS2	COPE	LRRFIP1	AIFM1	GLRX3
87	Q8N7G1	Q8N8J5	SLC25A10	FAF2	EIF3I	LARS
88	Q53F64	Q7Z612	CCDC58	TAF15	ATP1B3	YBX3
89	SKP1	SNRPD3	CNN3	FUS	TPP2	FYCO1
90	PI4K2A	ATL2	VPS26A	PIP4K2C	PTPN12	YWHAQ
91	ACADVL	Sep-09	TACSTD2	GNS	KPNA1	BICD2
92	EIF3K	COPB2	SKP1	B7Z592	B7Z4B7	B4DEA6
93	Q53GW1	TXNDC12	TRIP6	HIST2H2BF	RCN2	GCN1L
94	RANGAP1	CAPRIN1	NUDT5	RPS25	EIF5B	PPA1
95	PSAP	RAP1B	DNAJC3	RAB10	PPP5C	PXN

96	RPS28	PSMD14	UBC12	FKBP2	A8K9B9	Q53F64
97	EML2	PSMD3	MYH4	HADH	RPS16	DNAJB1
98	UNC45A	MRPL4	WARS	FTH1	A8K168	B4DGF6
99	FIS1	HSPH1	HADHB	A8K237	AP1B1	XPO1
100	RAB5B	KPNA1	B3KRC6	CRKL	PFDN4	Q59E99
101	INF2	GPD2	COPG1	VDAC2	DDX21	A8KAK1
102	SOD2	ANXA8L2	RPL26	RAC2	PSAP	RPL35
103	HNRNPH1	A8K529	LYRIC	Q53FT8	DTYMK	A8K878
104	NCKAP1	PXN	CFL2	RPN2	ERC1	IPO7
105	RPS9	S100A14	NCKAP1	TPD52L2	B2R5N2	MYG1
106	IKIP	COX6B1	FUBP1	ECH1	RPLP2	RPS15
107	RPLP2	CDD	PPP1CA	B2R608	GFPT1	CLTB
108	TPP2	KRT18	A8K3W4	HLA-A	HNRNPA0	CAD
109	Q7LD69	B7Z8X5	P5CS	PDCD10	NSDHL	ALYREF
110	A8K9B9	TRADD	Q59EK6	S100A16	PRKDC	LAMC2
111	SRPR	H13	ATP5J	HSPA4L	RAB5B	SRSF5
112	CDK5RAP3	YWHAG	GNAI2	ATOX1	DUT	SQSTM1
113	TWF2	FAU	HN1	GLRX3	NUCB2	AKAP2
114	ITGAV	MOB1A	EPPK1	SNRNP200	HNRNPH2	AK2
115	GIPC1	PDLIM5	RPL28	THIL	B4DK16	LRRFIP2
116	ATP6V1G1	KIAA0324	PTPN1	PLP2	SEC24C	PTGR1
117	AACS	TRIM29	HADH2	CORO1C	AP2M1	PSMA5
118	HPRT1	IL1RA	Q53G17	HINT1	SEC11A	A8K525
119	PSMA6	B4DGF6	PRMT5	FKBP4	LONP1	HADH
120	PLCG1	GRP78	Q7LD69	BICD2	ERF3A	A8K766

121	ADSS	RTCB	TWF1	CES2	Q53GE2	CACYBP
122	CTSD	TIM13	PPT1	HNRPL	MYO6	VASP
123	TPD52	MTAP	SLIRP	NOMO3	COMT	A8K1D2
124	SNCA	YWHAQ	RPL8	IARS2	RPS9	GPD2
125	KRT10	UBE2K	B4DDL4	GPD2	MTAP	ABRACL
126	NAPA	Q59EL2	ATXN10	A8K1D2	EML2	MGST1
127	Q8WZ56	A8K9U0	A7VJC9	GPX1	GALE	TLDC1
128	MTCH2	CLTA	NT5E	HSD17B4	RANGAP1	A8KAQ5
129	RIC8A	SFPQ	RAB13	PKM	Q59ER5	HIST2H2AA3
130	PCK2	Q96GX3	EIF3F	STRAP	RPS4X	CES2
131	B4DF00	CASP1	ARPC5L	E5KLM2	EIF3B	PKP1
132	PDCD10	ARPC1B	Q59GW8	B2R4M6	TOR1AIP1	PIP4K2C
133	B3KS50	Q53FV3	CAPNS1	MAOA	PI4K2A	Q59HH3
134	Q53FC3	GLRX3	MYH10	DCTN1	C1QBP	FH
135	A8KA84	HSPA4L	AP2M1	MRPL49	IGF2BP2	S100A16
136	HARS	GRPEL1	MYO6	AK2	Q8N7G1	RNPS1
137	A7VJC9	DLAT	ETFA	DDX17	PPT1	PDLIM4
138	ASNS	A8KAQ5	PFDN2	PKP1	DSG3	H11
139	EIF2AK2	B7Z792	G3BP2	LRRFIP2	AIM1	C7orf55
140	KRT1	RAB3GAP1	PTPN11	A8K525	CPNE3	HSPA2
141	B7Z4B7	A8K878	RAB5C	ASNS	GLRX5	EWS
142	METAP2	ABRACL	TUBB3	SF3B1	RPS6KA1	Q96GX3
143	SRP19	S100A16	CBX3	AP3B1	ETFA	FTH1
144	FUBP2	PFKP	TIM13	RPS14	B7Z5V3	KRT18
145	TRIP6	B4E1T1	PSAP	LAMC2	B2R694	LETM1

146	FUBP3	BICD2	ZNF185	PRKAR2A	G3BP2	B4DDX2
147	SF3B2	YBX3	HEXB	GCN1L	PCNA	HIST1H2AB
148	B3KNI2	PGRMC1	B4DSQ5	RIC8A	CKAP5	TAF15
149	NDUFS1	Q53F35	HNRNPA0	C19orf10	DEGS1	A8K897
150	NAPRT1	YARS	KRT9	Q8WZ56	CLNS1A	BAX
151	TRMT112	A8K766	B3KT21	PC	KRT8	PSMB5
152	B4DSQ5	HNRNPC	MTCH2	B4DLD4	PRMT5	RPL8
153	CDC37	FAHD2B	I1SRC5	SQSTM1	TACSTD2	SLK
154	FAM83H	A8K1D2	ARHG	EHD2	B2R4R9	RAC2
155	ETFA	TPM4	AKR7A2	HNRPDL	KRT9	SEC61A1
156	Q8NG23	PAI2	ATP6V1G1	Q53FS4	NAPA	MAP2K2
157	PLCB3	A8K525	TPR	ESD	JUP	GMPPB
158	SLIRP	B4DDL4	CSTF2T	KRT15	B2R6K4	TPM4
159	MAOA	HSPA2	LONP1	DLD	Q86U79	SRSF7
160	DBNL	ALYREF	Q53F35	AKAP2	IFITM1	FAHD1
161	IGF2BP2	RPS15	Q53GW1	YBX1	SF3A1	DCTPP1
162	BLVRA	GMPPB	LYPLA1	SAM50	LAMP2	H2AV
163	SYK	Q59E99	ACOT13	Q8NG23	TUBB3	Q53FT8
164	IDH2	S100A2	VAT1	GCS1	PFAS	KTN1
165	COPE	BUB3	EIF2AK2	MRRF	OST48	HIST3H2BB
166	Q59G42	ATOX1	TALDO	MAPK1	PSMC3	SF3B1
167	PSMB3	VPS4A	RTCB	PPL	HARS	HINT1
168	ILK	TMEM43	B2R5V9	Q69YG1	Q54A51	RPS14
169	DNAJB11	CCDC47	FAM83H	HIST3H2BB	IKBIP	YWHAG
170	NAMPT	GRHPR	USP5	GLG1	CDD	B2RDR4

171	Q53HN4	MAP2K2	PPP2R2A	XPO1	A8K3W4	S100A6
172	DNAJC9	PRKCSH	TSTA3	DYNLL2	TCP1	LRRFIP1
173	DSG3	MYG1	A8KA19	A8K9K6	ATP6V1G1	DDX17
174	VPS35	Q53FT8	ARHGEF2	EWS	NCLN	DNAJC9
175	PCNA	B4DRS6	NENF	PAI2	B2RAY1	ATOX1
176	JUP	ESD	ATP6V1E1	EIF2S2	ELAVL1	B3KNN9
177	REEP5	USO1	PPA2	COX6B1	PHP14	EHD2
178	B2R7W4	PIP4K2C	AHSA1	B3KY60	SNRPD1	ACIN1
179	TOR1AIP1	SF3B5	PSMC5	LMOD1	MAT2A	RALA
180	YBX1	A4GYY8	KRT2	L27a	B3KS50	Q53FS4
181	RET1/VSX2	RAC2	A8K9B9	AK1	APT	S100A10
182	GNB2L1	SCAMP2	Q2TNB3	SCAMP3	PFDN2	PLOD1
183	Q59FF0	SEC61A1	PRKDC	SLK	FAS	Q53HN4
184	Q59EK6	DNAJB1	NAA10	B4DDX2	B4DHX4	WASF2
185	Q53GF8	LAMC2	Q7Z518	A8K9U0	B3KNI2	NOMO3
186	SCRN1	UQCRFS1	DDB1	MGST1	RDX	LIS1
187	NACA	CACYBP	RHOA	Q53FN0	Q9UFM8	RPL13
188	TTLL12	PMPCA	RAB35	TUBG2	B4DZ08	HNRPDL
189	B2R694	AK2	TUBB	CACYBP	EVPL	CHCHD6
190	ANXA6	ACTH	GFPT1	LMNA	B4DEA3	EEA1
191	TCP1	ENDOD1	CDK5RAP3	RCN2	IDH2	CASP1
192	B2RAY1	TAF15	FDPS	PPP5C	PDAP1	PSMD14
193	RCN2	PTGR1	NAMPT	AKAP12	CNN3	Q8TAS0
194	TM9SF2	NUDC	MPRIP	FH	STMN1	METAP1
195	ATPO	ESYT2	TRIM25	KIAA0324	BLVRA	PKM

196	BTF3	PDLIM4	OSBPL9	RAB5B	RPS3	GRHPR
197	RPS4X	FKBP2	ANXA8L2	FAHD2B	NACA	NAPRT1
198	NQO1	RCC1	HDGF	HIST1H2AB	RPL18A	Q53F35
199	RPS6KA1	IARS2	U2AF1	RBBP7	MRRF	B3KXH8
200	A8K168	LRRFIP2	Q86TY5	TOM40	NIBAN	PMPCA
201	ANXA5	SH3GLB1	B4DF00	MFGE8	DLAT	FIS1
202	PLEK2	MPRIP	RPL18A	LPP	GNB2L1	CCDC58
203	NT5E	B4DDX2	Q59EK3	PSMD14	CTSD	HLA-A
204	PFDN4	UBAP2L	APT	MVD	ARHG	TOM40
205	PSMD7	EVPL	DARS	HIST1H4H	ATP1A1	DNAJA1
206	HSR1	GLG1	CAPRIN1	PMPCA	FKBP2	GCS1
207	HNRNPH2	B2R9H3		B4DFD5	UBE2D2	PC
208	SF1	FAHD1		NSDHL	Q59GX6	ESD
209	CCT7	Q6NVC0		A8K4T6	LYPLA2	L27a
210	XPO2	B3KY60		SARG	GMPR2	MRPL4
211	PCBP2	HIBADH		Q9H9B7	B7Z9M9	ZYX
212	GLS	MGST1		Q53HQ8	ITGA6	CSRP2
213	Q59GB4	S100A6		AKR1A1	DARS	A4
214	TXNDC17	FTH1		PALLD	CAPNS1	FAHD2B
215	ANXA11	ZYX		TPM4	RET1	HIST1H4H
216	B4DK16	RALA		HLA-B	BUB3	DCTN1
217	AIM1	PALLD		HIST1H1B	NCKAP1	SSBP1
218	ATP1B3	L27a		A8KAQ5	PSMA6	AKR1A1
219	FAS	EIF4B		HSP90AB3P	MYBBP1A	UBAP2L
220	ARPC3	B2R4M6		PLCG1	GOLGB1	A8K9U0

221	Q59EL4	GTF2F1		SBDS	Sep-02	A8K4T6
222	RPL11	Q53FS4		B3KNN9	LDHA	PLCG1
223	ALDH4A1	SNRNP200		SRP19	ITGAV	SRP72
224	APOA1BP	MRPL49		A4	USP5	B4DFD5
225	UGP2	Q53HQ8		RCC1	IFI16	Q8IWR8
226	B4DHX4	EIF6		PSMD7		SRP54
227	NOMO3	E5KLM2		RBBP4		ESYT2
228	ELAVL1	PABPC4		HSPA2		SERBP1
229	RAB6A	A8K4T6		RNPS1		PPP2R2A
230	LTA4H	A4		NAPG		DNPH1
231	CSTF2T	Q8TAS0		NUDC		A8K9U6
232	EIF3M	DHX15		AP3D1		NUDC
233	MYO18A	TUBG2		UBE2D2		PFD5
234	PSMD6	HIST2H2BF		TUBA2		TRADD
235		HIST2H2AA3		MRPL4		SCAMP2
236		MRRF		PON2		PFKL
237		H2AV		PACSIN2		B4DLD4
238		SBDS		HIBADH		RABEP1
239		SERBP1		SSBP1		SBDS
240		B3KXH8		TRADD		LMNA
241		AKAP2		FAM49B		Q53HQ8
242		DNAJA1		HIST1H3A		YBX1
243		LRRFIP1		ENY2		MRPL49
244		SF3B1		CSNK2B		ASNS
245		RABEP1		PTGS2		TUBG2

246		S100A10		ENDOD1		RAB5A
247		HNRPDL		TCEB1		PPL
248		FAM49B		TLDC1		ENDOD1
249		B4DFD5		Q59EE9		Q59EE9
250		PKP1		AP1B1		UNC45A
251		SRP54		SAMHD1		B3KT21
252		SARG		A8K9U6		AK1
253		CRKL		TOP1		B7Z8X5
254		LMNA		PDLIM2		ENY2
255		EWS				NAPG
256		NHP2L1				HIST1H3A
257		HIST1H4H				AP3D1
258		Q9H9B7				B7Z3Q4
259		COPS8				RBBP7
260		HIST3H2BB				B7Z592
261		PPL				MAPK1
262		B4DLD4				B2R6M5
263		HINT1				TUBA2
264		SLK				TOP1
265		EHD2				SAMHD1
266		PPP5C				SPRR1B
267		GCS1				PDLIM2
268		RNPS1				
269		H11				
270		S100A8				

271		B7Z6Y3				
272		TLDC1				
273		ENY2				
274		CHCHD6				
275		B3KNN9				
276		FAM3C				
277		DNPH1				
278		B2R608				
279		AK1				
280		HIST1H2AB				
281		B2R6M5				
282		HSP90AB3P				
283		TUBA2				
284		Q59EE9				
285		HIST1H3A				
286		MAPK1				
287		RAB5A				
288		A8K9U6				
289		PACSIN2				
290		SAMHD1				
291		AP1B1				
292		RBBP7				
293		CSNK2B				
294		HIST1H1B				
295		B7Z3Q4				

296		B3KT21				
297		AP3D1				
298		TOP1				
299		PDLIM2				
300		SPRR1B				
301		NAPG				
